# Molecular Networking Reveals Two Distinct Chemotypes in Pyrroloiminoquinone-Producing *Tsitsikamma favus* Sponges

**DOI:** 10.3390/md17010060

**Published:** 2019-01-16

**Authors:** Jarmo-Charles J. Kalinski, Samantha C. Waterworth, Xavier Siwe Noundou, Meesbah Jiwaji, Shirley Parker-Nance, Rui W. M. Krause, Kerry L. McPhail, Rosemary A. Dorrington

**Affiliations:** 1 Department of Biochemistry and Microbiology, Rhodes University, PO Box 94, Grahamstown 6140, South Africa; samche42@gmail.com (S.C.W.); X.SiweNoundou@ru.ac.za (X.S.N.); m.jiwaji@ru.ac.za (M.J.); shirley@saeon.ac.za (S.P.-N.); 2 Division of Pharmaceutical Sciences, University of Wisconsin-Madison, Madison, WI 53705, USA; 3 South African Environmental Observation Network, Elwandle Node, Nelson Mandela University Ocean Sciences Campus, Summerstrand, Port Elizabeth 6001, South Africa; 4 Department of Chemistry, Rhodes University PO Box 94, Grahamstown 6140, South Africa; r.krause@ru.ac.za; 5 Department of Pharmaceutical Sciences, College of Pharmacy, Oregon State University, Corvallis, OR 97331, USA; kerry.mcphail@oregonstate.edu

**Keywords:** *Latrunculiidae*, makaluvamine Q, damirone, pyrrolo-*ortho*-quinone, discorhabdin, tsitsikammamine, HR-ESI-LC-MS/MS, pyrroloquinoline, GNPS

## Abstract

The temperate marine sponge, *Tsitsikamma favus*, produces pyrroloiminoquinone alkaloids with potential as anticancer drug leads. We profiled the secondary metabolite reservoir of *T. favus* sponges using HR-ESI-LC-MS/MS-based molecular networking analysis followed by preparative purification efforts to map the diversity of new and known pyrroloiminoquinones and related compounds in extracts of seven specimens. Molecular taxonomic identification confirmed all sponges as *T. favus* and five specimens (chemotype I) were found to produce mainly discorhabdins and tsitsikammamines. Remarkably, however, two specimens (chemotype II) exhibited distinct morphological and chemical characteristics: the absence of discorhabdins, only trace levels of tsitsikammamines and, instead, an abundance of unbranched and halogenated makaluvamines. Targeted chromatographic isolation provided the new makaluvamine Q, the known makaluvamines A and I, tsitsikammamine B, 14-bromo-7,8-dehydro-3-dihydro-discorhabdin C, and the related pyrrolo-*ortho*-quinones makaluvamine O and makaluvone. Purified compounds displayed different activity profiles in assays for topoisomerase I inhibition, DNA intercalation and antimetabolic activity against human cell lines. This is the first report of makaluvamines from a *Tsitsikamma* sponge species, and the first description of distinct chemotypes within a species of the *Latrunculiidae* family. This study sheds new light on the putative pyrroloiminoquinone biosynthetic pathway of latrunculid sponges.

## 1. Introduction

Sponges of the family *Latrunculiidae* produce highly condensed and often brominated alkaloids such as makaluvamines, discorhabdins, and tsitsikammamines, collectively known as pyrroloiminoquinones [1,2]. These compounds display a broad range of biological activities, including cytotoxic [3,4,5,6,7], antitumor [8], antimicrobial [9,10], antiplasmodial [11], and antioxidant activities [12], as well as inhibition of topoisomerase I [7,13] and II [6,8], calcineurin, CPP32 [5], and cholinesterase [14]. Recently, pyrroloiminoquinones have attracted a surge of renewed interest as potential anticancer drug leads. Several makaluvamines, discorhabdins, and synthetic analogs have shown promising results in anticancer assays [3,4,15,16] and as inhibitors of the formation of the cancer-related HIF-1α/p300 enzyme complex [17].

The temperate coastal waters of the South African Agulhas Bioregion are home to several latrunculid sponge species, including *Tsitsikamma favus* and *Tsitsikamma pedunculata* [1]. Previous investigations reported the isolation of tsitsikammamines and discorhabdins of the C and V series from *T. favus*, while *T. pedunculata* yielded only C and V series discorhabdins [7,18]. The first tsitsikammamines were discovered by Hooper et al. in 1996 [18], who isolated tsitsikammamines A and B alongside 14-bromodiscorhabdin C and 14-bromo-3-dihydrodiscorhabdin C from *T. favus*. Subsequently, tsitsikammamine C was isolated from an Australian *Zyzzya* sp. [11] and recently tsitsikammamine A and 16,17-dehydrotsitsikammamine A were reported from an Antarctic representative of the species, *Latrunculia biformis*, by Li et al. [19]. Reports of discorhabdins of the V series are exclusive to the genus *Tsitsikamma*, while brominated derivatives or analogs of the discorhabdin C series have been isolated from species belonging to the genera *Tsitsikamma* [7,18], *Strongylodesma* [7], *Latrunculia* [9,14,20,21], *Sceptrella* [22], and *Batzella* [5].

The pyrroloiminoquinone biosynthetic pathway in latrunculid sponges is proposed to proceed from simple pyrroloiminoquinones and pyrrolo-*ortho*-quinones, such as makaluvamines and damirones, to tsitsikammamines and discorhabdins [23,24]. For discorhabdin B, phenylalanine is incorporated, as demonstrated by radio-isotope labeling experiments on tissue slices of a New Zealand *Latrunculia* species [23]. The pyrroloiminoquinone core itself is proposed to originate from tryptophan [24], which is also the initial precursor in the biosynthetic pathway leading to the related pyrroloquinoline, lymphostin, produced by the marine bacterium *Salinispora* sp. [25]. This, together with the conservation of dominant bacterial symbionts in the microbiomes of multiple pyrroloiminoquinone-producing *Tsitsikamma* species [26], has led to the suggestion that the biogenetic origin of pyrroloiminoquinones in latrunculid sponges may be microbial. Whether or not this is indeed the case remains to be determined. In this study, we set out to gain new insight into pyrroloiminoquinone biosynthesis, focusing on the secondary metabolite ensemble produced by *T. favus* sponges and targeting pyrroloiminoquinones for isolation and bioactivity assays.

## 2. Results

### 2.1. Taxonomic Identification of Sponge Specimens and Microbial Community Profiles

**Figure 1:**
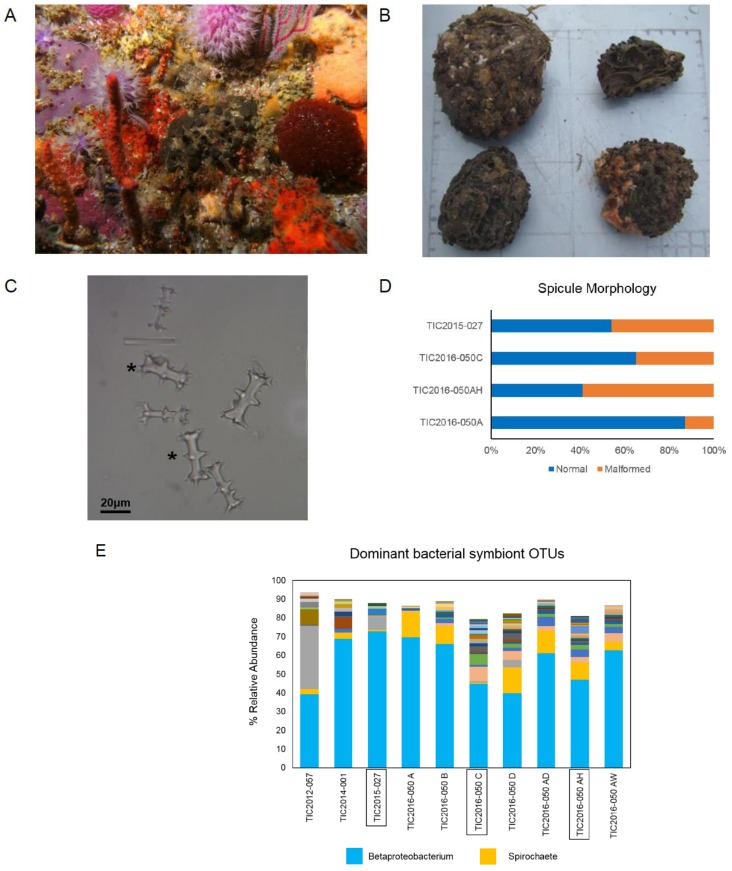
Taxonomic identification of sponges and 16S rRNA gene sequence profiling of sponge-associated bacterial. (**A**) In situ image of a live specimen collected from Evans Peak and (**B**) four representative specimens from the TIC2016-050 collection; (**C**) Skeletal components of *T. favus* specimen TIC2016-050AH with standard spicules indicated with asterisks (*); (**D**) Relative abundance of malformed spicules in four sponge specimens. Spicules mounted on permanent slide and first 100 spicules for each specimen categorized; (**E**) Dominant bacterial OTUs in sponge-associated microbiomes derived from 16S rRNA gene sequencing.

More than fifty sponge specimens were collected by SCUBA or Remotely Operated Vehicle (ROV) from Evan’s Peak reef, Algoa Bay, South Africa, including TIC2015-027 in September 2015 and TIC2016-050A, TIC2016-050B, TIC2016-050C, TIC2016-050D, TIC2016-050AD, TIC2016-050AH, and TIC2016-050AW, from a single location, in March 2016. Initial visual identification of these sponges as *T. favus* specimens (Figure 1A,B) was confirmed by 28S rRNA sequencing (Supplementary Table S.1). Subsequent spicule analysis however revealed an unusually high abundance (35–59%) of malformed spicules with irregular tubercles, (Figure 1C) present in three specimens (TIC2015-027, TIC2016-050C, and TIC2016-050AH) compared with the type specimens, represented by TIC2016-050A with 13% malformed spicules (Figure 1D).

The skeletal abnormalities observed in TIC2015-027, TIC2016-050C, and TIC2016-AH did not correlate with significant shifts in sponge-associated microbial communities as determined by 16S rRNA gene sequencing. For comparison, 16S rRNA data from previous work is included (TIC2012-057 and TIC2014-001 in Matcher et al. [26]). The associated microbiota of all sponge specimens was dominated by a conserved Betaproteobacterium Operational Taxonomic Unit (OTU) and a second, conserved Spirochetes OTU was also present but in varying relative abundance (Figure 1E). These OTUs were previously found to be conserved in *Tsitsikamma* sponge-associated microbial communities [26]. Furthermore, 18S rRNA gene sequence profiling (data not shown) and a comparative analysis of the metagenomes of TIC2015040A and TIC2015050C (S.C. Waterworth and R.A. Dorrington, unpublished data) failed to reveal significant differences between the two morphotypes of *T. favus* sponges leading to the conclusion that these differences were unlikely to be due to a pathogenic microbial infection.

### 2.2. Purification and Structure Elucidation

**Figure 2:**
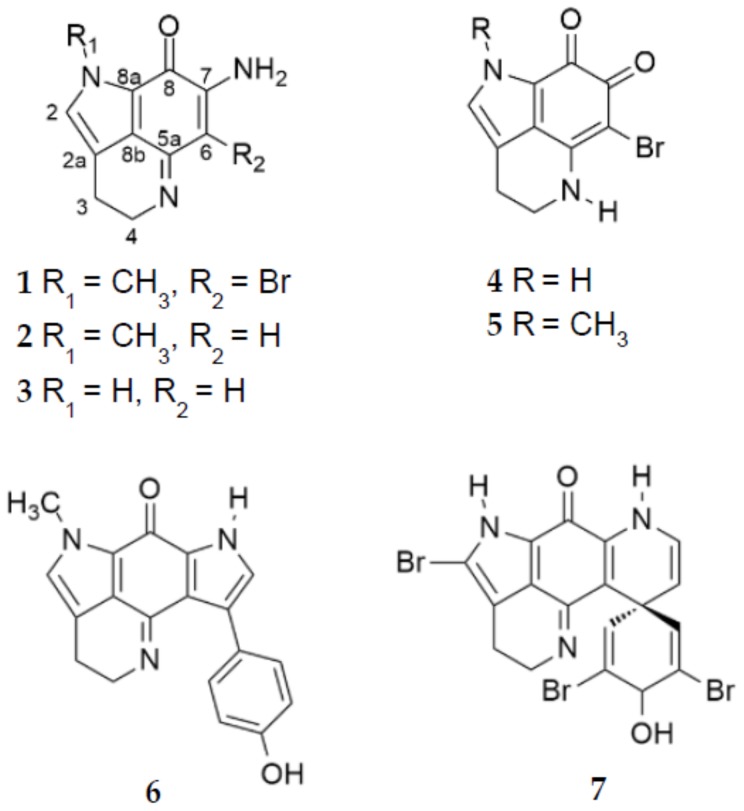
Chemical structures of the isolated makaluvamines Q (**1**), A (**2**), I (**3**), and O (**4**), makaluvone (**5**), tsitsikammamine B (**6**) and 14-bromo-7,8-dehydro -3-dihydrodiscorhabdin C (**7**).

Initial UV-HPLC profiling of organic extracts from *T. favus* collected in September 2015 and June 2016 revealed that specimens with increased spicule malformation also exhibited chromatographic profiles distinct from specimens without spicule malformation (Supplementary Figure S.2). Preparative chromatography efforts focused on one specimen of each chemotype of *T. favus* sponge (TIC2016-050A, type I; TIC2016-050C, type II). Organic extracts (DCM-MeOH 2:1, *v*/*v*) were prepared from frozen sponge material and subjected to Sephadex LH-20 chromatography or reversed-phase solid phase extraction (RP-SPE), followed by RP semi-preparative HPLC purification. Exhaustive chromatography resulted in the isolation of seven pure compounds (Figure 2) identified by 1D and 2D NMR spectroscopy (^1^H, ^13^C, DEPT, ^1^H-^1^H COSY, HSQC, and HMBC; Supplementary Figures S.7.1 to S.7.34) and HRMS (Supplementary Figures S.5.1 to S.5.7) as: makaluvamine Q (**1**), makaluvamine A (**2**) [6], makaluvamine I (**3**) [27], makaluvamine O (**4**) [28], makaluvone (**5**) [6], tsitsikammamine B (**6**) [18], 14-bromo-3-dihydro-7,8-dehydrodiscorhabdin C (**7**) [7]. Makaluvamine Q (**1**) represents the first example of a brominated *N*-methylated makaluvamine.

Positive mode HRESIMS analysis of makaluvamine Q (**1**) produced paired (1:1) isotope peaks at *m*/*z* 280.0076 and 282.0058, indicative of monobromination and a molecular formula of C_11_H_10_N_3_OBr, for eight double bond equivalents (Supplementary Figure S.5.1). The IR spectrum displayed a carbonyl absorbance (1732 cm^−1^; Supplementary Figure S.6). The ^13^C NMR spectrum (Table 1) (Supplementary Figures S.7.2 and S.7.3) exhibited eleven resonances, including one methyl, two methylenes, one methine, and seven non-protonated, sp^2^-hybridized carbons. One of latter could be assigned as the carbonyl carbon (δ_C_ 166.7), and of the remaining six sp^2^-hybridized ^13^C signals (δ_C_ 82.1 −156.4), the one at δ_C_ 82.1 was assigned as a brominated (C-6). The remaining signals were consistent with a methine (δ_C_ 132.4) and two methylene (δ_C_ 19.4 and 44.7) carbons.

The ^1^H NMR spectrum for **1** (Table 1, Supplementary Figure S.7.1) exhibited two mutually coupled triplets at δ_H_ 2.92 (H-3, t, 7.7 Hz, 2H) and 3.88 (H-4, t, 7.7 Hz, 2H), confirming the presence of two methylenes in **1**. A deshielded methine singlet (δ_H_ 7.12, s) was characteristic of a pyrroloiminoquinone core structure [29]. An additional 3H deshielded singlet resonating at δ_H_ 3.92, (N-CH_3_, s) was consistent with an *N*-methyl group. In the HMBC spectrum, correlations (Supplementary Figure S.7.6) were observed between H-3 and C-2, C-2a, C-4, and C-8b, as well as H-4 and C-2a, C-3 and C-5a. The methyl group was located at *N*-1, based on ^3^*J*_CH_ correlation from H-2 to the methyl carbon. The absence of a proton signal between δ_H_ 5–6 (as observed for non-brominated **2**) is consistent with bromination at C-6.

The COSY spectrum displayed couplings (Supplementary Figure S.7.4) only between protons H_2_-3 and H_2_-4. HSQC data together with COSY and HMBC spectra, as well as comparison with literature data of the non-methylated analog makaluvamine N [29], allowed assignment of the remaining ^13^C signals. Thus, the structure of **1** was assigned and the compound designated as the new makaluvamine Q.

### 2.3. Molecular Networking and Dereplication

To further elucidate the diversity of pyrroloiminoquinone production in *T. favus*, we acquired HRESI-LC-MS/MS data on organic extracts of seven specimens (TIC2016-050A, B, C, D, AD, AH, and AW), collected in March 2016. For data analysis purposes, we utilized a molecular networking approach using MZmine 2.34 to pre-process data [30], followed by molecular networking analysis on the Global Natural Product Social (GNPS; www.gnps.ucsd.edu) platform [31].

**Figure 3:**
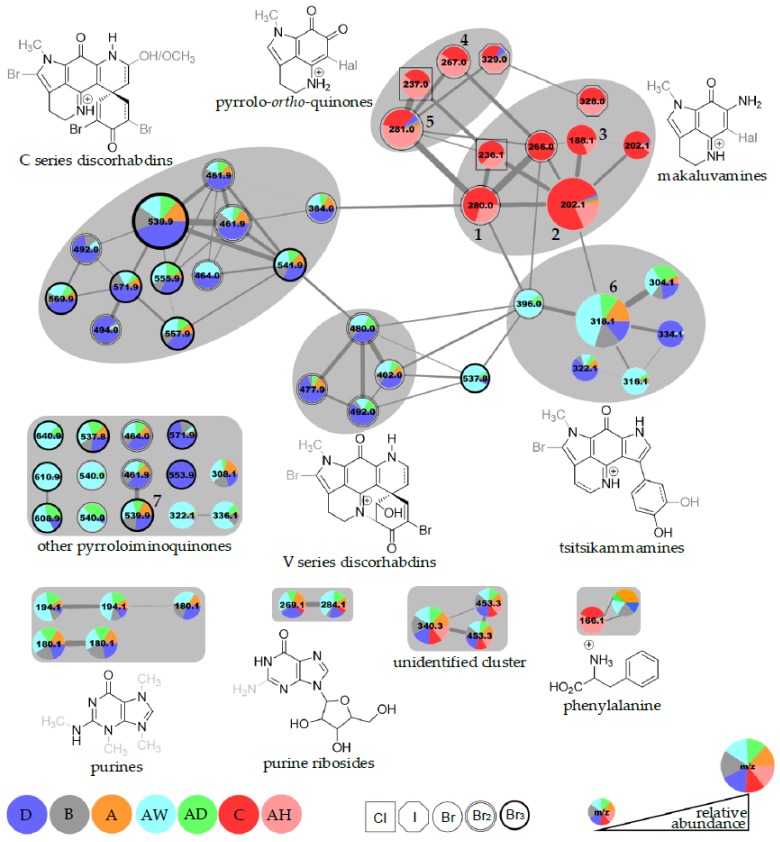
Annotated molecular network of *T. favus* organic extracts. Each chromatographic feature is represented by a single node labeled in Da according to monoisotopic mass, edge width increases with spectral similarity. Contributions to the pie charts by each sample correspond to chromatographic peak areas with samples (2016-050-A, B, C, D, AD, AH, and AW) distinguished by color and node size representing the total chromatographic peak area summed over all samples. The nature and degree of halogenation is indicated by node border shape. Node identities corresponding to isolated compounds are annotated by number as in the text. Hypothesized molecular core structures for sub-networks are given with proposed sites of substitution drawn in grey.

The resulting molecular network (Figure 3) was visualized in Cytoscape 3.51 and initial inspection of relative peak area contributions to the network nodes revealed that the ensembles of natural products produced by the two *T. favus* chemotypes were strikingly different. While chemotype I produced many compounds identified as discorhabdins and tsitsikammamines, extracts of chemotype II were dominated by the presence of makaluvamines and related pyrrolo-*ortho*-quinolines, with only trace detection of tsitsikammamines and no detectable presence of any discorhabdins.

Most compounds detected in chemotype II specimens form a distinct sub-network with little contribution from chemotype I samples and the large node at *m*/*z* 202.1 representing makaluvamine A (**2**). Other verified nodes within this sub-network are makaluvamine Q (**1**, *m*/*z* 280.0), makaluvamine I (**3**, *m*/*z* 188.1), makaluvamine O (**4**, *m*/*z* 267.0), and makaluvone (**5**, *m*/*z* 281.0). Dereplication efforts propagated from these verified nodes allowed for putative identification of makaluvamine C [6] or a new isomer (not **2**, small 202.1 Da node), as well as isobatzelline C (*m*/*z* 236.1) [32] and batzelline C (*m*/*z* 237.0) [33], which are the chlorinated analogs of **1** and **5**, respectively. The two remaining nodes at *m*/*z* 328.0 and 329.0 were identified as the iodinated analogs of **1** and **5** and represent the first evidence of iodinated pyrroloiminoquinones.

The neighboring tsitsikammamine sub-network is almost exclusively comprised of chemotype I samples and dominated by tsitsikammamine B (**6**, *m*/*z* 318.1). Other nodes in this sub-network were putatively identified as tsitsikammamine A (*m*/*z* 304.1) [7], 16,17-tsitsikammamine B (*m*/*z* 316.1), a brominated analog of tsitsikammamine B (*m*/*z* 396.0), as well as hydroxylated analogs of tsitsikammamine A and 16,17-dehydrotsitsikammamine B (*m*/*z* 322.1 and 334.1, respectively). 

The node representing the brominated tsitsikammamine B analog (*m*/*z* 396.0) links to a minor sub-network of four nodes, which is itself linked to the major discorhabdin sub-network. Taking into consideration the derived molecular formulas, the relatively short retention times and previous studies on *T. favus* [7,18], we hypothesize that this sub-network of four nodes represents hexa-cyclic mono- and dibrominated discorhabdin V-like compounds and that the neighboring node at *m*/*z* 537.8 represents a hexa-cyclic 14-bromo-discorhabdin C analog. The fragment intensities observed in the MS^2^ spectra for these nodes were very low, indicative of the high structural stability of these compounds.

The isolated 14-bromo-7,8-dehydro-3-dihydrodiscorhabdin C (**7**) at *m*/*z* 539.9 was grouped among the poorly or non-clustered pyrroloiminoquinones (“other pyrroloiminoquinones” in Figure 3), linking only to a node matching 7,8-dehydro-3-dihydrodiscorhabdin C (*m*/*z* 461.9). Water loss was observed in the fragmentation spectra of both nodes and we suggest that the presence of a C-3 hydroxyl may be responsible for the lack of connectivity with the main network.

The MS^2^ spectra of the nodes of the major discorhabdin sub-network, show much less pronounced water loss, implying that the structures of this sub-network are based on a discorhabdin C skeleton containing the α-bromoacryloyl moiety and variable substituents. The major node in this sub-network at *m*/*z* 539.9 matches 14-bromodiscorhabdin C [7], yet double bonds could not be located conclusively. Dibrominated discorhabdin C-like analogs are observed at *m*/*z* 461.9 and 464.0. Furthermore, compounds representing hydroxylated derivatives of 14-bromodiscorhabdin C or an isomer are indicated for the *m*/*z* 555.9 and 557.9 nodes, while methoxylated analogs of di- and tribrominated discorhabdin C type compounds are assigned for *m*/*z* 492.0, 494.0, 569.9, and 571.9. The major discorhabdin sub-network and the makaluvamine sub-network are connected through a node with *m*/*z* 384.0, representing a compound containing a single bromine atom and matching discorhabdin E and G.

**Figure 4:**
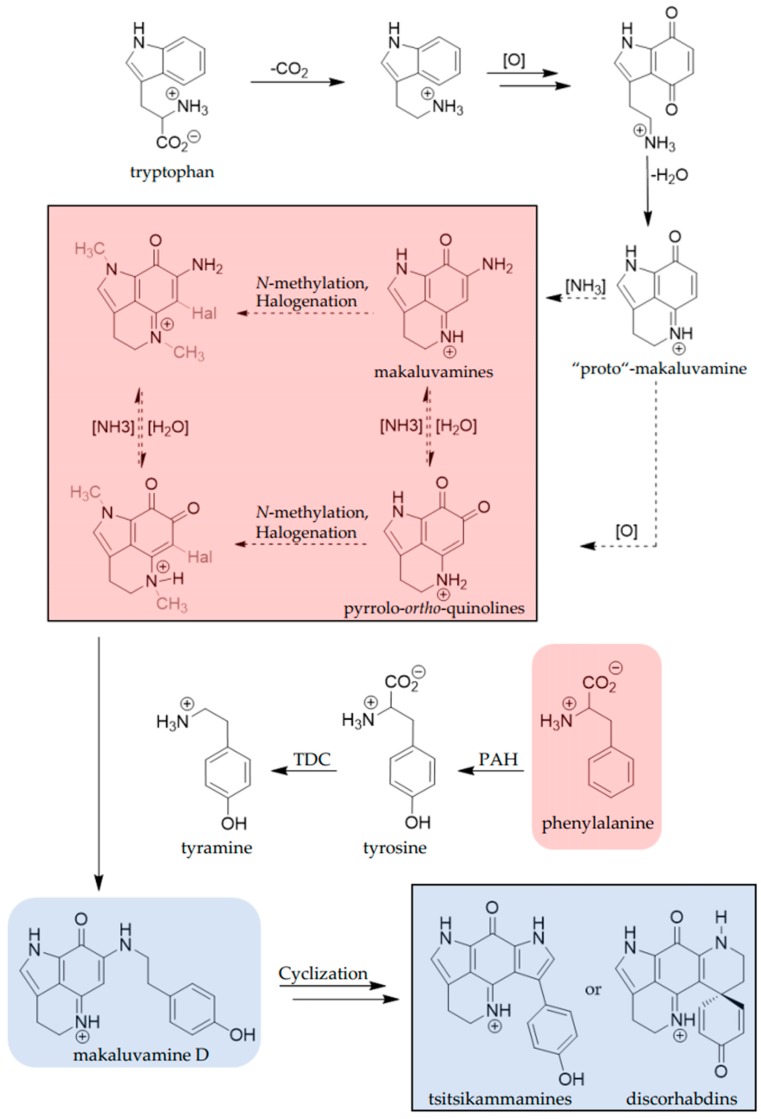
Proposed biogenesis of pyrroloiminoquinones in marine sponges originating from tryptophan. Dashed arrows represent possible alternative reaction pathways. Shaded backgrounds indicate structures predominantly identified in organic extracts of *T. favus* type I in blue and type II in red. Variable substituents are drawn in grey. TDC: tyrosine decarboxylase; PAH: phenylalanine hydroxylase. The proposed biosynthetic pathway in *T. favus* has been adapted from various authors [1,2,23,24] and modified for relevance to this study.

The remaining nodes identified as pyrroloiminoquinones are grouped with the node verified as 14-bromo-7,8-dehydro-3-dihydrodiscorhabdin C (**7**). The molecular formulas determined for the nodes at *m*/*z* 322.1 and *m*/*z* 336.1 match the branched makaluvamines E [6] and M [27], respectively. Furthermore, the node at *m*/*z* 308.1 was only detected in chemotype I and putatively identified as makaluvamine D [6], which is considered to be an intermediate structure in the formation of discorhabdins and tsitsikammamines from simple pyrroloiminoquinones or pyrrolo-*ortho*-quinones (Figure 4). Unspecified tribrominated pyrroloiminoquinone conjugates were identified at *m*/*z* 640.9, 610.9 and 608.9, corresponding to molecular ion formulas of [C_22_H_19_Br_3_N_4_O_4_ + H]^+^, [C_21_H_17_Br_3_N_4_O_3_ + H]^+^ and [C_21_H_15_Br_3_N_4_O_3_ + H]^+^, respectively. Other noteworthy compounds are represented by the connected pair of nodes at *m*/*z* 540.0, corresponding to dibrominated isomers with a molecular ion formula of [C_20_H_19_Br_2_N_3_O_5_ + H]^+^ and possibly representing discorhabdin C conjugates. 

Three additional minor clusters were identified as purines and purine ribosides. Interestingly, only chemotype I contributed to the two clusters containing nodes with *m*/*z* 180.1 and 194.1, and the best matches for ion formulas are [C_7_H_9_N_5_O + H]^+^ and [C_8_H_11_N_5_O + H]^+^. These ions were putatively identified as di- and trimethylated guanine derivatives, based on reports of the isolation of 3,7-dimethylguanine from the pyrroloiminoquinone-producing sponges *Zyzzya fuliginosa* and *Latrunculia purpurea* [8,34]. The nodes of the neighboring cluster at *m*/*z* 269.1 and 284.1 were identified as purines by spectral matching to hypoxanthine and guanine, with cosine values of 0.93 and 0.85, respectively (Supplementary Figures S.3.1 and S.3.2). Taking into consideration a precursor mass offset of +132 Da to the purines for both nodes, we believe that these nodes represent the respective purine ribosides, inosine, and guanosine. Both compounds appear in all samples from both chemotypes, albeit more abundantly in chemotype I compared with chemotype II. The remaining cluster with nodes at 340.3 Da and 453.3 Da, appears to be equally abundant in both chemotypes and the nodes were not identified successfully.

Finally, the node at *m*/*z* 166.1 (Figure 3, bottom right) was identified as phenylalanine by spectral matching to a MassBank entry (PB000409) through GNPS, with a cosine score of 0.92 (Supplementary Figure S.3.3). Phenylalanine was detected in all samples but with much higher relative abundance in chemotype II extracts.

### 2.4. Evaluation of Biological Activity

We tested the isolated compounds for inhibition of topoisomerase I catalyzed DNA decatenation, DNA intercalation ability and antimetabolic activity vs human HEK293 (Supplementary Figure S.4.1) and HeLa cells. All compounds displayed some topoisomerase I inhibition and DNA intercalation, with the pyrrolo-*ortho*-quinolines **4** and **5** being the most effective topoisomerase I inhibitors, while exhibiting the lowest DNA affinity (Table 2 and Supplementary Figures S.4.2 and S.4.3). The same two compounds had no significant adverse effect on the metabolic activity of either human cell line at the tested concentrations. In contrast, the isolated makaluvamines **1**–**3** elicited a decrease in metabolic activity of HeLa cells, with the new makaluvamine Q (**1**) proving most active at the tested concentration, while also exhibiting the highest DNA affinity. Activity against HEK293 cells followed a similar trend: **1**–**3** and discorhabdin **7** inhibited metabolic activity to below 20% viability at 50 μM, while the bis-pyrroloiminoquinone **6** exhibited weak activity even at such high concentrations. Only compound **1** exhibited an inhibitory effect on cell viability at a concentration of 5 μM.

## 3. Discussion

In this study, we characterized the secondary metabolites of *T. favus* sponges, isolating one new and six known pyrroloiminoquinone compounds. This is the first report of makaluvamines from a *Tsitsikamma* species. The seven isolated compounds displayed varying activities in biological assays and it became evident that topoisomerase I inhibition does not correlate with the DNA affinity of the compounds. This suggests that for these compounds, topoisomerase I inhibition does not require DNA intercalation. Molecular networking analysis of the secondary metabolites of seven *T. favus* specimens resulted in the putative identification of 48 pyrroloiminoquinones, several of which are new and as yet uncharacterized. The data also revealed the existence of two distinct *T. favus* chemotypes, which predominantly produce both discorhabdins and tsitsikammamines (chemotype I) or makaluvamines (chemotype II).

The prevalence of different chemotypes in marine sponges, particularly linked to geographic location, is not new, and even latrunculid sponges have been reported to exhibit some location-dependent variation in pyrroloiminoquinone production [8,35]. Nonetheless, the striking intra-species variation in secondary metabolite production observed in this study for *T. favus* specimens from the same collection site has not been reported before. This phenomenon is not confined solely to the 2015 and 2016 collections: we have found that 8 out of 26 specimens collected from the same site in July 2018 displayed chemotype II (data not shown). We have yet to observe chemotype II specimens in collections from other reefs within Algoa Bay, so it remains to be seen whether chemotype II *T. favus* sponges are peculiar to the Evans Peak reef.

The two *T. favus* chemotypes are characterized either by the presence of tsitsikammamines and discorhabdins as in type I, or by makaluvamines and increased relative abundance of phenylalanine in type II. This is likely to be related to the pyrroloiminoquinone biosynthetic pathway. Phenylalanine is known to be involved in discorhabdin biosynthesis [23] and has been suggested to react with makaluvamines, via its *p*-hydroxylated, decarboxylated derivative tyramine, to form structurally more complex pyrroloiminoquinones (Figure 4).

The biogenesis of the pyrroloiminoquinone core is proposed to begin with decarboxylation of tryptophan, followed by oxidation and condensation to give a hypothetical “proto”-makaluvamine. From this precursor, the pathway is proposed to proceed by either oxidation or amination to pyrrolo-*ortho*-quinones or makaluvamines, which are then converted into their methylated and halogenated analogs.

The next step in the biosynthetic pathway is believed to be the condensation of tyramine and one of the pyrrolo-*ortho*-quinolines or makaluvamines to give makaluvamine D. This putative key intermediate between simple and complex pyrroloiminoquinones was identified only in *T. favus* chemotype I (Figure 3; node 308.1 Da) and it is uncertain whether its biosynthesis is facilitated from tyramine and pyrrolo-*ortho*-quinolines, tyramine and unbranched makaluvamines, or even tyramine and the “proto”-makaluvamine (Figure 4). The accumulation of phenylalanine and correlating deficiency of tsitsikammamines and discorhabdins suggest that this step is blocked in chemotype II and furthermore corroborates the phenylalanine incorporation in tsitsikammamine/discorhabdin biosynthesis. In chemotype I sponges, the cyclization of makaluvamine D or an activated derivative is assumed to lead to tsitsikammamines or discorhabdins, or both.

The precursor tyramine is derived from tyrosine by decarboxylation catalyzed by a tyrosine decarboxylase [36]. Tyrosine is synthesized de novo only in plants and bacteria, while animals use phenylalanine to produce tyrosine in a reaction catalyzed by phenylalanine hydroxylase (PAH) [37,38]. The activity of either of these enzymes could be inhibited or absent in the chemotype II holobionts, resulting in the build-up of phenylalanine. Interestingly, PAH is biopterin-dependent and biopterins are biosynthesized from the purine precursor guanosine triphosphate [39], which in turn is derived from inosine monophosphate [40]. The observation of increased purine and purine riboside levels in the chemotype I metabolomes, may thus be related to the differences in pyrroloiminoquinone and phenylalanine production observed between chemotypes. Biosynthetic phenylalanine incorporation into discorhabdin B in sponge tissue slices is unaffected by the presence of broad spectrum antibiotics, suggesting that sponge cells are the biogenetic origin of discorhabdins [23]. This leads us to speculate that the two chemotypes may result from alterations in sponge-related metabolic pathways.

The isolation of makaluvamine A from a myxomycete culture [41] and the production of the structurally related lymphostin by an actinobacterium [42], lend support to a potential microbial origin for makaluvamines. It is tempting to speculate on a combined effort by the sponge host and selected associated microbial symbionts, resulting in pyrroloiminoquinone production by latrunculid sponges. If this model were correct, then the makaluvamines and pyrrolo-*ortho*-quinones are produced microbially and then converted to discorhabdins and tsitsikammamines by the sponge. We are currently engaged in the analysis of metagenomic sequence data derived from chemotype I and II *T. favus* sponges and other latrunculid species to identify potential microbial biosynthetic gene clusters.

## 4. Materials and Methods

### 4.1. General Experimental Procedures

IR spectra were recorded on a Perkin-Elmer Spectrum 100 FTIR spectrometer (Perkin-Elmer, Shelton, CT, USA). NMR spectra were obtained on a Bruker Avance DRX-600 MHz spectrometer (Bruker, Rheinstetten, Germany) using methanol-*d*_4_ (Merck Millipore, Johannesburg, South Africa) or DMSO-*d*_6_ (Sigma-Aldrich, Johannesburg, South Africa) as solvents. The MS analyses were performed on a Bruker Compact QToF mass spectrometer using an electrospray ionization probe (Bruker, Bremen, Germany). Organic extract analysis was carried out in LC-MS/MS mode with a Dionex UHPLC (Thermo Fisher Scientific, Sunnyvale, CA, USA) equipped with a Kinetex polar C-18 column (3.0 × 100 mm, 2.6 μm; Phenomenex, Torrance, CA, USA) at a flowrate of 0.300 mL/min using mixtures of water and acetonitrile (MeCN), both adjusted with 0.1% formic acid (FA). The water was prepared using a MilliQ filtration system (Merck Millipore, Johannesburg, South Africa) and LC-MS grade acetonitrile was purchased from Merck Millipore (Johannesburg, South Africa); formic acid was acquired from Sigma-Aldrich (Johannesburg, South Africa). Preparative column chromatography was performed using Sephadex LH-20 purchased from Sigma Aldrich (Johannesburg, South Africa). For solid phase extraction, Waters 10 g C-18 cartridges were used and semi-preparative HPLC was carried out on a Waters 1525 binary pump system equipped with a Waters model 2487 dual λ absorbance detector and an XBridge C18-RP-Shield column (10 × 150 mm, 5 µm; Waters, Milford, MA, USA) using the same mobile phase components as in the LC-MS analysis.

### 4.2. Sample Collection, Taxonomic Identification, and 16S rRNA Amplicon Analysis

Specimen TIC2015-027 was collected by SCUBA at Evans Peak reef, South Africa from a depth of 30 m (−33.8455, 25.81663) in September 2015. All other specimens of *T. favus* were collected by SCUBA from depths between 15 and 20 m at Evans Peak, South Africa (−33,84548, 25.81663) in June 2016. Sponges were identified morphologically, both by general characteristic of the sponge, distribution of oscula and porefields, partitioning of the choanosome, and the shape of the spicules, and are deposited in the South African Institute for Aquatic Biodiversity (SAIAB) in Grahamstown, South Africa under Accession numbers SAIAB207230 (TIC2015-027) and SAIAB207192 (TIC2016-050 series). Other sponge specimens used in this study, TIC2012-057 and TIC2014-001, are accessioned at SAIB as SAIAB207187 and SAIAB207189, respectively.

Sponge gDNA was isolated as described previously [43]. Morphological identification was sequence analysis of the D3-D5 region of the sponge 28S rRNA gene. The 28S rRNA gene fragment was PCR amplified using primer pair RD3a (5′-GAC CCG TCT TGA AAC ACG A-3′) and RD5b2 (5′-ACA CAC TCC TTA GCG GA-3′). PCR amplificationwas carried out as described before [43] and amplicons were ligated into the pGEM-T Easy vector (Stratagene) and the insert sequence determined by Sanger sequencing. 

Assessment of the sponge associated bacterial communities was performed through amplification of the 470 bp hypervariable region between V4 and V5 of the 16S rRNA gene using primer pair E517F (5′-CAG CAG CCG CGG TAA-3′) and E969-984 (5′-GTA AGG TTC YTC GCG T-3′). PCR amplification of 16S rRNA gene fragments was performed in reactions containing 0.3 μM primers, 0.3 μM dNTPs, 1X Buffer with MgCl2, and 1 U Kapa HiFi Taq polymerase with 30 ng DNA template. Thermal cycling parameters employed were as follows: Initial denaturation at 95 °C for 5 min followed by amplification with 35 cycles at 94 °C for 30 s, 45 °C for 20 s, 72 °C for 1 min, followed by a final elongation step of 72 °C for 10 min. Resultant amplicons were gel purified using the Isolate II PCR and Gel kit from Bioline (Cat. No. BIO-52060). Amplicon libraries were further processed and sequenced using the Illumina MiSeq sequencing platform.

Amplicon library sequence datasets were curated using Mothur software v.1.35.1 [44]. Sequences less than 250 nts in length, containing ambiguous bases and/or homopolymeric runs greater than 8 nts were discarded. Sequence chimeras were identified using the VSEARCH algorithm [45] and removed from the respective datasets. Sequences were classified using the Naïve Bayesian classifier against the Silva bacterial database (Version 132) and then scored as the relative percentage of reads per sample dataset. A distance matrix (cut-off of 0.05) was generated in Mothur and used to cluster the sequence reads into Operational Taxonomic Units (OTUs) at distance values of 0.03 (species level). OTUs with a relative percentage lower than 0.5% were excluded from the analysis of dominant bacteria. The raw sequence data has been deposited in the NCBI SRA database under the accession number PRJNA508092.

### 4.3. Data Acquisition and Processing

Samples were prepared from dried sponge extracts at 1 mg/mL in methanol. The mobile phase program was set to follow a step gradient, with constant ramping between segments and proceeded as follows: 0–5 min (H_2_O–MeOH–FA, 95:5:0.1, *v*/*v*/*v*), 10–15 min (H_2_O–MeOH–FA, 90:10:0.1, *v*/*v*/*v*), 20–25 min (H_2_O–MeOH–FA, 85:15:0.1, *v*/*v*/*v*), 30–35 min (H_2_O–MeOH–FA, 80:20:0.1, *v*/*v*/*v*), 40–45 min (H_2_O–MeOH–FA, 60:40:0.1, *v*/*v*/*v*). Injection volumes were 10 μL. The positive mode MS source parameters were set as follows; End Plate Offset 500 V, Capillary Voltage 4500V, Nebulizer pressure 3.0 bar, Dry Gas flow 9.0 L/min, Dry Temperature 220 °C. Furthermore, the three most intense precursor ions were selected for acquisition of ms^2^ spectra at collision energies of 40 eV in data-dependent acquisition mode.

After acquisition, the raw data were converted to mzXML format using the Bruker Compass software (Bruker, Bremen, Germany). The resulting mzXML files were processed in MZmine 2 (ver. 2.34). Mass lists were created using the mass detection module with a noise level of 1000 counts for MS^1^ and 40 counts for MS^2^. The chromatogram builder module was used to create peak lists with a minimum retention time of 0.05 min, a minimum peak height of 5000 counts and an *m*/*z* tolerance of 0.01 Da or 10 ppm. The peak lists were deconvoluted using the local minimum search algorithm with the following parameters: chromatographic threshold 0.01%, search minimum in retention time range 0.2 min, minimum relative height 0.1%, minimum absolute height 5000 counts, minimum ratio of peak top/edge 2, peak duration range 0.02–2 min. For MS^2^ scan pairing, the *m*/*z* range was set to 0.05 Da and the retention time range to 0.2 min. Next, the deconvoluted peak lists were aligned using the Join aligner module with an *m*/*z* tolerance of 0.01 Da or 10 ppm and a retention time tolerance of 0.8 min. Weight for *m*/*z* and retention time were both set to 100. The aligned peak list was subsequently filtered to retain only peaks with a paired MS^2^ scan and a retention time between 1.3 and 41.6 min. After filtering, the peak list was deisotoped manually to include only the most intense isotope signal for brominated compounds (^79^Br, ^79^Br^81^Br or ^79^Br_2_^81^Br_2_), followed by gap-filling with the peak finder module, using an intensity tolerance of 30%, an *m*/*z* tolerance of 0.01 Da or 10 ppm, and a retention time tolerance of 0.8 min. Lastly the peak ID’s were reset and the peak list exported for GNPS analysis.

After adjusting the precursor values in the exported feature list to represent the lowest *m*/*z* value isotope for brominated compounds and multiplying all peak areas for samples TIC2016-050C and TIC2016-050AH by 2.5, the files were uploaded to GNPS for molecular networking analysis. A job was submitted, using the molecular feature networking workflow with the following parameters: precursor and fragment ion tolerance 0.02 Da each, minimum number of matching fragments 6, minimum cosine value 0.6, maximum of connected edges per node 6. The spectra were filtered to exclude all signals below 40 counts, within a window of ±17 Da window around the precursor value, and those not at least the sixth most intense within a 50 Da window around their *m*/*z* value. The completed GNPS job can be accessed online at https://gnps.ucsd.edu/ProteoSAFe/status.jsp?task=5b2a35f456a64dd5bf5c1016dd817c3f.

### 4.4. Extraction and Isolation

Frozen sponge material of a chemotype II *T. favus* (TIC2016-050C, 136 g dry weight after extraction) was extracted with MeOH–DCM (1:2, *v*/*v*) to give 227 mg of dry extract. A portion (100 mg) of this extract was separated into 14 fractions on Sephadex LH-20 (H_2_O–MeOH–FA, 80:20:0.05, *v*/*v*/*v*). Fractions 5 and 6 were combined (36 mg) and subjected to semi-prep RP-HPLC purification (H_2_O–MeCN–FA, 80:20:0.05, *v*/*v*/*v*) and yielded compound **1** (3.2 mg). Fraction 7 (12 mg) provided compound **2** (2.1 mg) after semi-prep HPLC purification (H_2_O–MeCN–FA, 95:5:0.05, *v*/*v*/*v*). Fractions 13 and 14 were combined (13 mg) and subsequent semi-prep RP-HPLC purification (H_2_O–MeCN–FA, 80:20:0.05, *v*/*v*/*v*) resulted in the isolation of compound **3** (1.6 mg). A further portion of the organic extract (100 mg) was subjected to a two-step fractionation through RP-SPE (H_2_O–FA, 100:0.05 and MeOH–FA 100:0.05, *v*/*v*/*v*). The second fraction (66.1 mg) was purified through semi-prep RP-HPLC (H_2_O–MeCN–FA, 80:20:0.05, *v*/*v*/*v*) to afford compound **4** (1.8 mg) and compound **5** (1.6 mg).

Frozen sponge material of a chemotype I *T. favus* (TIC2016-050A, 152 g dry weight after extraction) was extracted with MeOH–DCM (1:2, *v*/*v*) to provide 474 mg of dry extract. Part of this extract (100 mg) was fractionated into 12 fractions on Sephadex LH-20 (H_2_O–MeOH–FA, 60:40:0.05, *v*/*v*/*v*). Fraction 5 (8.6 mg) was separated on semi-prep HPLC (H_2_O–MeCN–FA, 80:20:0.05, *v*/*v*/*v*) and resulted in the isolation of compound **6** (2.2 mg) and compound **7** (3.1 mg).

**Makaluvamine Q (1)** green solid; IR (thin film) ν_max_ 3046, 3014, 2916, 1606, 1520, 1421, 1382, 1344 cm^−1^; NMR Data, see Table 1; HRESIMS *m*/*z* 280.0076 ([M + H]^+^, calculated for C_11_H_11_N_3_OBr, 280.0080).

**Makaluvamine A (2)** green solid; ^1^H and ^13^C NMR (600 and 150 MHz, DMSO-*d*_6_) data consistent with published data [6]; HRESIMS *m*/*z* 202.0971 ([M + H]^+^, calculated for C_11_H_12_N_3_O, 202.0975).

**Makaluvamine I (3)** green solid; ^1^H and ^13^C NMR (600 and 150 MHz, DMSO-*d*_6_) data consistent with published data [27]; HRESIMS *m*/*z* 188.0814 ([M + H]^+^, calculated for C_10_H_10_N_3_O, 188.0818).

**Makaluvamine O (4)** grey solid; ^1^H and ^13^C NMR (600 and 150 MHz, DMSO-*d*_6_) data consistent with published data [28]; HRESIMS *m*/*z* 266.9770 ([M + H]^+^, calculated for C_10_H_8_N_2_O_2_Br, 266.9764).

**Makaluvone (5)** grey solid; ^1^H and ^13^C NMR (600 and 150 MHz, DMSO-*d*_6_) data consistent with published data [6]; HRESIMS *m*/*z* 280.9922 ([M + H]^+^, calculated for C_11_H_10_N_2_O_2_^79^Br, 280.9920).

**Tsitsikammamine B (6)** dark red solid; ^1^H and ^13^C NMR (600 and 150 MHz, DMSO-*d*_6_) data consistent with published data [18]; HRESIMS *m*/*z* 318.1226 ([M + H]^+^, calculated for C_19_H_16_N_3_O_2_, 318.1237).

**14-Bromo-7,8-dehydro-3-dihydrodiscorhabdin C (7)** green solid; ^1^H and ^13^C NMR (600 and 150 MHz, DMSO-*d*_6_) data consistent with published data [7]; HRESIMS *m*/*z* 539.8541 ([M + H]^+^, calculated for C_18_H_13_N_3_O_2_^79^Br_3_, 539.8552).

### 4.5. Assays for Bioactivity

Human embryonic kidney (HEK293) and human cervical cancer (HeLa) cells were maintained in DMEM and 10% FBS at 37 °C in an atmosphere that contained 5% CO_2_. HEK293 and HeLa cells were plated at a density of 1 × 10^6^ cells per 1 mL in each well of a 24 well cell culture plate (NEST 702001) and settled overnight. Compound isolates were re-suspended at the concentrations specified in the data tables. The drugs, dissolved in DMSO, were used in combination with the resazurin-based in vitro toxicology assay kit (Catalogue No. R6892,Sigma-Aldrich, St. Louis, MO, USA). Cytotoxicity was evaluated after 48 h.

DNA (200 ng) and 0.8 µg ethidium bromide were diluted to 100 µL in TE buffer (10 mM Tris-HCl pH 8.0; 1 mM EDTA). These samples were incubated at room temperature for 15 min. Compounds of interest were added to the reactions above and incubated for a further 15 min. The samples were excited at 545 nm in a 96 well plate on the SpectraMax M series microplate reader (Molecular Devices, San Jose, CA, USA) and the fluorescence emission measured at 595 nm. The ethidium bromide remaining bound to the DNA was calculated as follows: (TE_Drug_EtBR_DNA) − (TE_Drug)/(TE_EtBr_DNA) * 100. This value was subtracted from 100 to represent the percentage (%) ethidium bromide displaced by the compounds of interest from the DNA.

Human topoisomerase I (Catalogue No. T9069, Sigma-Aldrich, St. Louis, MO, USA) assays were performed in Topoisomerase I assay buffer (10 mM Tris-HCl (pH 7.9), 150 mM NaCl, 0.1% BSA, 0.1 mM spermidine and 5% glycerol) with 1 unit of topoisomerase I and 500 ng DNA. Compounds of interest were added to a final concentration of 1 mM. The reactions were incubated at 37 °C for 1 h, then separated on a 1% agarose gel at 80 V for 1 h. The gel was stained with ethidium bromide before visualization on a UV transilluminator. The fluorescence attributed to circular and nicked DNA was quantified using ImageJ (v1.50i). The inhibition of DNA nicking was calculated as follows: (Fluorescence _Circular DNA_/ Fluorescence _Total DNA_) *100. Two internal controls were included in these assays; (i) DNA with no additional compounds and this assay showed no detectable DNA cleavage and (ii) DNA treated with topoisomerase I and no additional compounds and this assay resulted in the nicking of 100% of the DNA.

## Figures and Tables

**Table 1:** 1D and 2D NMR (600 MHz, MeOD-*d4*) data for makaluvamine Q (**1**).

Atom No.	δ_H_ (J in Hz)	δ_C_ (Type)	HMBC (^1^H-^13^C)	COSY(^1^H-^1^H)
2	7.12, s	132.4 (CH)	N-CH_3_, 2a, 8b	
2a		120.6 (C)		
3	2.92, t (7.7)	19.4 (CH_2_)	2, 2a, 4, 8b	H-4
4	3.88, t (7.7)	44.7 (CH_2_)	2a, 3, 5a	H-3
5a		155.3 (C)		
6		82.1 (C)		
7		156.4 (C)		
8		166.7 (C)		
8a		124.5 (C)		
8b		123.6 (C)		
*N*-CH_3_	3.92, s	36.6 (CH3)	2, 8a	

**Table 2:** Bioassay results of isolated pyrroloiminoquinones and pyrrolo-*ortho*-quinolines.

Compound	Topoisomerase I (% Inhibition of DNA Nicking)	Ethidium Bromide Displacement (%) at 500 µM	Cell Viability (HeLa, % Metabolic Activity at 10 µM)
**1**	27	92.60 ± 2.82	14.7 ± 0.5
**2**	33	84.60 ± 1.05	83.1 ± 6.1
**3**	8	84.48 ± 0.50	73.3 ± 9.2
**4**	41	71.21 ± 1.47	134.9 ± 15.5
**5**	33	70.56 ± 3.67	87.8 ± 12.3
**6**	14	75.18 ± 1.17	101.0 ± 8.4
**7**	25	84.70 ± 2.04	99.5 ± 9.2
Control	86	94.02 ± 0.18	8.2 ± 2.8
	(1 mM Camptothecin) ^1^	(500 µM m-AMSA) ^1^	(0.05 µM Emetine) ^1^

^1^ Concentration of control compound used in the assay.

## Supplementary Materials

The following are available online at http://www.mdpi.com/1660-3397/17/1/60/s1, Table S.1: Collection Metadata; Figure S.2: UV-HPLC scouting; Figure S.3.1 to S.3.3: Spectral matching through GNPS; Figure S.4.1 to S.4.3: Bioassays of isolated pyrroloiminoquinones; Figure S.5.1 to S.5.7: Mass spectrometry data of isolated pyrroloiminoquinones; Figure S.6: Infrared spectrum of makaluvamine Q; Figure S.7.1 to S.7.34: NMR-data of isolated pyrroloiminoquinones.

## References

[1] Antunes E.M., Copp B.R., Davies-Coleman M.T., Samaai T. (2005). Pyrroloiminoquinone and related metabolites from marine sponges. Nat. Prod. Rep..

[2] Hu J., Fan H., Xiong J., Wu S. (2011). Discorhabdins and pyrroloiminoquinone-related alkaloids. Chem. Rev..

[3] Lam C.F.C., Cadelis M.M., Copp B.R. (2017). Exploration of the influence of spiro-dienone moiety on biological activity of cytotoxic marine alkaloid discorhabdin P. Tetrahedron.

[4] Lin S., McCauley E.P., Lorig-Roach N., Tenney K., Naphen C.N., Yang A., Johnson T.A., Hernandez T., Rattan R., Valeriote F.A. (2017). Another look at pyrroloiminoquinone alkaloids-perspectives on their therapeutic potential from known structures and semisynthetic analogues. Mar. Drugs.

[5] Gunasekera S.P., McCarthy P.J., Longley R.E., Pomponi S.A., Wright A.E., Lobkovsky E., Clardy J.J. (1999). Discorhabdin P, a new enzyme inhibitor from deep-water Caribbean sponge of the genus *Batzella*. J. Nat. Prod..

[6] Radisky D.C., Radisky E.S., Barrows L.R., Copp B.R., Kramer R.A., Ireland C.M. (1993). Novel cytotoxic topoisomerase II inhibiting pyrroloiminoquinones from Fijian sponges of the genus *Zyzzya*. J. Am. Chem. Soc..

[7] Antunes E.M., Beukes D.R., Kelly M., Samaai T., Barrows L.R., Marshall K.M., Sincich C., Davies-Coleman M.T. (2004). Cytotoxic pyrroloiminoquinones from four new species of South African latrunculid sponges. J. Nat. Prod..

[8] Dijoux M., Schnabel P.C., Hallock Y.F., Boswell J.L., Johnson T.R., Wilson J.A., Ireland C.M., van Soest R., Boyd M.R., Barrows L.R. (2005). Antitumor activity and distribution of pyrroloiminoquinones in the sponge genus *Zyzzya*. Bioorg. Med. Chem..

[9] Perry N.B., Blunt J.W., Munro M.H.G. (1988). Cytotoxic pigments from New Zealand sponges of the genus *Latrunculia*: Discorhabdins A, B and C. Tetrahedron.

[10] Ford J., Capon R.J. (2000). Discorhabdin R: A new antibacterial pyrroloiminoquinone from two latrunculid marine sponges, *Latrunculia* sp. and *Negombata* sp.. J. Nat. Prod..

[11] Davis R.A., Buchanan M.S., Duffy S., Avery V.M., Charman S.A., Charman W.N., White K.L., Shackleford D.M., Edstein M.D., Andrews K.T. (2012). Antimalarial activity of pyrroloiminoquinones from the Australian marine sponge *Zyzzya* sp.. J. Med. Chem..

[12] Alonso E., Alvariño R., Leirós M., Tabudravu J.N., Feussner K., Dam M.A., Rateb M.E., Jaspars M., Botana L.M. (2016). Evaluation of the antioxidant activity of the marine pyrroloiminoquinone makaluvamines. Mar. Drugs.

[13] Carney J.R., Scheuer P.J., Kelly-Borges M. (1993). Makaluvamine G, a cytotoxic pigment from an Indonesian sponge *Histodermella* sp.. Tetrahedron.

[14] Botić T., Defant A., Zanini P., Žužek M.C., Frangež R., Janussen D., Kersken D., Knez Ž., Mancini I., Sepčić K. (2017). Discorhabdin alkaloids from Antarctic *Latrunculia* spp. sponges as a new class of cholinesterase inhibitors. Eur. J. Med. Chem..

[15] Harris E.M., Strope J.D., Beedie S.L., Huang P.A., Goey A.K.L., Cook K.M., Schofield C.J., Chau C.H., Cadelis M.M., Copp B.R. (2018). Preclinical evaluation of discorhabdins in antiangiogenic and antitumor models. Mar. Drugs.

[16] Wang W., Nijampatnam B., Velu S.E., Zhang R. (2016). Discovery and development of synthetic tricyclic pyrroloquinone (TPQ) alkaloid analogs for human cancer therapy. Front. Chem. Sci. Eng..

[17] Goey A.K.L., Chau C.H., Sissung T.M., Cook K.M., Venzon D.J., Castro A., Ransom T.R., Henrich C.J., McKee T.C., McMahon J.B. (2016). Screening and biological effects of marine pyrroloiminoquinone alkaloids: Potential inhibitors of the HIF-1α/p300 interaction. J. Nat. Prod..

[18] Hooper G.J., Davies-Coleman M.T., Kelly-Borges M., Coetzee P.S. (1996). New alkaloids from a South African latrunculid sponge. Tetrahedron Lett..

[19] Li F., Janussen D., Pfeifer C., Pérez-Victoria I., Tasdemir D. (2018). Targeted isolation of tsitsikammamines from the Antarctic deep-sea sponge *Latrunculia biformis* by molecular networking and anticancer activity. Mar. Drugs.

[20] Yang A., Baker B.J., Grimwade J., Leonard A., McClintock J.B. (1995). Discorhabdin alkaloids from the Antarctic sponge *Latrunculia* apicalis. J. Nat. Prod..

[21] Perry N.B., Blunt J.W., McCombs J.D., Munro M.H.G. (1986). Discorhabdin C, a highly cytotoxic pigment from a sponge of the genus *Latrunculia*. J. Org. Chem..

[22] Jeon J., Na Z., Jung M., Lee H., Sim C.J., Nahm K., Oh K.-B., Shin J. (2010). Discorhabdins from the Korean marine sponge *Sceptrella* sp.. J. Nat. Prod..

[23] Lill R.E., Major D.A., Blunt J.W., Munro M.H.G., Battershill C.N., McLean M.G., Baxter R.L. (1995). Studies on the biosynthesis of discorhabdin B in the New Zealand sponge *Latrunculia* sp. B. J. Nat. Prod..

[24] Urban S., Hickford S.J.H., Blunt J.W., Munro M.H.G. (2000). Bioactive marine alkaloids. Curr. Org. Chem..

[25] Tatsuta K., Imamura K., Itoh S., Kasai S. (2004). The first total synthesis of lymphostin. Tetrahedron Let..

[26] Matcher G.F., Waterworth S.C., Walmsley T.A., Matsatsa T., Parker-Nance S., Davies-Coleman M.T., Dorrington R.A. (2016). Keeping it in the family: Coevolution of latrunculid sponges and their dominant bacterial symbionts. MicrobiologyOpen.

[27] Schmidt E.W., Harper M.K., Faulkner D.J. (1995). Makaluvamines H-M and damirone C from the Pohnpeian sponge *Zyzzya fuliginosa*. J. Nat. Prod..

[28] Hu J., Schetz J.A., Kelly M., Peng J., Ang K.K.H., Flotow H., Yan Leong C., Bee Ng S., Buss A.D., Wilkins S.P. (2002). New antiinfective and human 5-HT2 receptor binding natural and semisynthetic compounds from the Jamaican sponge *Smenospongia aurea*. J. Nat. Prod..

[29] Venables D.A., Concepciόn G.P., Matsumoto S.S., Barrows L.R., Ireland C.M. (1997). Makaluvamine N: A new pyrroloiminoquinone from *Zyzzya fuliginosa*. J. Nat. Prod..

[30] Pluskal T., Castillo A., Villar-Briones M., Orešič M. (2010). MZmine2: Modular framework for processing, visualizing and analyzing mass spectrometry-based molecular profile data. BMC Bioinf..

[31] Wang M., Carver J.J., Phelan V.V., Sanchez L.M., Garg N., Peng Y., Nguyen D.D., Watrous J., Kapono C.A., Luzzatto-Knaan T. (2016). Sharing and community curation of mass spectrometry data with Global Natural Products Social Molecular Networking. Nat. Biotechnol..

[32] Sun H.H., Sakemi S., Burres N., McCarthy P. (1990). Isobatzelline A, B, C and D. Cytotoxic and antifungal pyrroloquinoline alkaloids from the marine sponge *Batzella* sp.. J. Org. Chem..

[33] Sakemi S., Sun H.H., Jefford C.W., Bernardinelli G. (1989). Batzellines A, B and C. Novel pyrroloquinoline alkaloids from the sponge *Batzella* sp.. Tetrahedron Lett..

[34] Tasdemir D., Mangalindan G.C., Concepciόn G.P., Harper M.K., Ireland C.M. (2001). 3,7-dimethylguanine, a new purine from a Philippine sponge *Zyzzya fuliginosa*. Chem. Pharm. Bull..

[35] Grkovic T., Copp B.R. (2009). New natural products in the discorhabdin A- and B-series from New Zealand-sourced *Latrunculia* spp. sponges. Tetrahedron.

[36] Zhu H., Xu G., Zhang K., Kong X., Han R., Zhou J., Ni Y. (2016). Crystal structure of tyrosine decarboxylase and identification of key residues involved in conformational swing and substrate binding. Sci. Rep..

[37] Yoo H., Widhalm J.R., Qian Y., Maeda H., Cooper B.R., Jannasch A.S., Gonda I., Lewinsohn E., Rhodes D., Dudareva N. (2013). An alternative pathway contributes to phenylalanine biosynthesis in plants via a cytosolic tyrosine:phenylpyruvate aminotransferase. Nat. Commun..

[38] Wiens M., Koziol C., Batel R., Müller W.E.G. (1998). Phenylalanine hydroxylase from the sponge Geodia cydonium: Implication for allorecognition and evolution of aromatic amino acid hydroxylases. Dev. Comp. Immunol..

[39] Nichol C.A., Lun Lee C., Edelstein M.P., Chao Y.J., Duch D.S. (1983). Biosynthesis of tetrahydrobiopterin by de novo and salvage pathways in adrenal medulla extracts, mammalian cell cultures, and rat brain in vivo. Proc. Natl. Acad. Sci. USA.

[40] Weber G., Nakamura H., Natsumeda Y., Szekeres T., Nagai M. (1992). Regulation of GTP biosynthesis. Adv. Enzyme Regul..

[41] Ishibashi M., Iwasaki T., Imai S., Sakamoto S., Yamaguchi K., Ito A. (2001). Laboratory culture of the myxomycetes: Formation of fruiting bodies of *Didymium bahiense* and its plasmodial production of makaluvamine A. J. Nat. Prod..

[42] Miyanaga A., Janso J.E., McDonald L., He M., Liu H., Barbieri L., Eustáquio A.S., Fielding E.N., Carter G.T., Jensen P.R. (2011). Discovery and assembly-line biosynthesis of the lymphostin pyrroloquinoline alkaloid family of mTOR inhibitors in *Salinispora* bacteria. J. Am. Chem. Soc..

[43] Waterworth S.C., Jiwaji M., Kalinski J.-C.J., Parker-Nance S., Dorrington R.A. (2017). A place to call home: An analysis of the bacterial communities in two *Tethya rubra* Samaai and Gibbons 2005 populations in Algoa Bay, South Africa. Mar. Drugs.

[44] Schloss P.D., Westcott S.L., Ryabin T., Hall J.R., Hartmann M., Hollister E.B., Weber C.F. (2009). Introducing mothur: Open-source, platform-independent, community-supported software for describing and comparing microbial communities. Appl. Env. Microbiol..

[45] Rognes T., Flouri T., Nichols B., Quince C., Mahé F. (2016). VSEARCH: A versatile open source tool for metagenomics. PeerJ..

