# Supplementary material for: Molecular Networking Reveals Two Distinct Chemotypes in Pyrroloiminoquinone-Producing Tsitsikamma favus Sponges
Source: Mar Drugs. 2019 Jan 16;17(1):60. doi: 10.3390/md17010060 (PMC6356464; doi:10.3390/md17010060)
Supplement: Supplementary file 1 [file marinedrugs-17-00060-s001.pdf]

Table of Contents

Supplementary information 1 Sample Collection 3

S.1 Collection Metadata for sponges collected at Evan's Peak, Algoa Bay 3

Supplementary information 2 UV-HPLC scouting of *T.favus* specimens 3

S.2 UV-HPLC analysis (60:40:0.05, H_2O:MeOH:FA; 1.1 mL/min; 244 nm, 0.46x250mm XBridge RP-shield) of methanol extracts of frozen *T. favus* specimens collected at Evan's Peak, Algoa Bay in September 2015 and March 2016. 7

Supplementary information 3 Spectral matching using GNPS 8

S.3.1 Mirror plot of ms^2 spectra for the 269.1 Da precursor identified as inosine (black) and a database spectrum for hypoxanthine (green), matching with a cosine value of 0.93. 8

S.3.2 Mirror plot of ms^2 spectra for the 284.1 Da precursor identified as guanosine (black) and a database spectrum for guanine (green), matching with a cosine value of 0.85. 8

S.3.3 Mirror plot of ms^2 spectra for the 166.1 Da precursor identified as phenylalanine (black) and a database spectrum for phenylalanine (green), matching with a cosine value of 0.92. 8

Supplementary information 4 Bioassays for isolated pyrroloiminoquinones 9

S.4.1 Antimetabolic activity vs HEK293 for compounds 1-7 at 50, 5 and 0.5 μM concentrations. 9

S.4.2 Ethidium bromide displacement for compounds 1-7 at 50, 5 and 0.5 μM concentrations. 9

S.4.3 SDS-PAGE gel showing topoisomerase I activities of compounds 1-7. Constricted chromosomes are decatenated through topoisomerase I activity, causing steric hindrance to delay the progression of DNA through the gel. Hence the presence of an uncut band (see camptothecin) indicates topoisomerase I inhibition. 10

Supplementary information 5 Mass spectrometry data for pyrroloiminoquinone isolates 10

S.5.1 Mass spectra (A ms^1; B ms^2) for makaluvamine Q (1). 10

S.5.2 Mass spectra (A ms^1; B ms^2) for makaluvamine A (2). 11

S.5.3 Mass spectra (A ms^1; B ms^2) for makaluvamine I (3). 11

S.5.4 Mass spectra (A ms^1; B ms^2) for makaluvamine O (4). 12

S.5.5 Mass spectra (A ms^1; B ms^2) for makaluvone (5). 12

S.5.6 Mass spectra (A ms^1; B ms^2) for tsitsikammamine B (6). 13

S.5.7 Mass spectra (A ms^1; B ms^2) for 14-bromo-3-dihydro-7,8-dehydrodiscorhabdin C (7). 13

Supplementary information 6 Infrared spectroscopy 14

S.6.1 IR spectrum for makaluvamine Q (1). 14

Supplementary information 7 NMR data for pyrroloiminoquinone isolates 14

S.7.1 ^1H NMR spectrum for makaluvamine Q (1) in MeOD-d_4. 14

S.7.2 ^{13}C NMR spectrum for makaluvamine Q (1) in MeOD-d_4. 15

S.7.3 DEPT-135 spectrum for makaluvamine Q (1) in MeOD-d_4. 15

S.7.4 COSY spectrum of makaluvamine Q (1) in MeOD-d_4 and chemical structure highlighting key COSY (^1H-^1H) correlations (arrows). 16

S.7.5 HSQC spectrum for makaluvamine Q (1) in MeOD-d_4. 16

S.7.6 HMBC spectrum for makaluvamine Q (1) in MeOD-d_4 and chemical structure highlighting key HMBC (^{1}H-^{13}C) correlations.(arrows). 17
S.7.7 ^{1}H NMR spectrum for makaluvamine A (2) in MeOD-d_4. 17
S.7.8 ^{13}C NMR spectrum for makaluvamine A (2) in MeOD-d_4. 18
S.7.9 DEPT-135 spectrum for makaluvamine A (2) in MeOD-d_4. 18
S.7.10 COSY spectrum for makaluvamine A (2) in MeOD-d_4. 19
S.7.11 HSQC spectrum for makaluvamine A (2) in MeOD-d_4. 19
S.7.12 HMBC spectrum for makaluvamine A (2) in MeOD-d_4. 20
S.7.13 ^{1}H NMR spectrum for makaluvamine I (3) in MeOD-d_4. 20
S.7.14 ^{13}C NMR spectrum for makaluvamine I (3) in MeOD-d_4. 21
S.7.15 DEPT-135 spectrum for makaluvamine I (3) in MeOD-d_4. 21
S.7.16 COSY spectrum for makaluvamine I (3) in MeOD-d_4. 22
S.7.17 HSQC spectrum for makaluvamine I (3) in MeOD-d_4. 22
S.7.18 HMBC spectrum for makaluvamine I (3) in MeOD-d_4. 23
S.7.19 ^{1}H NMR spectrum for makaluvamine O (4) in MeOD-d_4. 23
S.7.20 ^{13}C spectrum for makaluvamine O (4) in MeOD-d_4. 24
S.7.21 HMBC spectrum for makaluvamine E (4) in MeOD-d_4. 24
S.7.22 HSQC spectrum for makaluvamine E (4) in MeOD-d_4. 25
S.7.23 ^{1}H NMR spectrum for makaluvone (5) in DMSO-d_6. 25
S.7.24 ^{1}H NMR spectrum for tsitsikammamine B (6) in MeOD-d_4. 26
S.7.25 ^{1}H NMR spectrum for tsitsikammamine B (6) in DMSO-d_6. 26
S.7.26 ^{13}C NMR spectrum for tsitsikammamine B (6) in DMSO-d_6. 27
S.7.27 DEPT-135 spectrum for tsitsikammamine B (6) in DMSO-d_6. 27
S.7.28 COSY spectrum for tsitsikammamine B (6) in DMSO-d_6. 28
S.7.29 HSQC spectrum for tsitsikammamine B (6) in DMSO-d_6. 28
S.7.30 ^{1}H NMR spectrum for 14-bromo-7,8-dehydro-3-dihydrodiscorhabdin C (7) in MeOD-d_4. 29
S.7.31 ^{13}C NMR spectrum for 14-bromo-7,8-dehydro-3-dihydrodiscorhabdin C (7) in MeOD-d_4. 29
S.7.32 COSY spectrum for 14-bromo-7,8-dehydro-3-dihydrodiscorhabdin C (7) in MeOD-d_4. 30
S.7.33 HMBC spectrum for 14-bromo-7,8-dehydro-3-dihydrodiscorhabdin C (7) in MeOD-d_4. 30
S.7.34 HSQC spectrum for 14-bromo-7,8-dehydro-3-dihydrodiscorhabdin C (7) in MeOD-d_4. 31

Supplementary information 1 Sample Collection

S.1 Collection Metadata for sponges collected at Evan's Peak, Algoa Bay

Specimen ID	Collection date	Depth	28S rRNA sequence	Chemotype	Reference
TIC2012-057	Dec 2012	30 m	KU535626l	ND	Matcher et al., 2017
TIC2014-001	Aug 2014	30 m	KU535627l	ND	Matcher et al., 2017
TIC2015-027[1]	Sep 2015	30 m	MG640038.1	II	This study
TIC20160-50A[2]	Mar 2016	20 m	MG203892.1	I	This study
TIC2016-050B[2]	Mar 2016	20 m	ND	I	This study
TIC2016-050C[2]	Mar 2016	20 m	ND	II	This study
TIC2016-050D[2]	Mar 2016	20 m	MG640038.1	I	This study
TIC2016-050AH[2]	Mar 2016	20 m	MG640038.1	II	This study
TIC2016-050AW[2]	Mar 2016	20 m	ND	I	This study

[1]Collection permit: RES2015/16 issued by the South African National Department of Environmental Affairs

[2] Collection permit: RES2016/11 issued by the South African National Department of Environmental Affairs

Supplementary information 2 UV-HPLC scouting of *T.favus* specimens

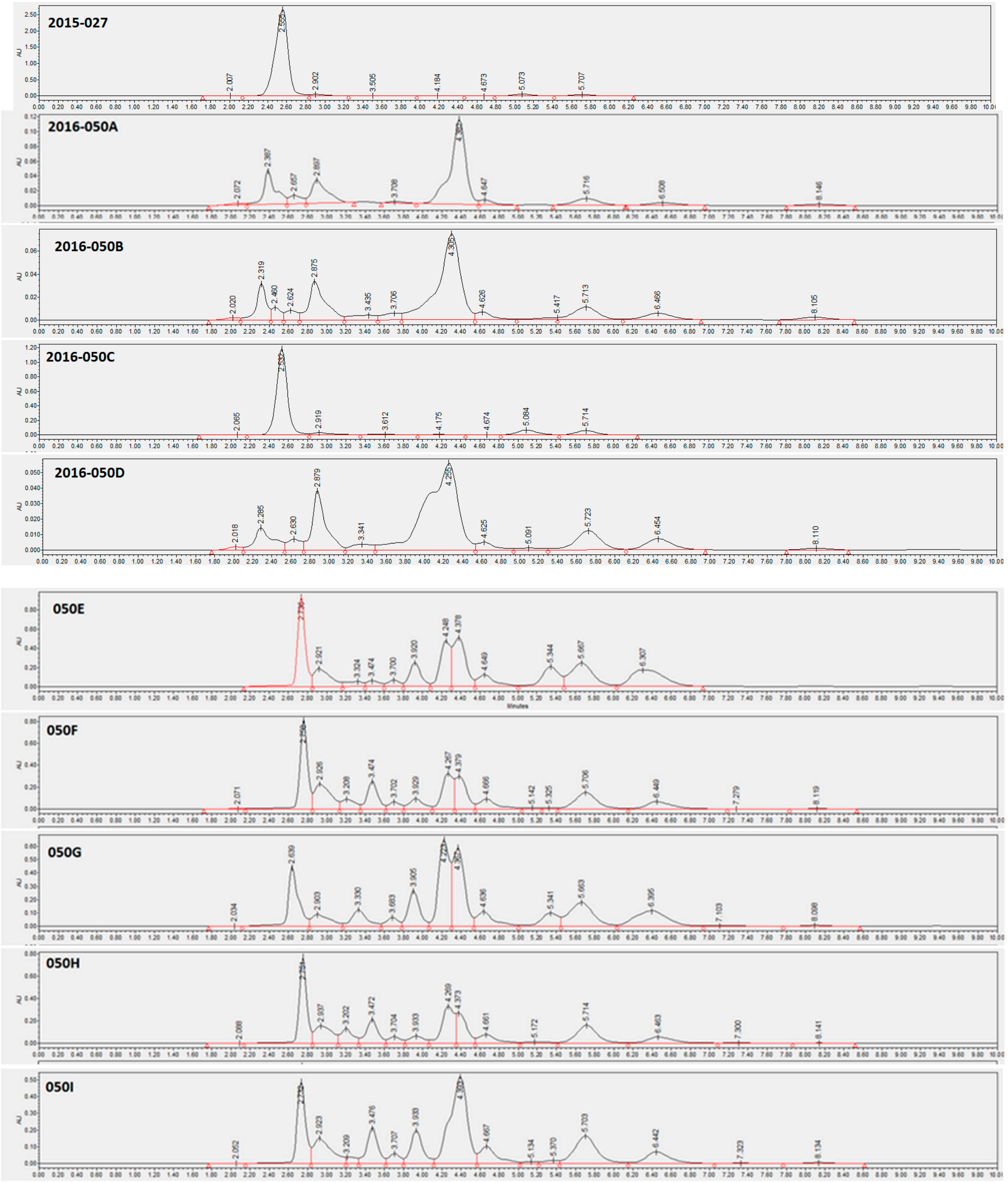

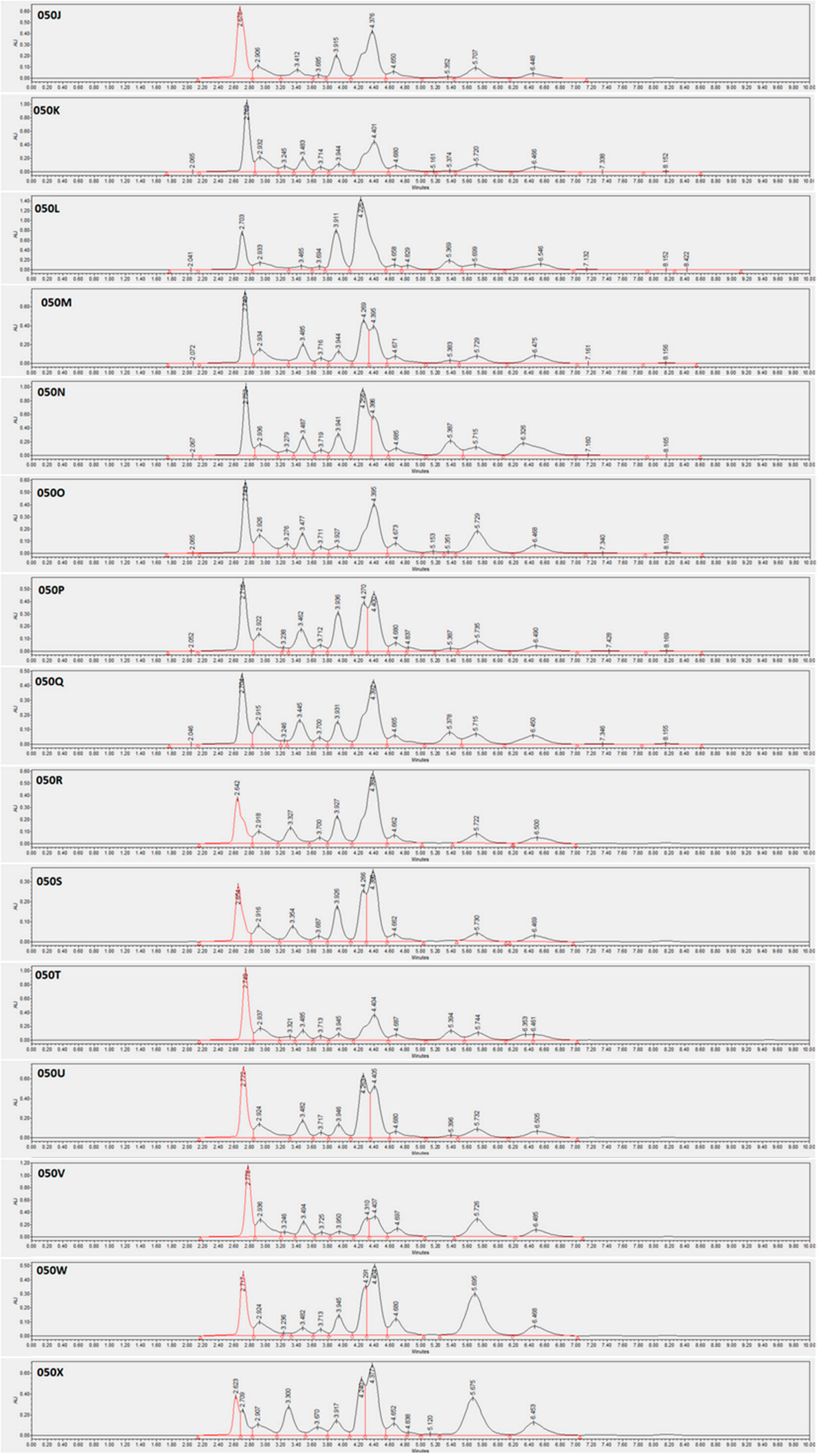
050J
050K
050L
050M
050N
050O
050P
050Q
050R
050S
050T
050U
050V
050W
050X

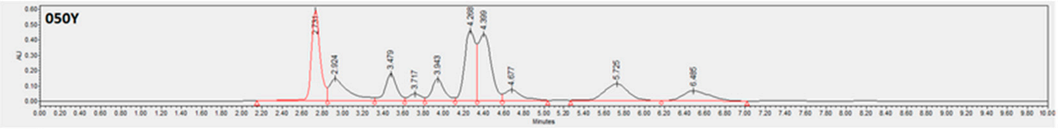
050Y
AU
Minutes

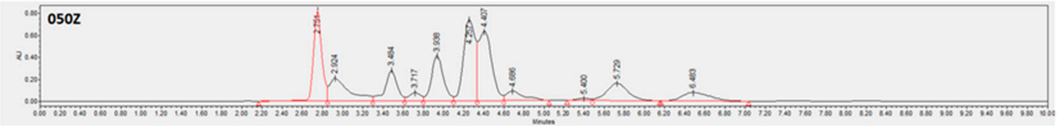
050Z
AU
Minutes

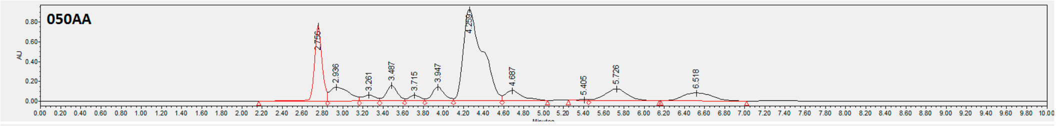
050AA
AU
Minutes

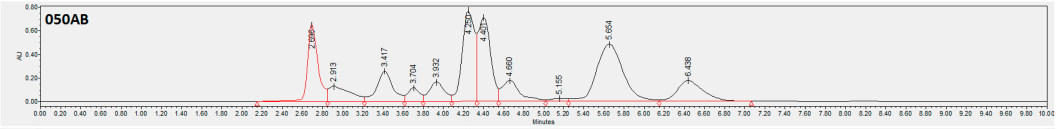
050AB
AU
Minutes

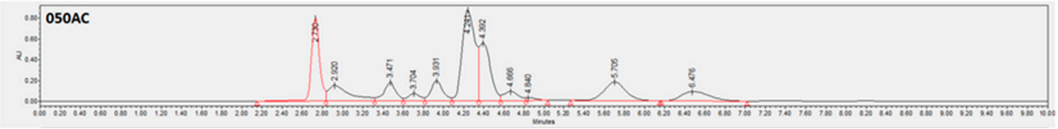
050AC
AU
Minutes

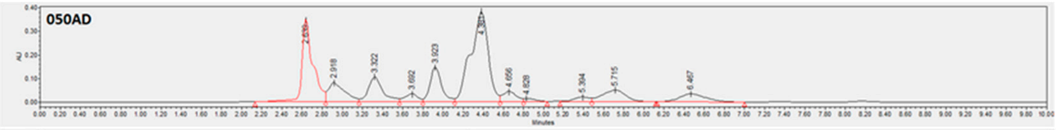
050AD
AU
Minutes

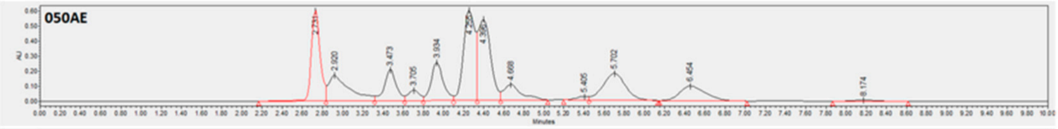
050AE
AU
Minutes

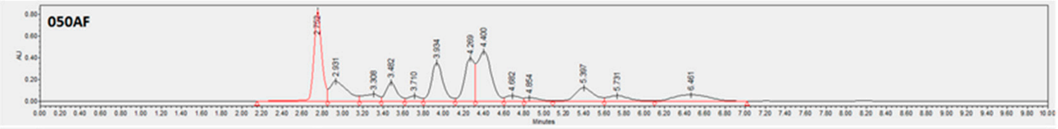
050AF
AU
Minutes

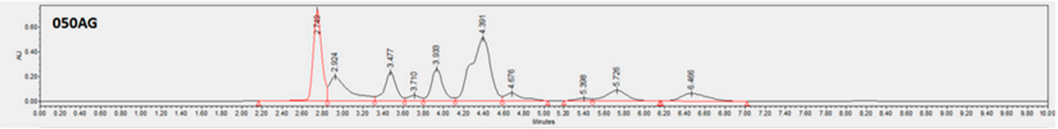
050AG
AU
Minutes

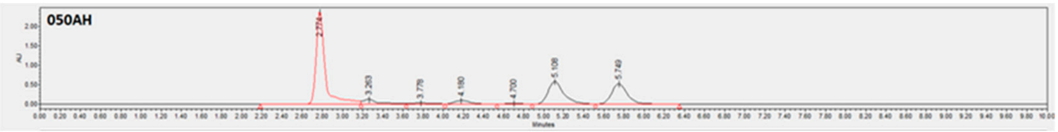
050AH
AU
Minutes

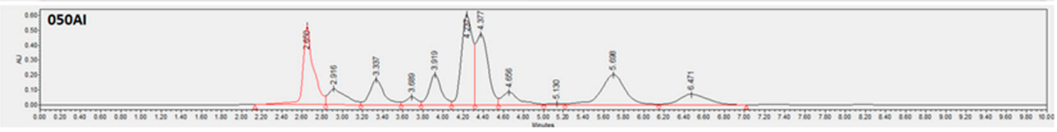
050AI
AU
Minutes

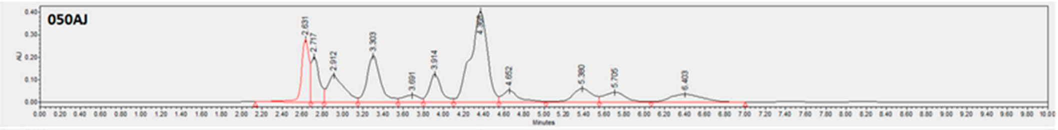
050AJ
AU
Minutes

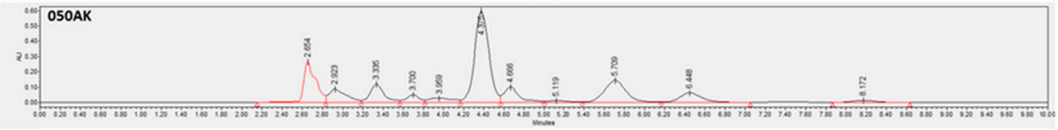
050AK
AU
Minutes

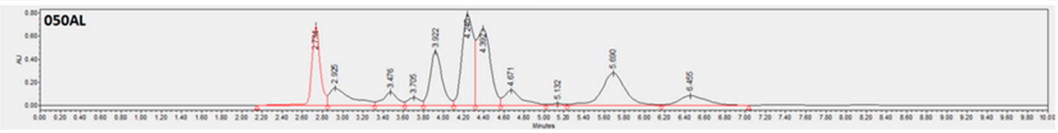
050AL
AU
Minutes

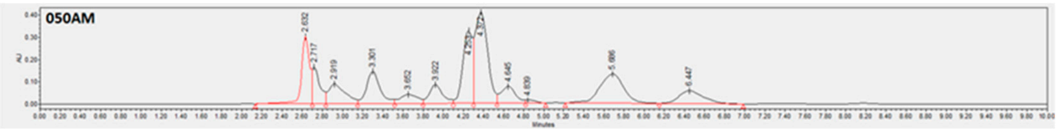
050AM
AU
Minutes

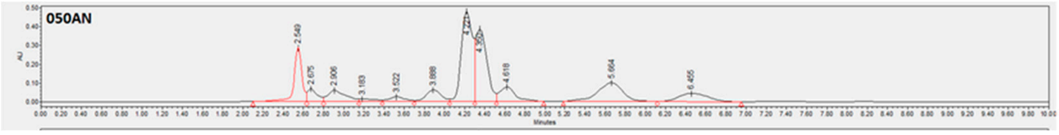
050AN
AU
Minutes

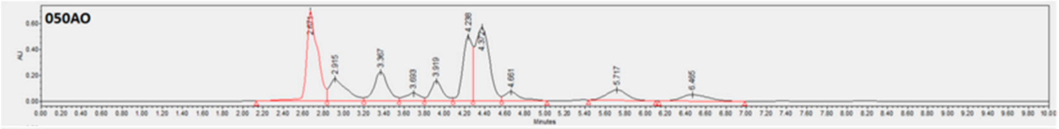
050AO
AU
Minutes

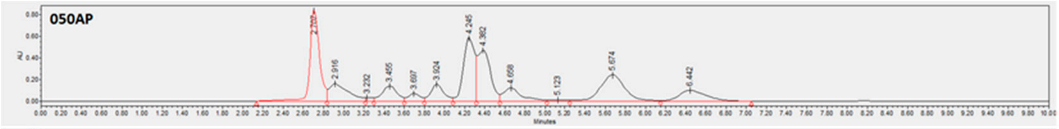
050AP
AU
Minutes

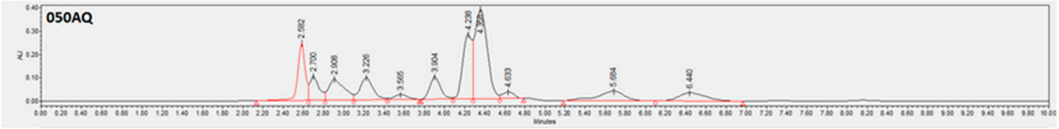
050AQ
AU
Minutes

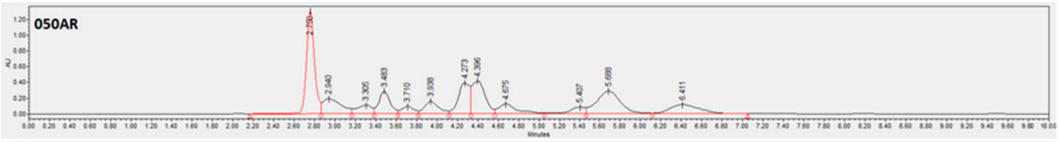
050AR
AU
Minutes

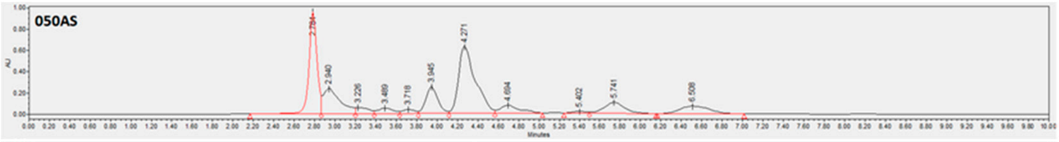
050AS
AU
Minutes

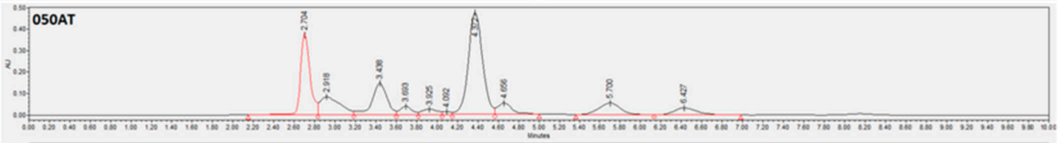
050AT
AU
Minutes

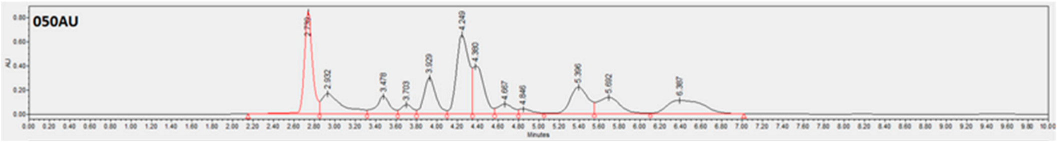
050AU
AU
Minutes

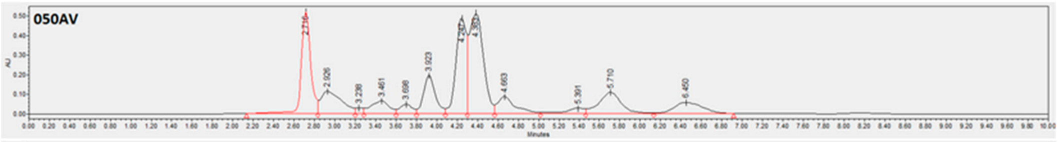
050AV
AU
Minutes

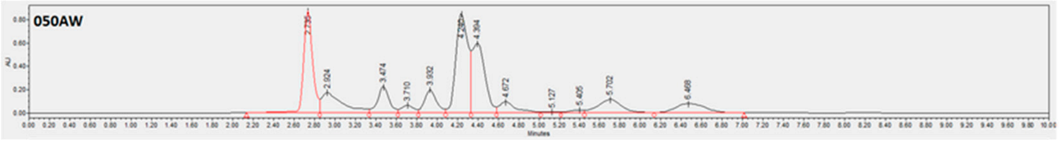
050AW
AU
Minutes

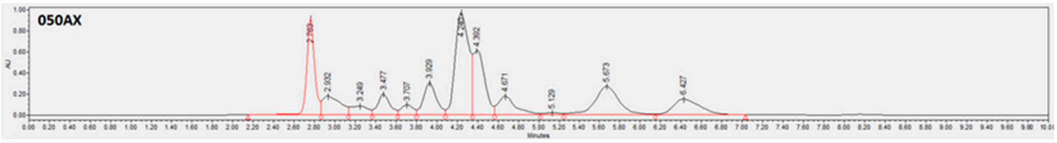
050AX
AU
Minutes

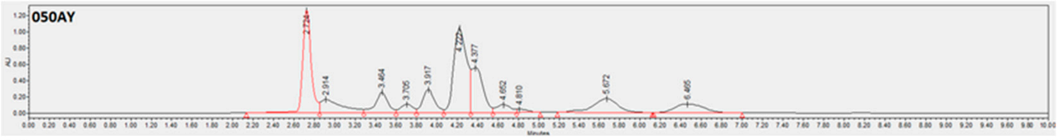
050AY
AU
Minutes

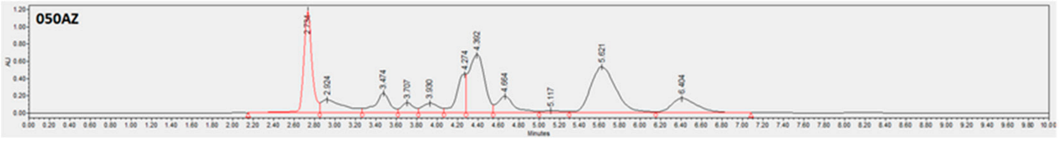
050AZ
AU
Minutes

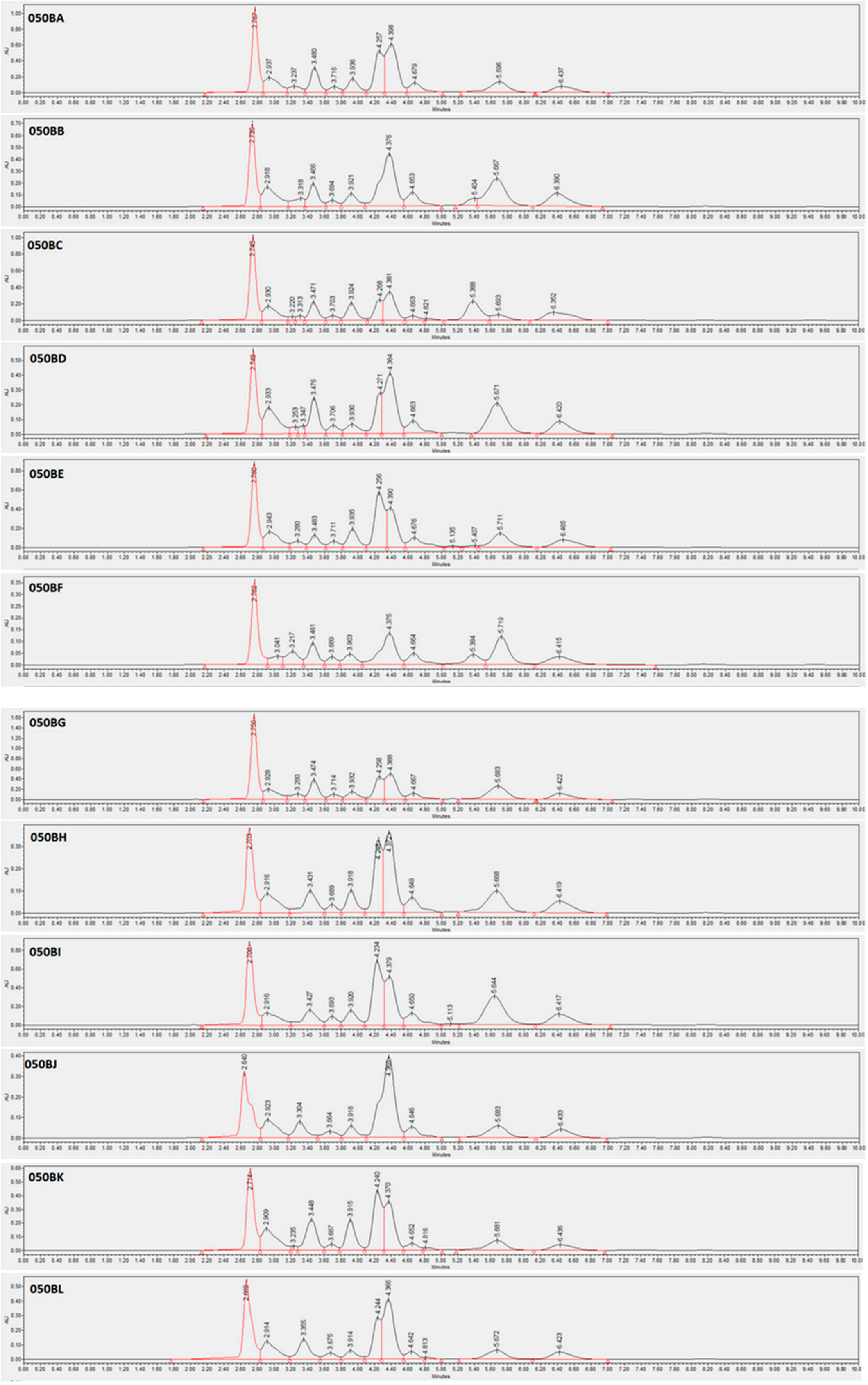

S.2 UV-HPLC analysis (60:40:0.05, H_2O:MeOH:FA; 1.1 mL/min; 244 nm, 0.46x250mm XBridge RP-shield) of methanol extracts of frozen *T. favus* specimens collected at Evan's Peak, Algoa Bay in September 2015 and March 2016.

Supplementary information 3 Spectral matching using GNPS

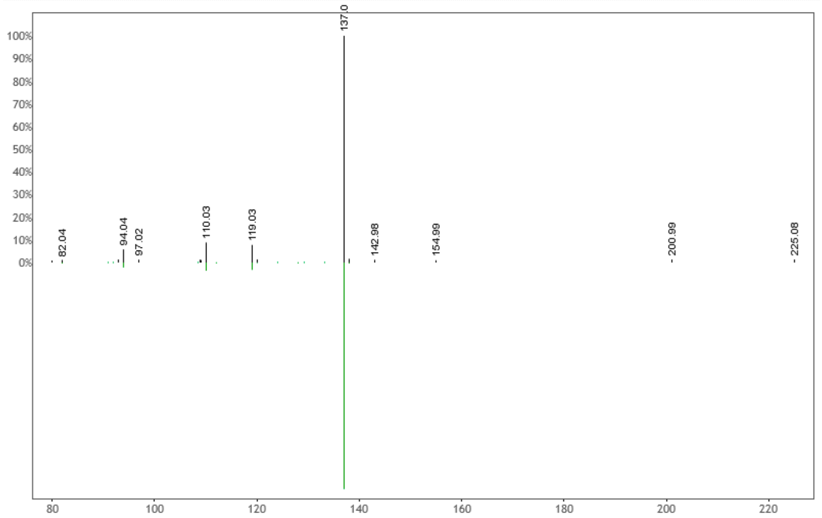

S.3.1 Mirror plot of ms² spectra for the 269.1 Da precursor identified as inosine (black) and a database spectrum for hypoxanthine (green), matching with a cosine value of 0.93.

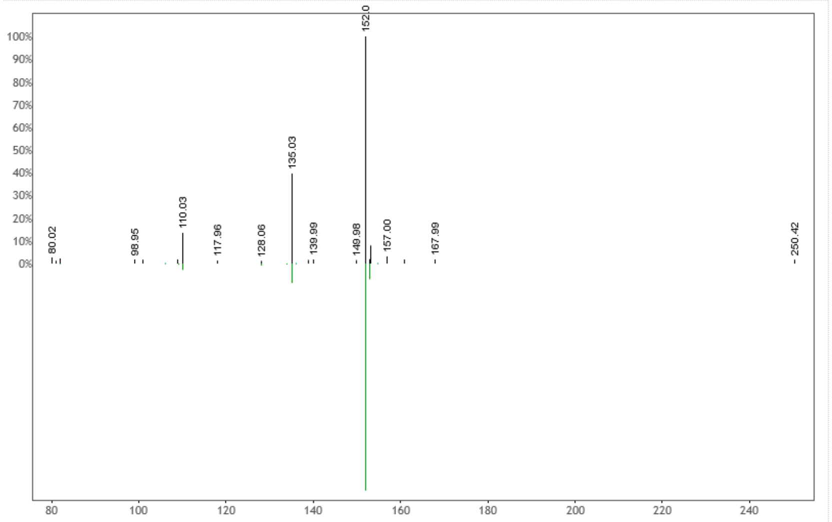

S.3.2 Mirror plot of ms² spectra for the 284.1 Da precursor identified as guanosine (black) and a database spectrum for guanine (green), matching with a cosine value of 0.85.

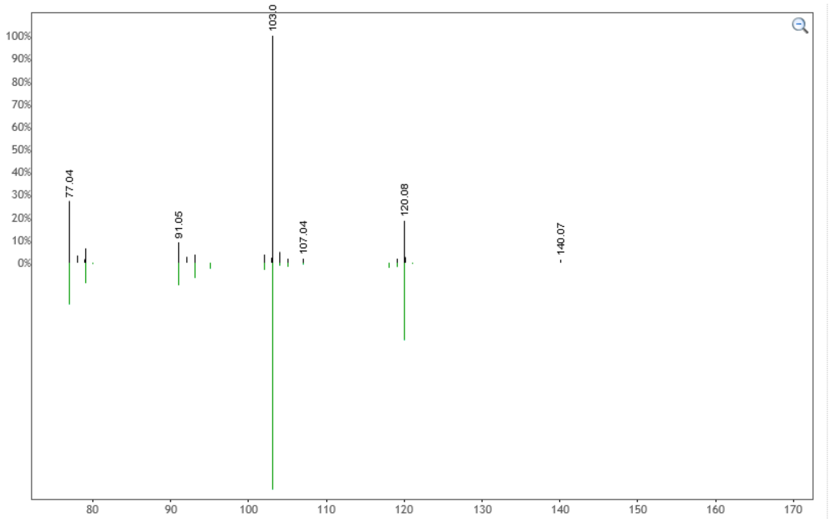

S.3.3 Mirror plot of ms² spectra for the 166.1 Da precursor identified as phenylalanine (black) and a database spectrum for phenylalanine (green), matching with a cosine value of 0.92.

Supplementary information 4 Bioassays for isolated pyrroloiminoquinones

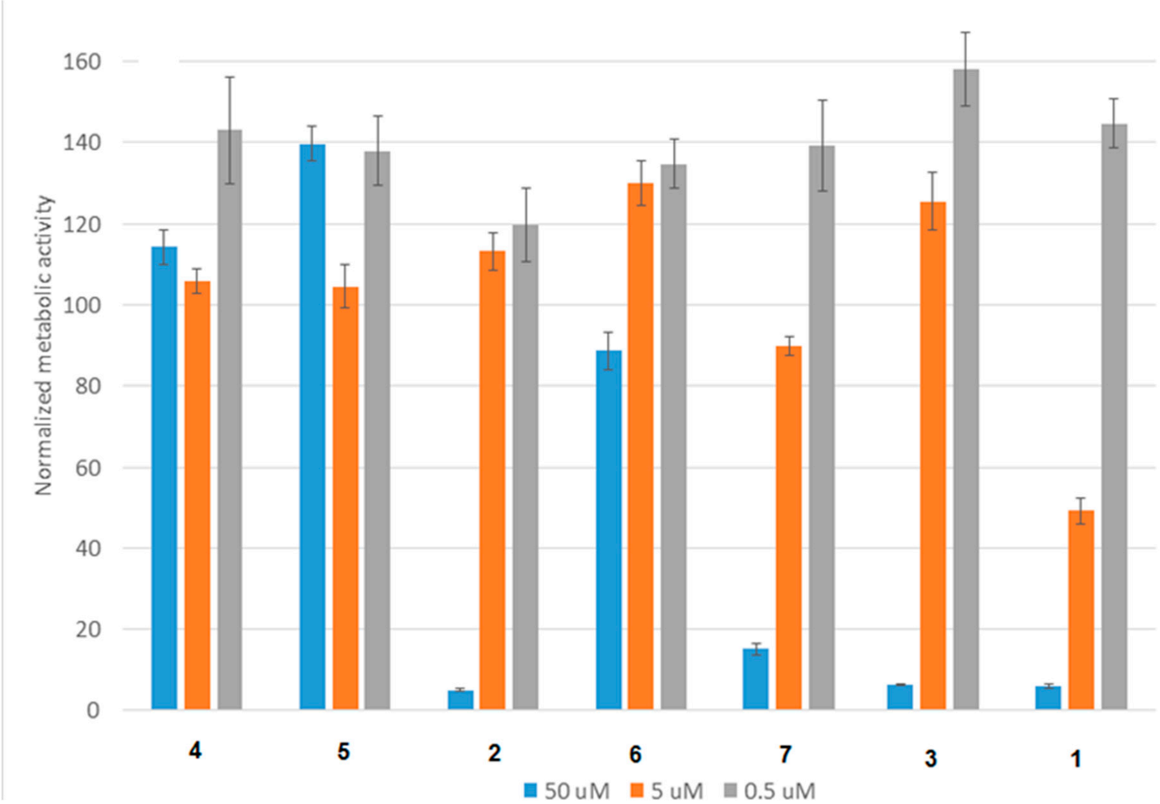

S.4.1 Antimetabolic activity vs HEK293 for compounds **1-7** at 50, 5 and 0.5 µM concentrations.

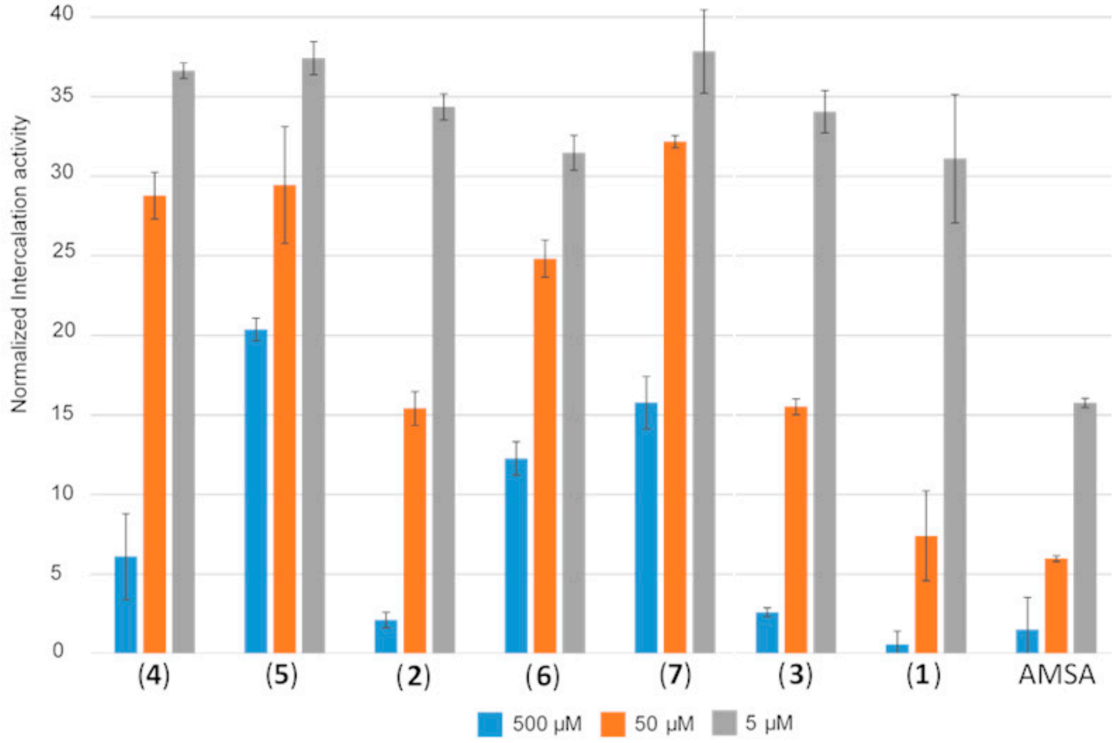

S.4.2 Ethidium bromide displacement for compounds **1-7** at 50, 5 and 0.5 µM concentrations.

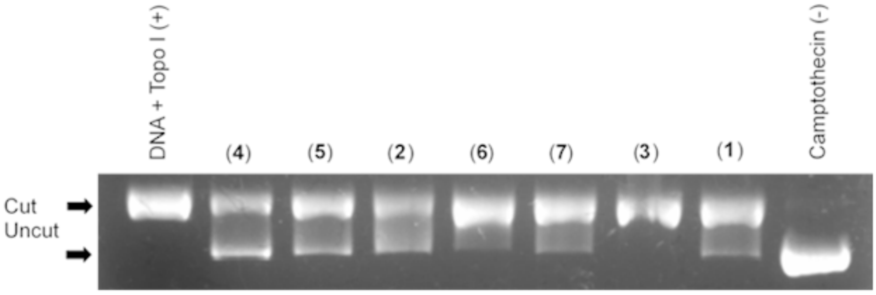

S.4.3 SDS-PAGE gel showing topoisomerase I activities of compounds **1**-**7**. Constricted chromosomes are decatenated through topoisomerase I activity, causing steric hindrance to delay the progression of DNA through the gel. Hence the presence of an uncut band (see camptothecin) indicates topoisomerase I inhibition.

Supplementary information 5 Mass spectrometry data for pyrroloiminoquinone isolates

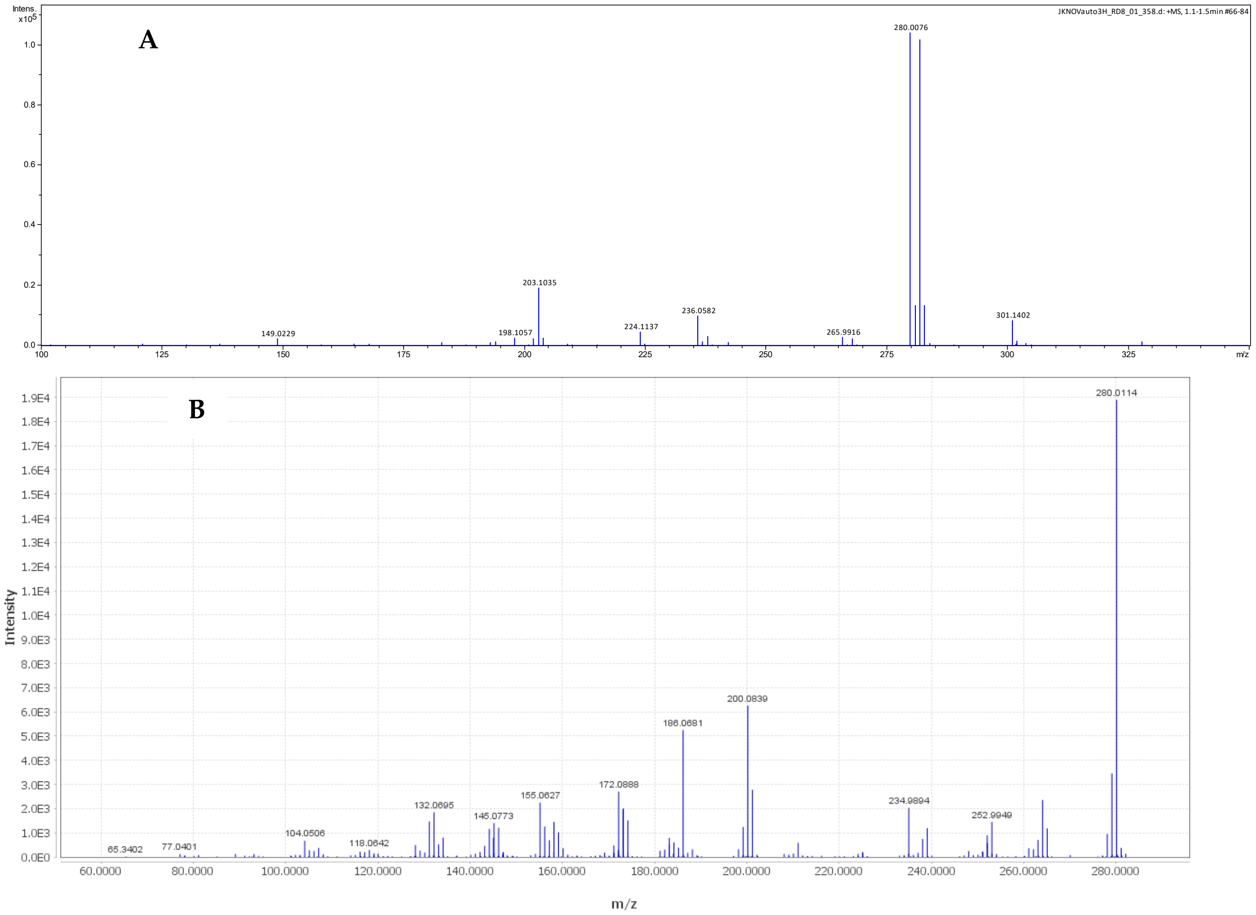

S.5.1 Mass spectra (**A** ms[1]; **B** ms[2]) for makaluvamine Q (**1**).

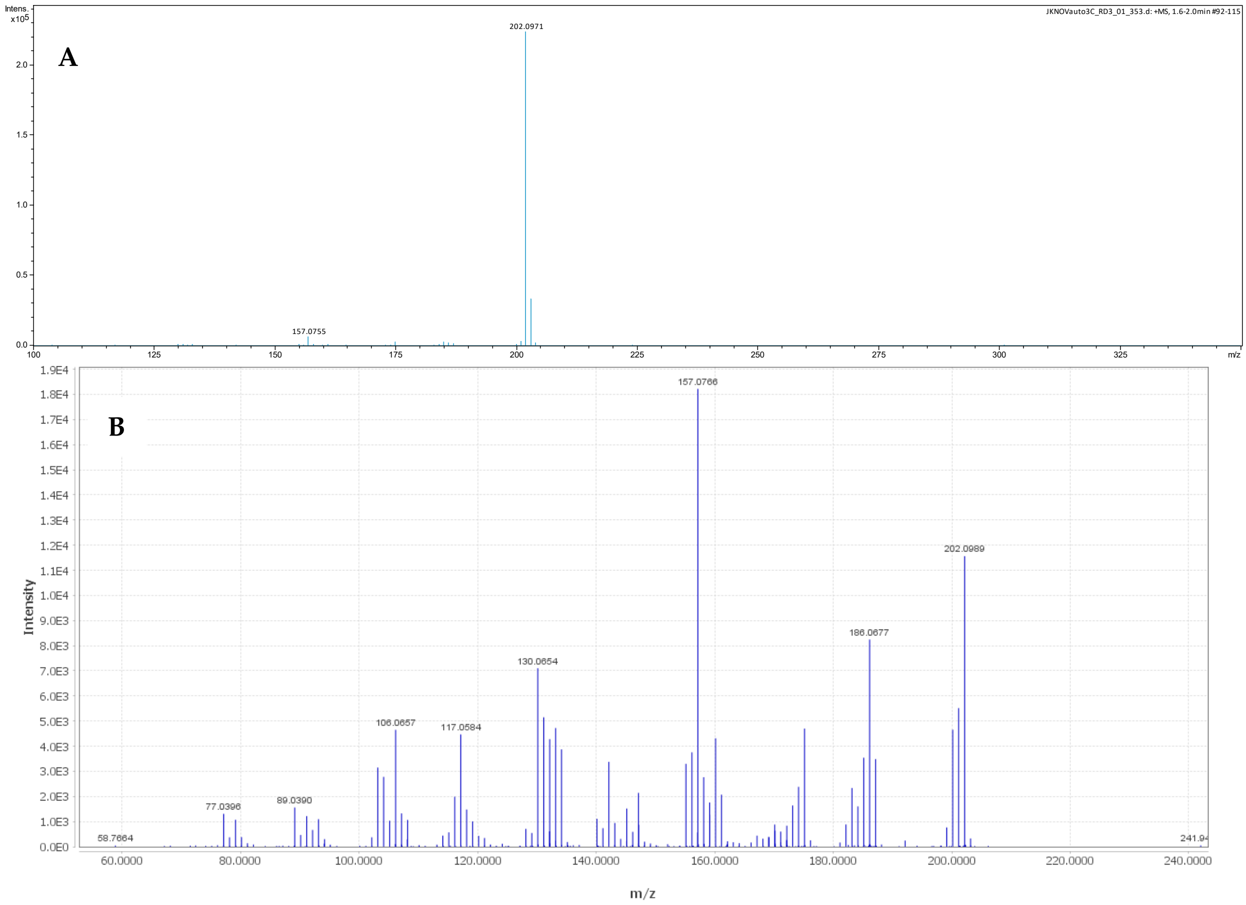

S.5.2 Mass spectra (**A** ms[1]; **B** ms[2]) for makaluvamine A (**2**).

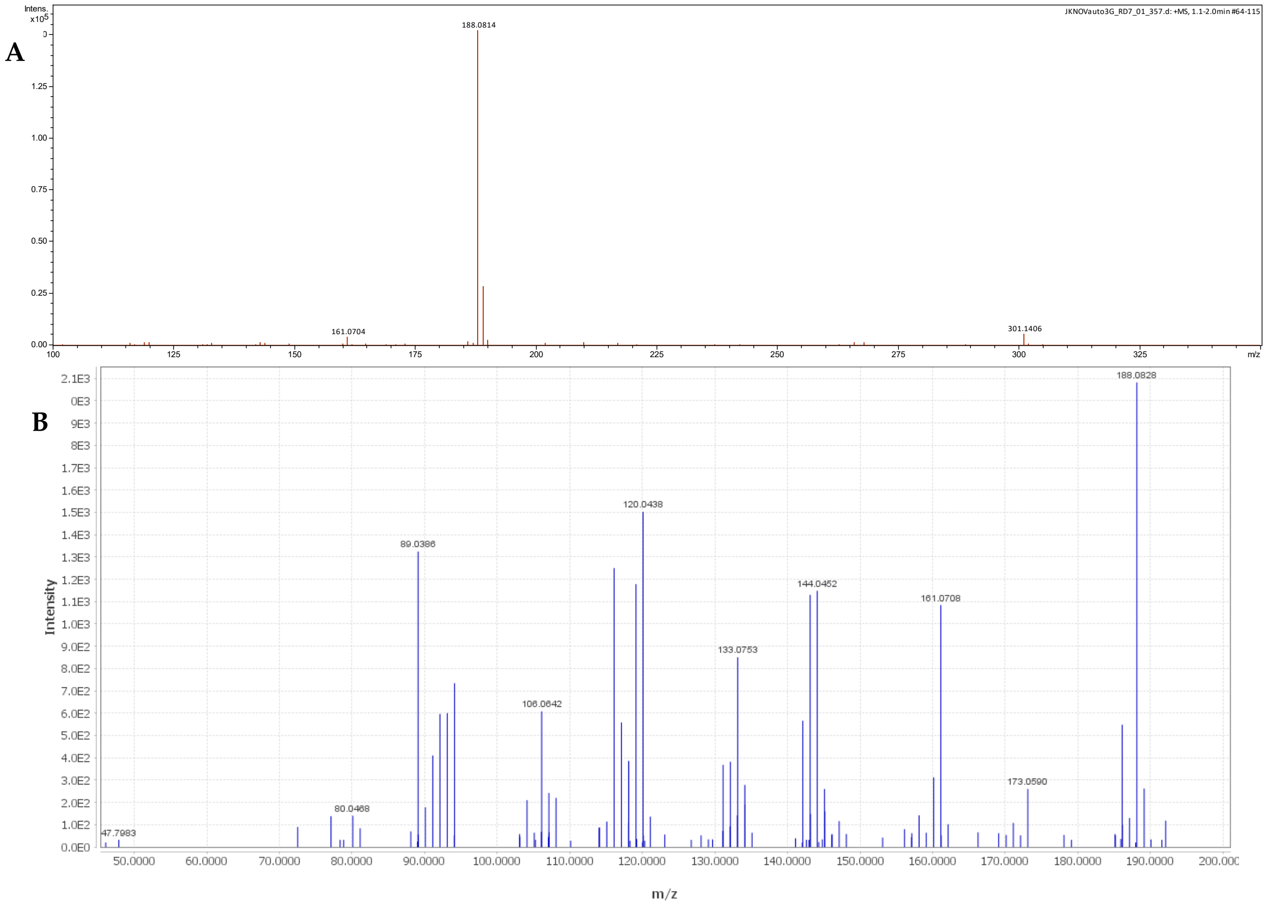

S.5.3 Mass spectra (**A** ms[1]; **B** ms[2]) for makaluvamine I (**3**).

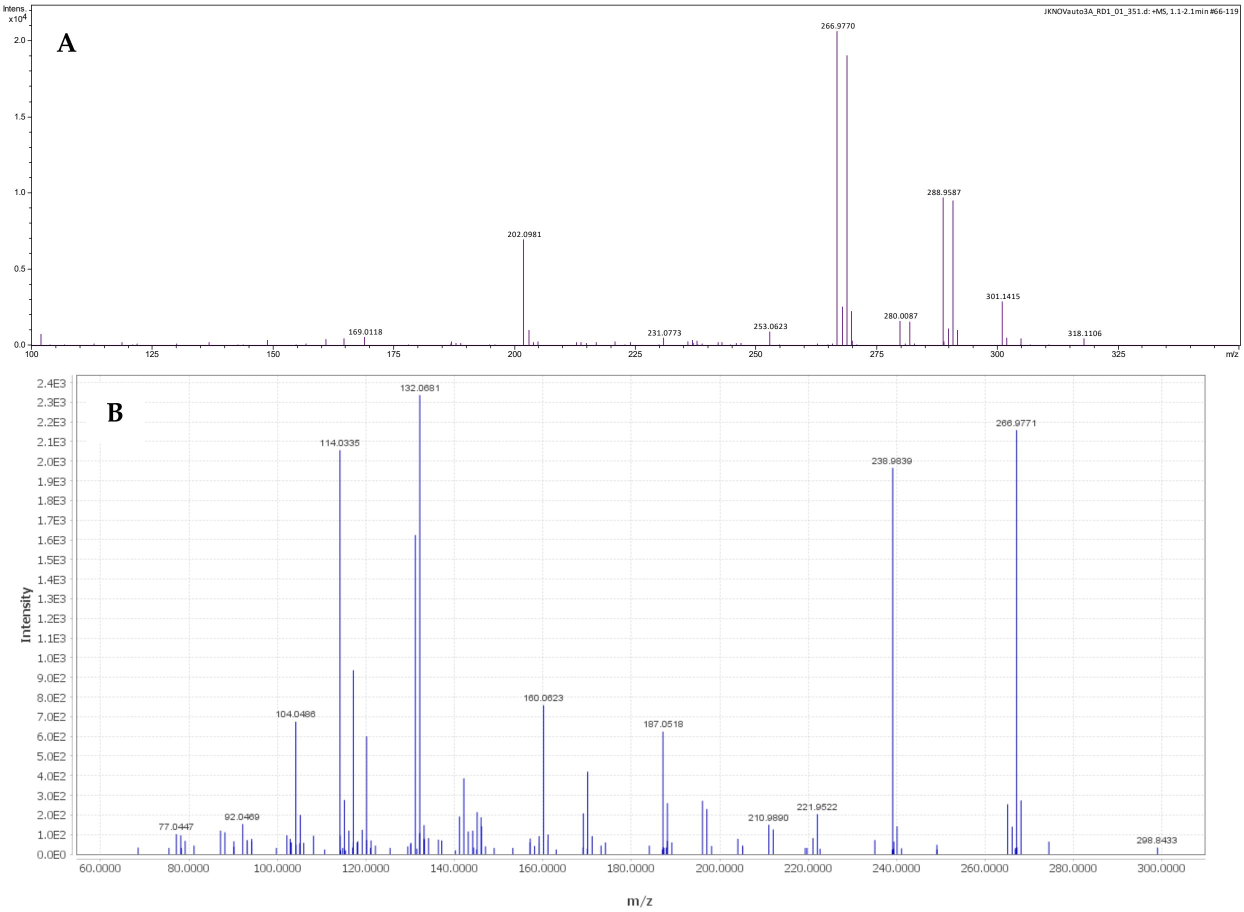

S.5.4 Mass spectra (**A** ms[1]; **B** ms[2]) for makaluvamine O (**4**).

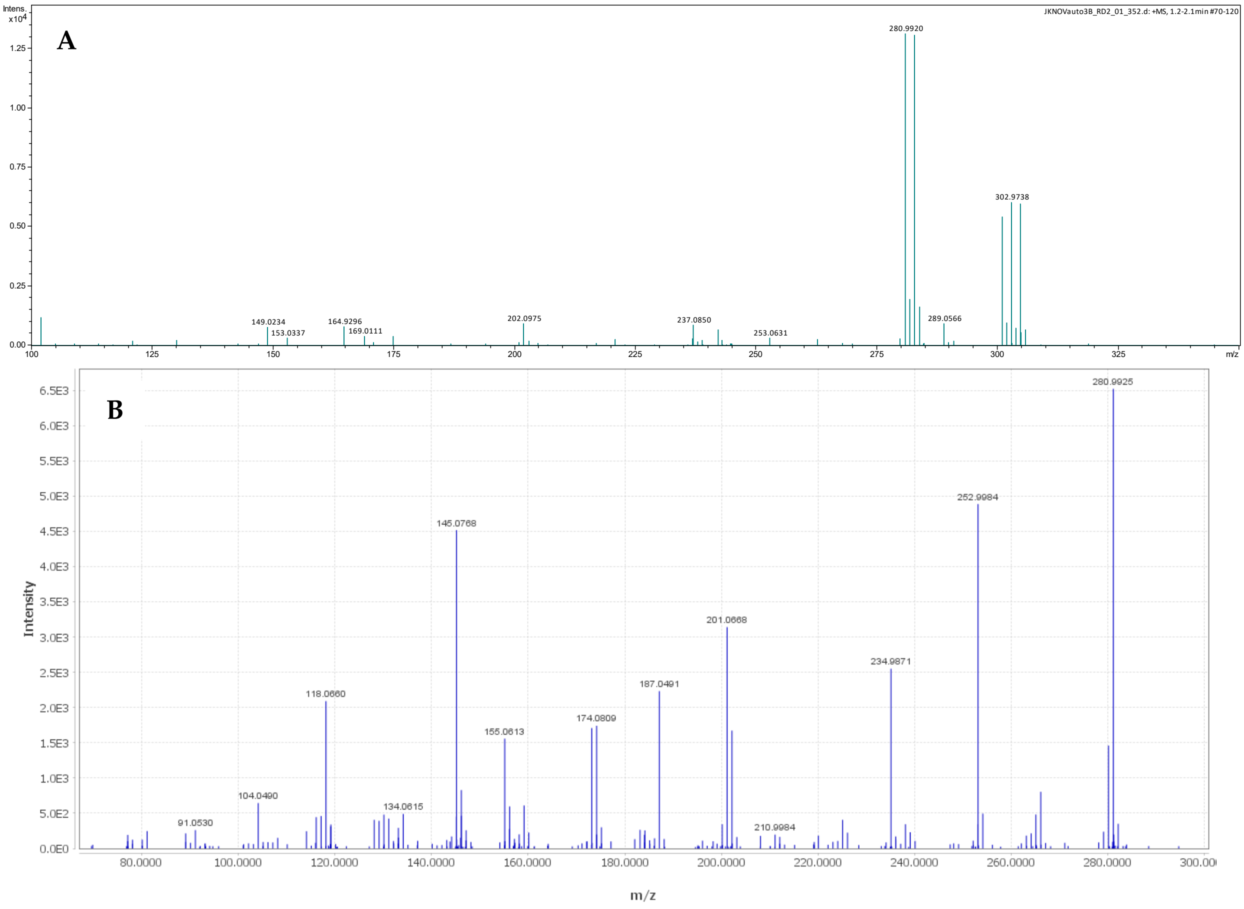

S.5.5 Mass spectra (**A** ms[1]; **B** ms[2]) for makaluvone (**5**).

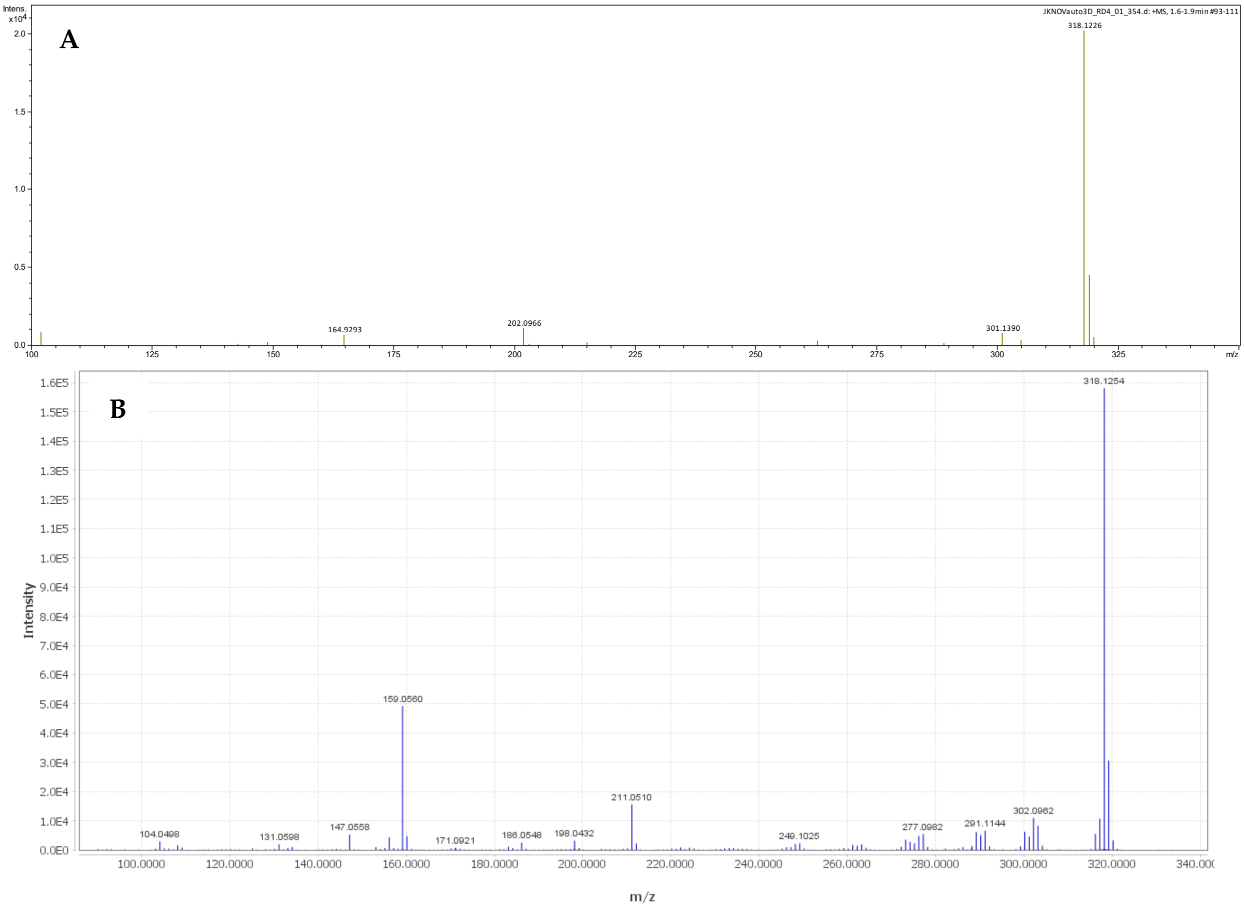

S.5.6 Mass spectra (**A** ms¹; **B** ms²) for tsitsikammamine B (**6**).

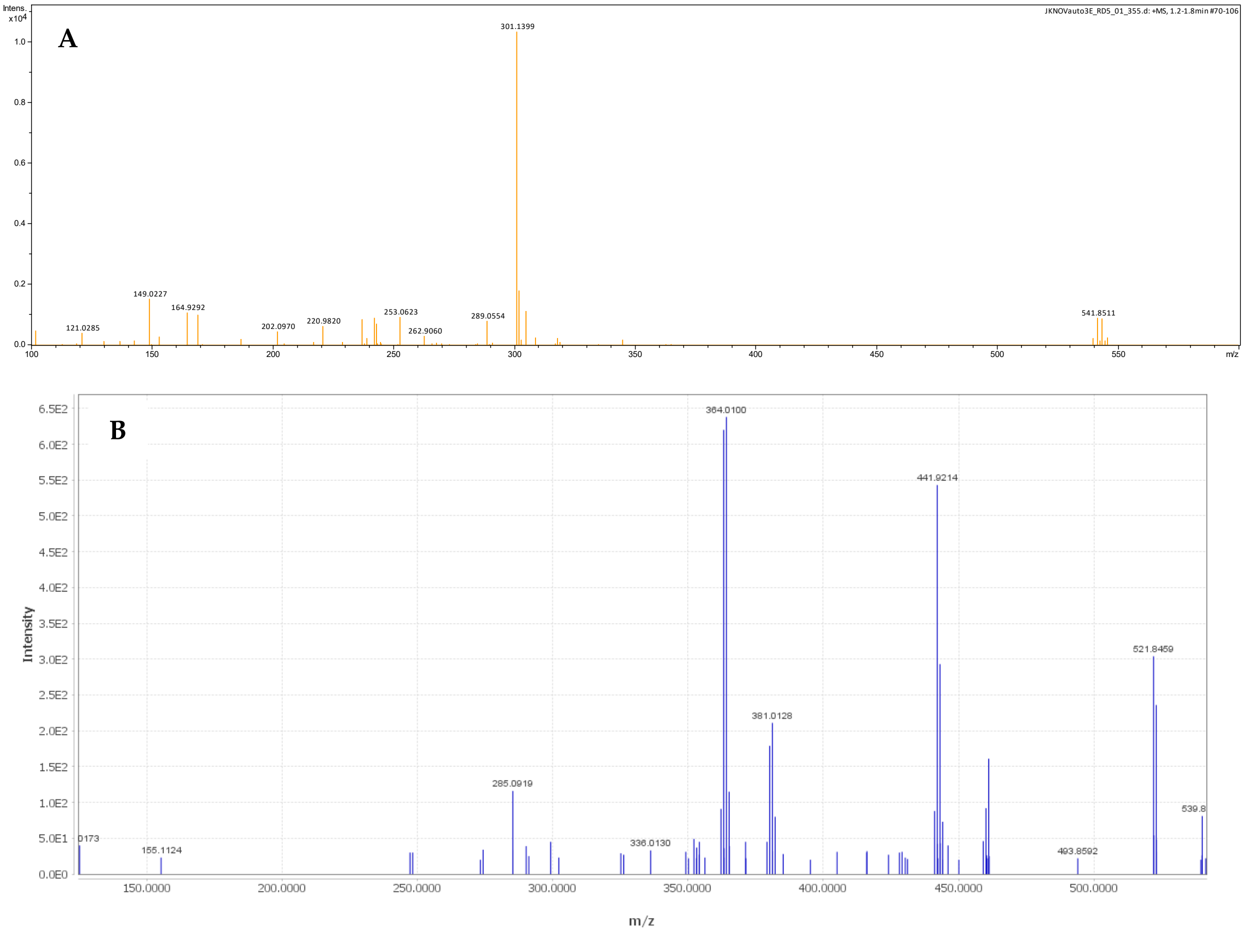

S.5.7 Mass spectra (**A** ms¹; **B** ms²) for 14-bromo-3-dihydro-7,8-dehydrodiscorhabdin C (**7**).

Supplementary information 6 Infrared spectroscopy

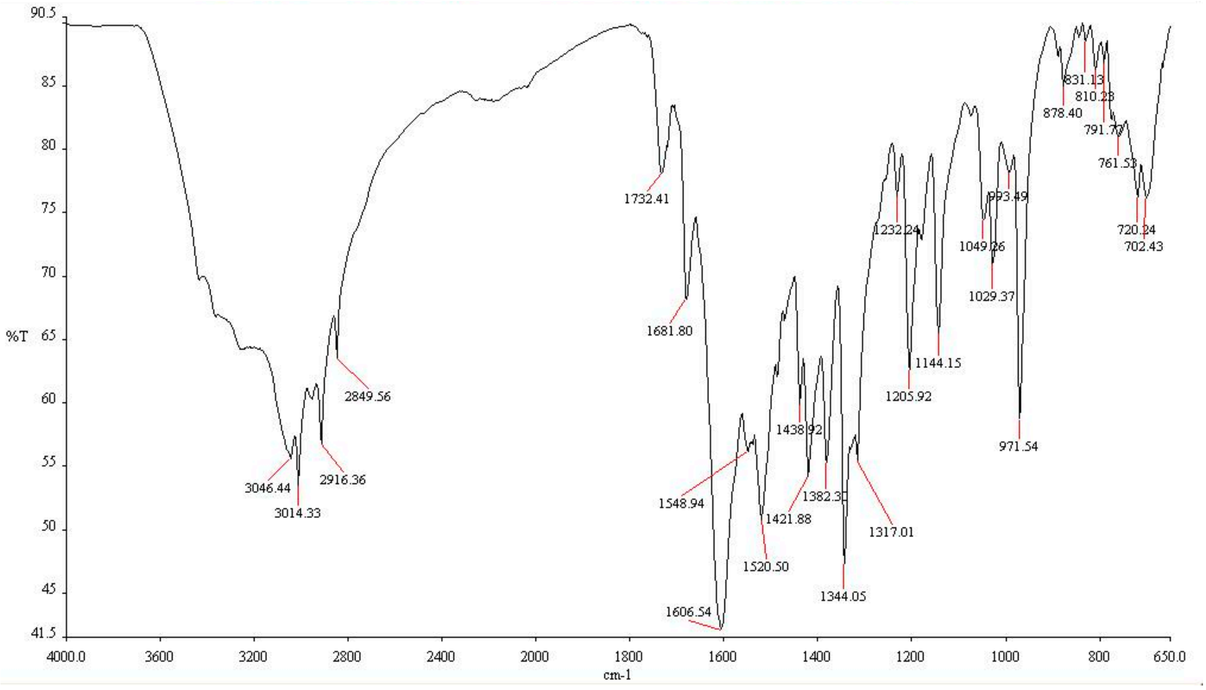

S.6 IR spectrum for makaluvamine Q (**1**).

Supplementary information 7 NMR data for pyrroloiminoquinone isolates

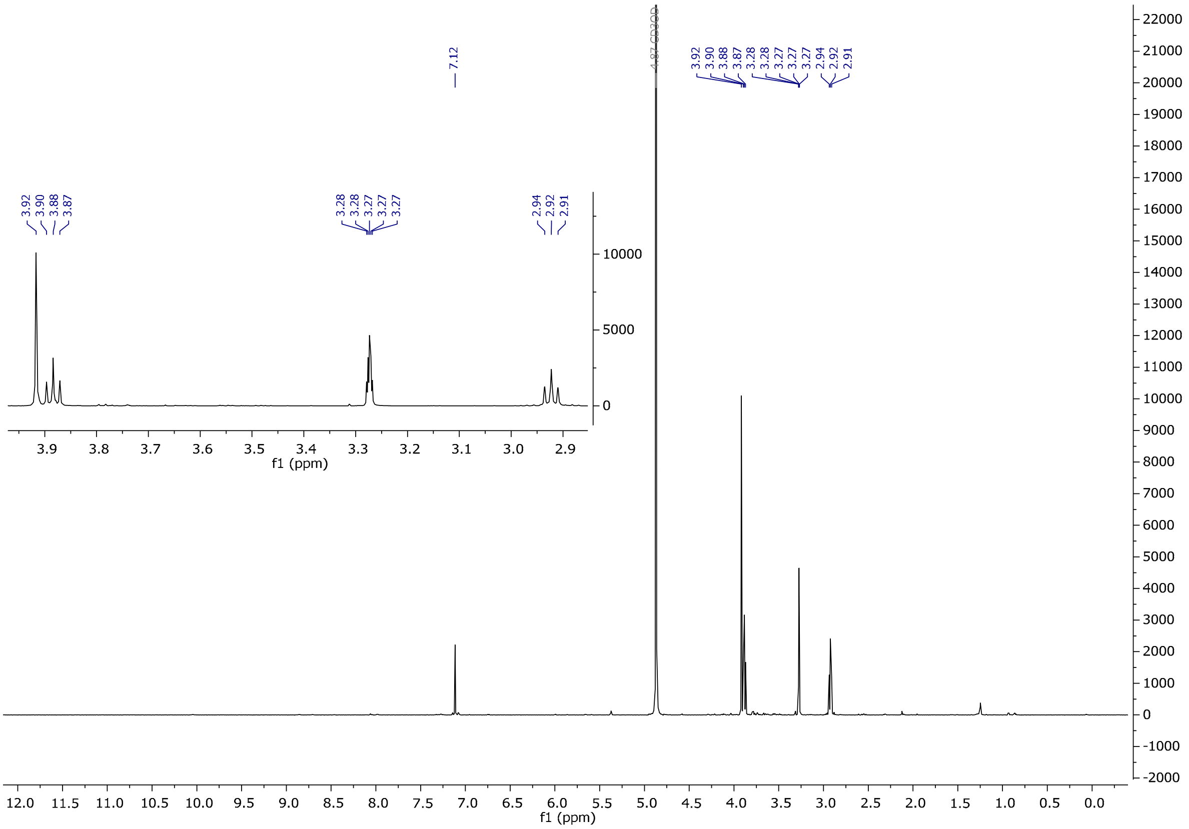

S.7.1 ^{1}H NMR spectrum for makaluvamine Q (**1**) in MeOD-d_4.

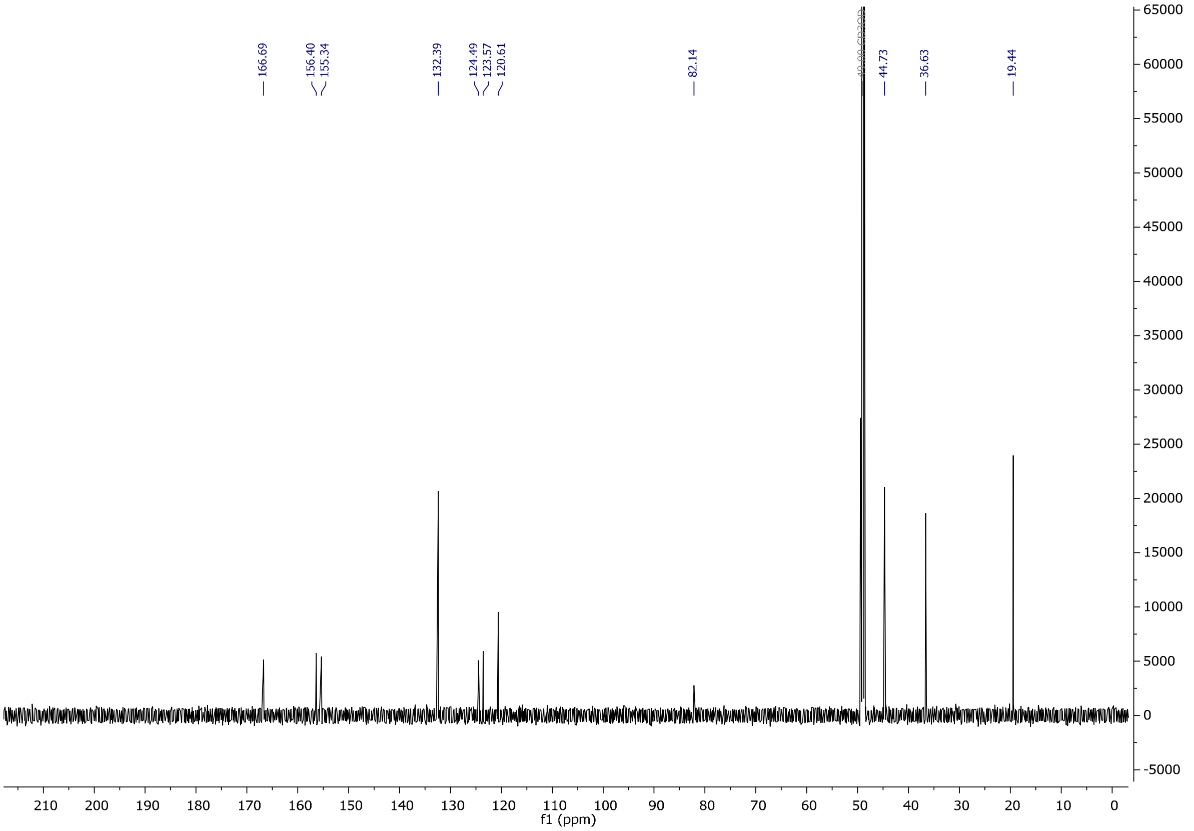

S.7.2 ^{13}C NMR spectrum for makaluvamine Q (**1**) in MeOD-d$_4$.

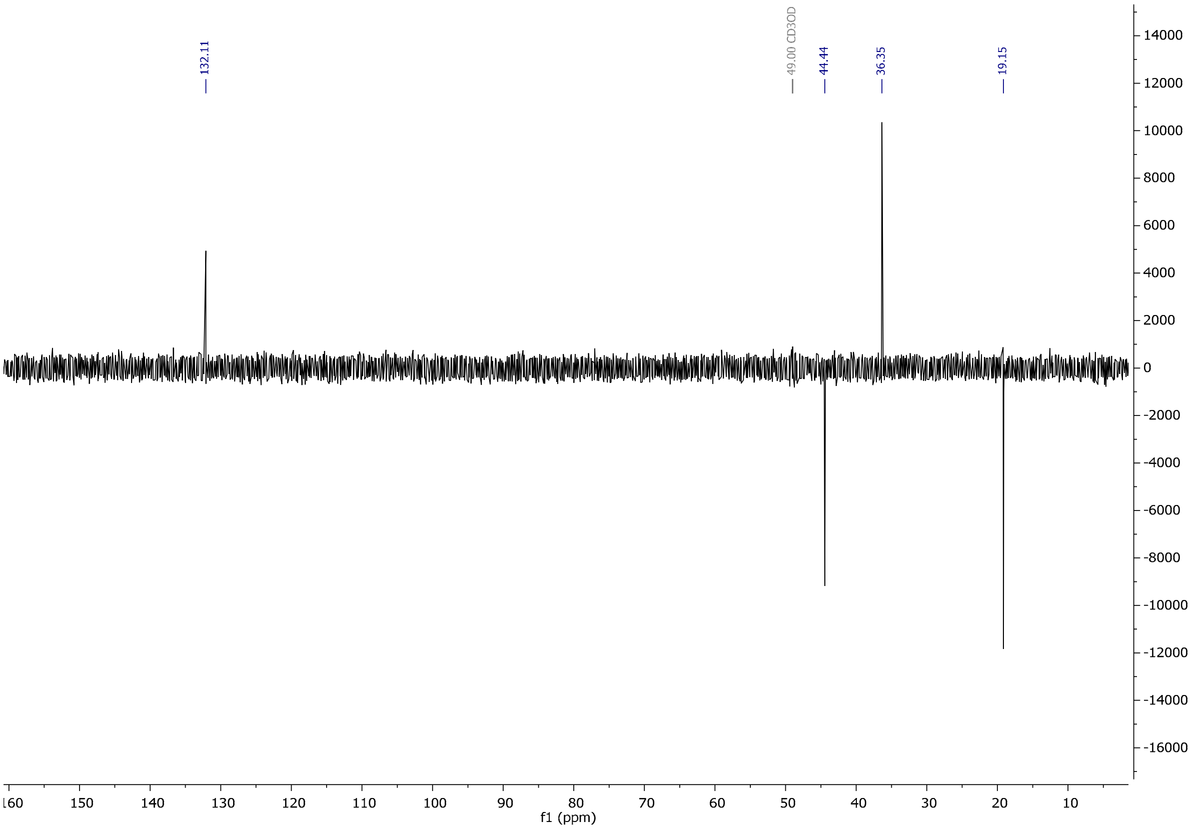

S.7.3 DEPT-135 spectrum for makaluvamine Q (**1**) in MeOD-d$_4$.

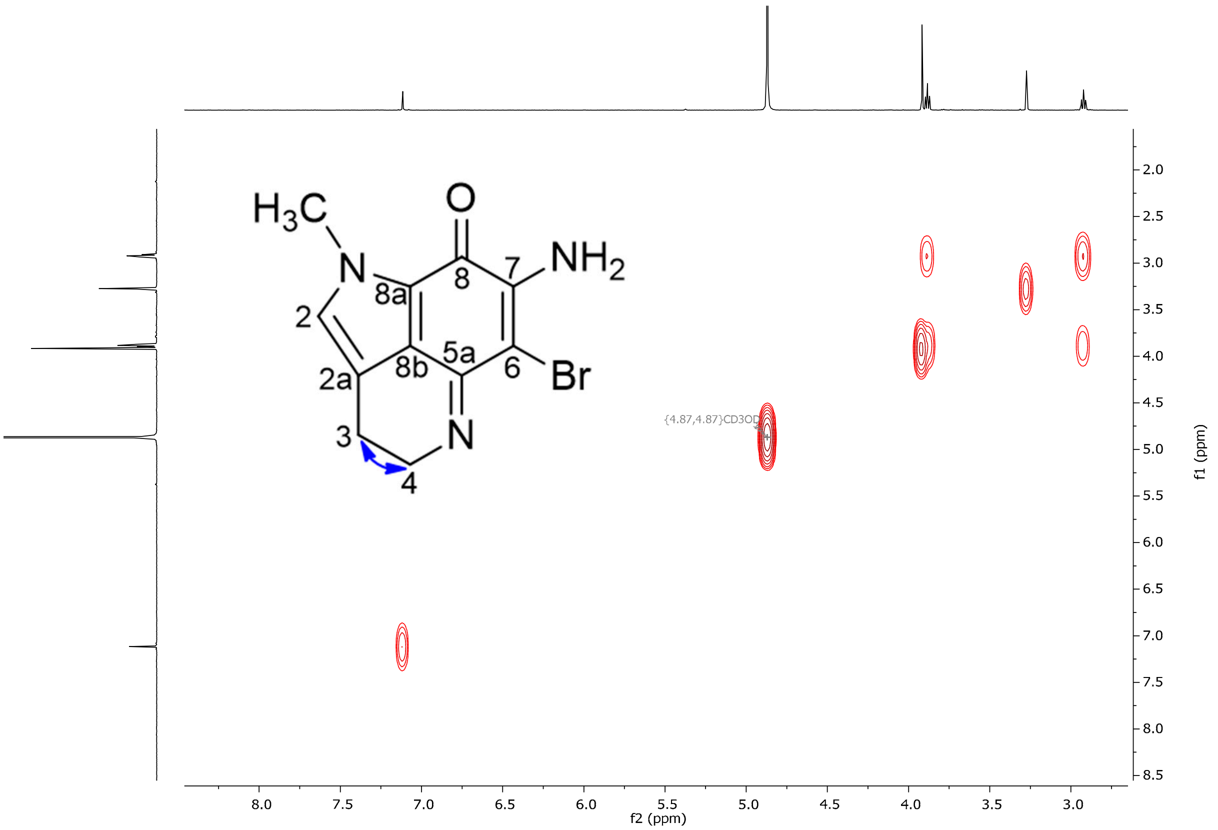

S.7.4 COSY spectrum of makaluvamine Q (**1**) in MeOD-d4 and chemical structure highlighting key COSY (^1H-^1H) correlations (arrows).

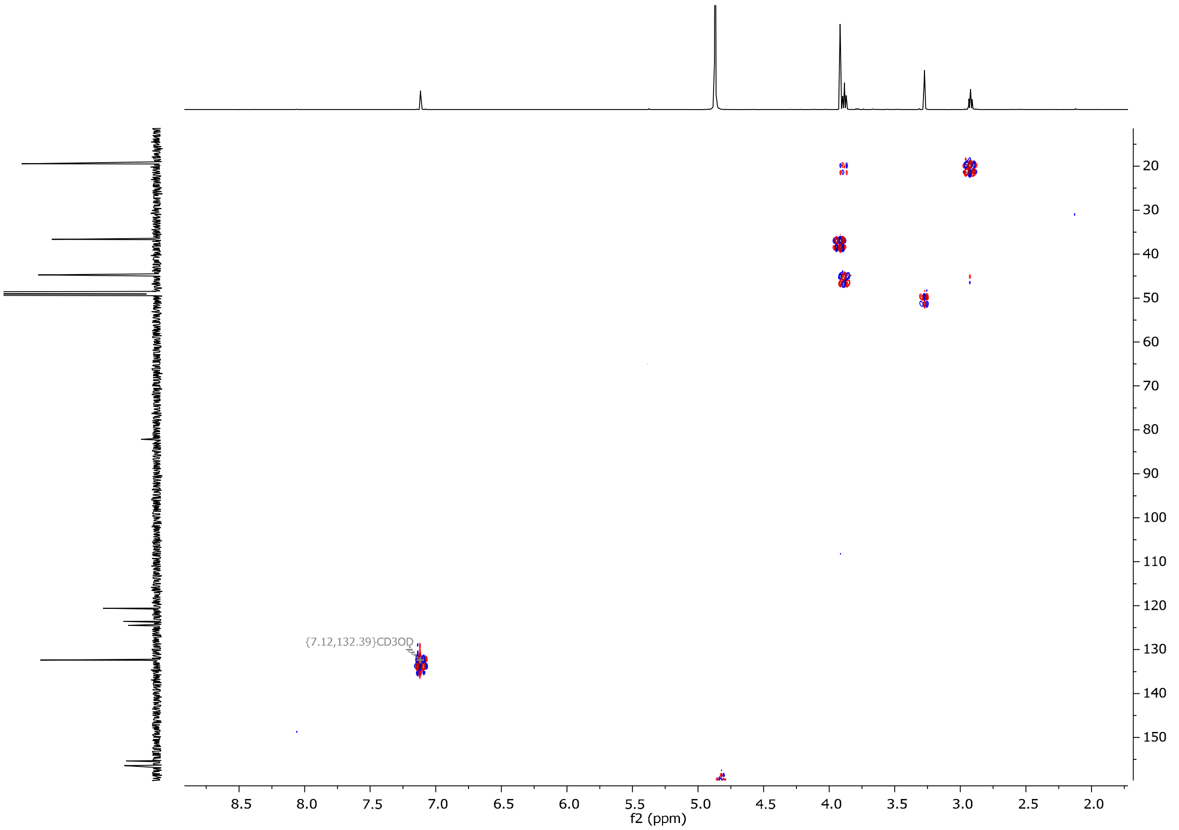

S.7.5 HSQC spectrum for makaluvamine Q (**1**) in MeOD-d4.

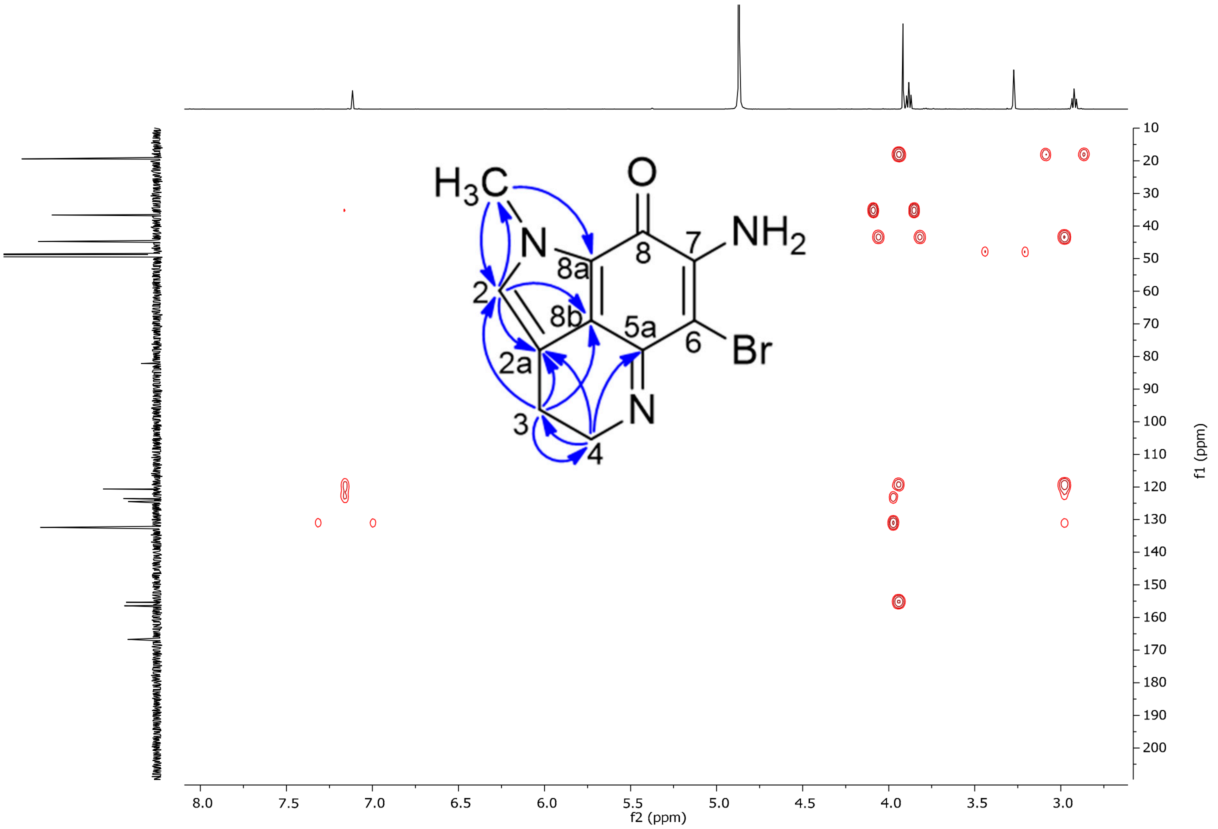

S.7.6 HMBC spectrum for makaluvamine Q (**1**) in MeOD-d4 and chemical structure highlighting key HMBC (^1H-^{13}C) correlations.(arrows).

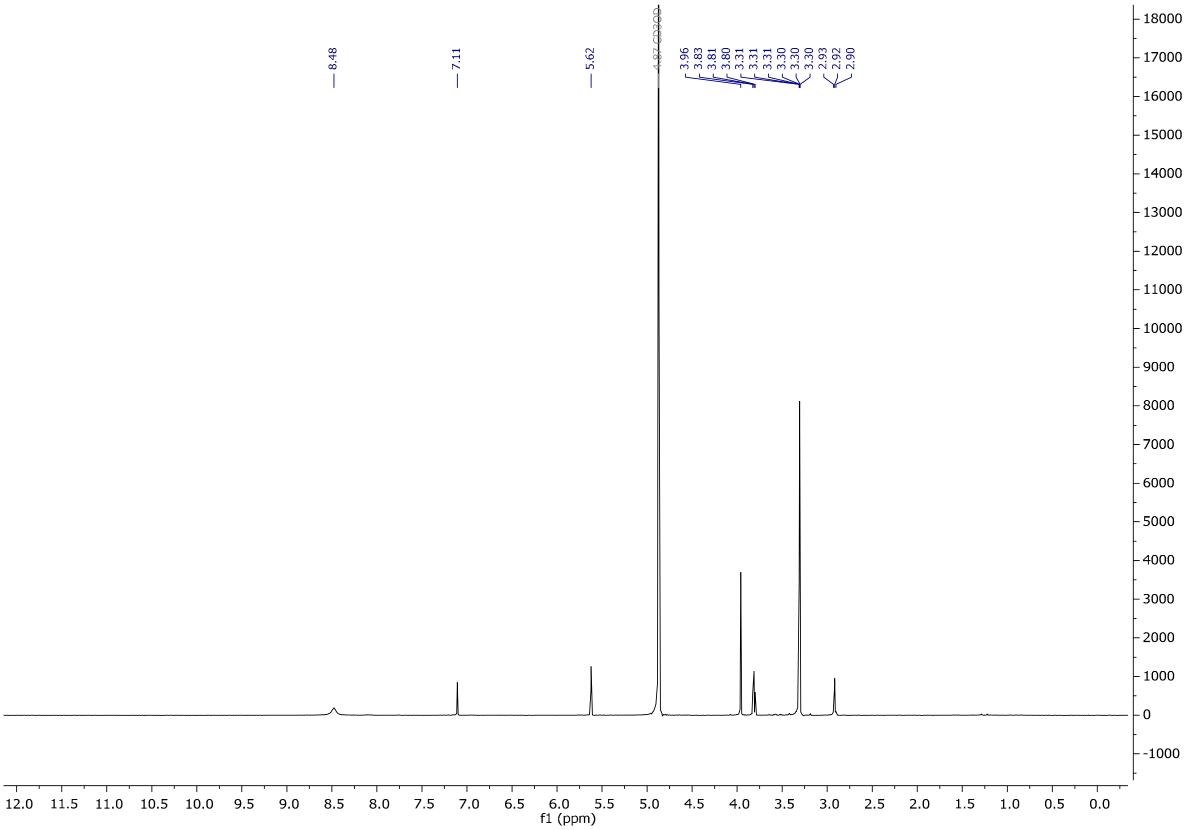

S.7.7 ^1H NMR spectrum for makaluvamine A (**2**) in MeOD-d4.

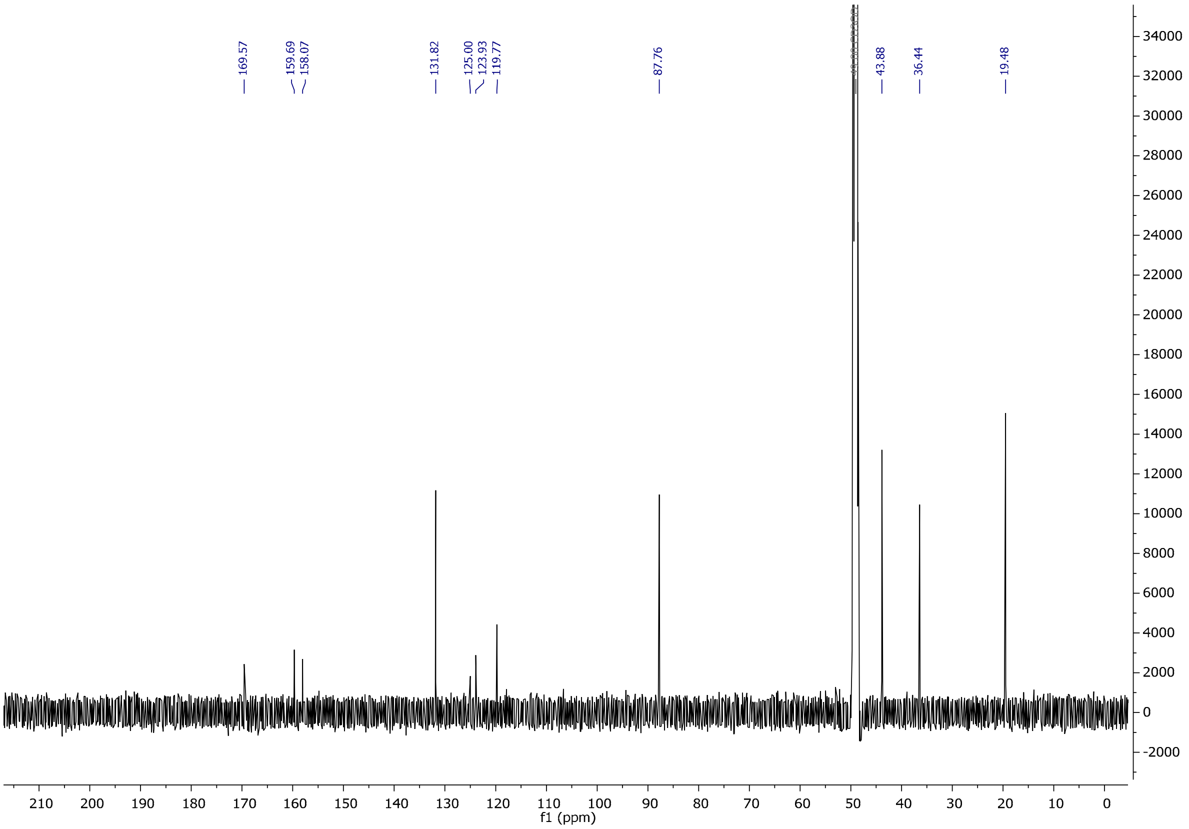

S.7.8 ^{13}C NMR spectrum for makaluvamine A (**2**) in MeOD-d4.

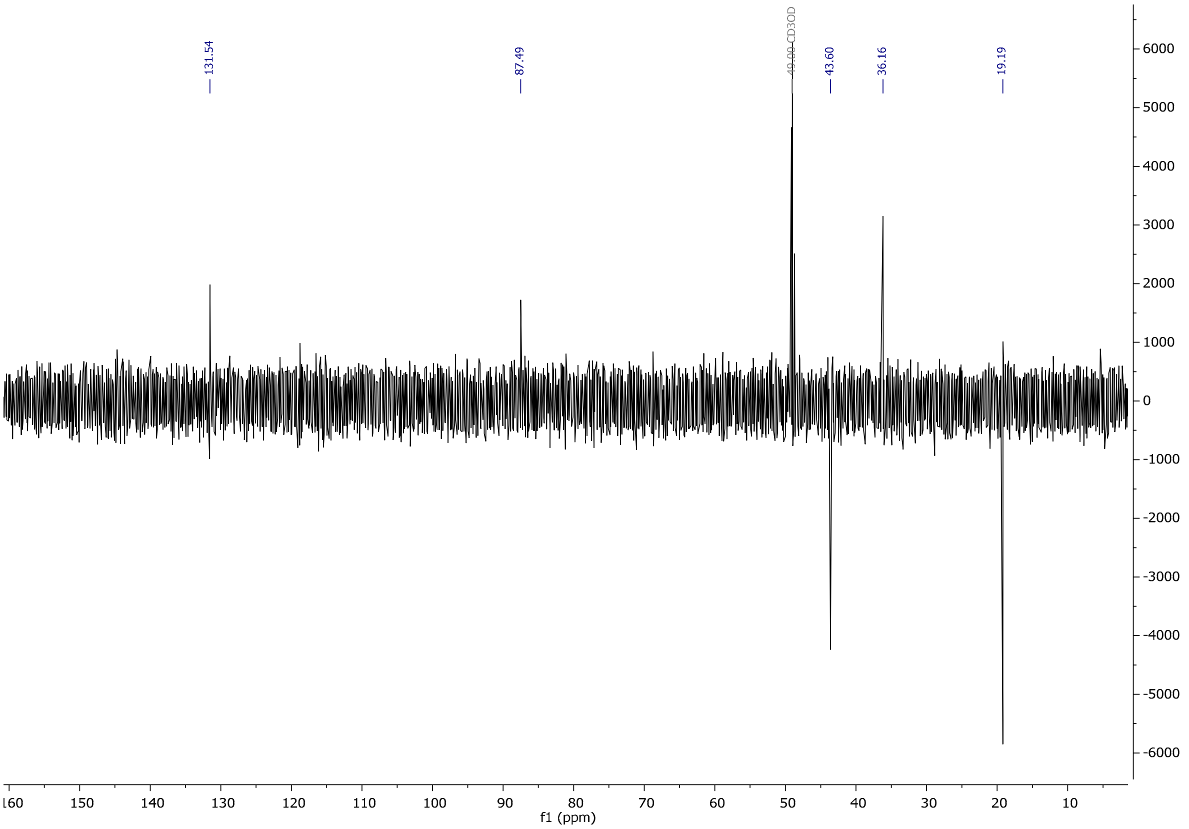

S.7.9 DEPT-135 spectrum for makaluvamine A (**2**) in MeOD-d4.

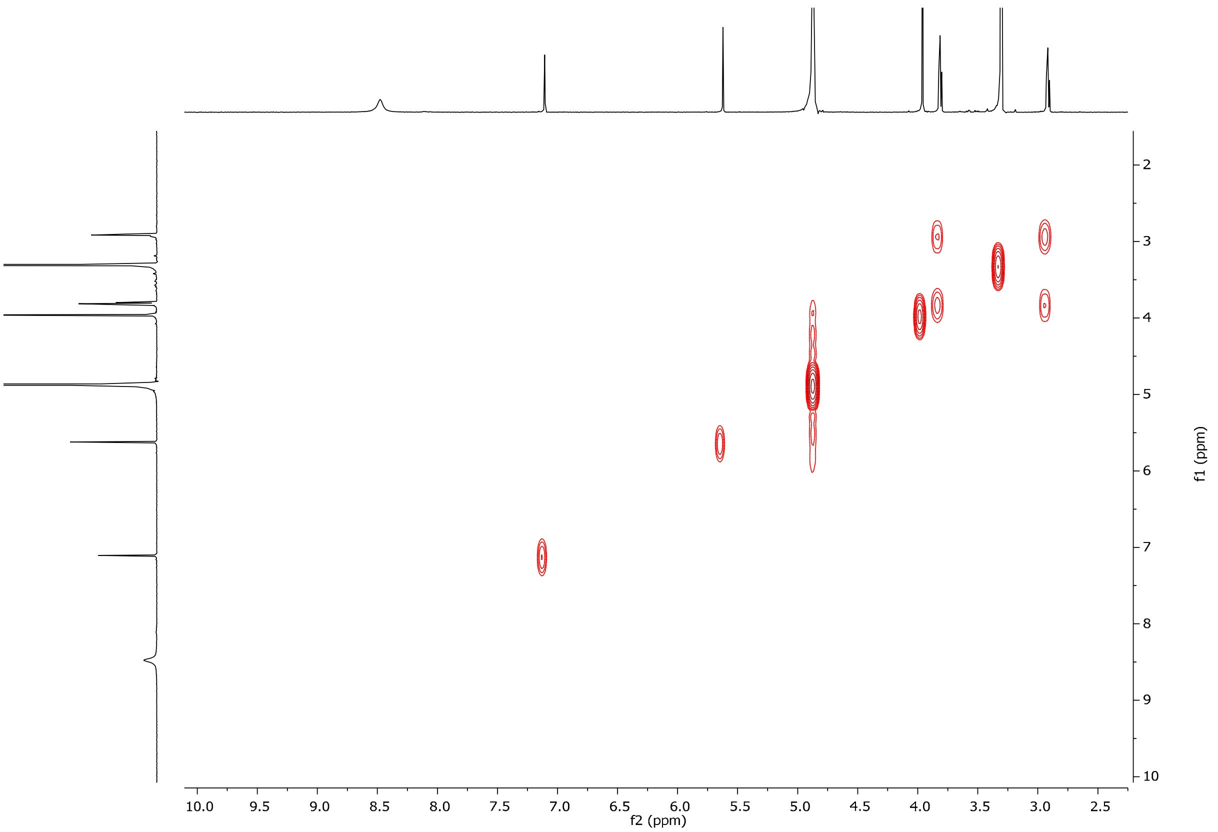

S.7.10 COSY spectrum for makaluvamine A (**2**) in MeOD-d_4.

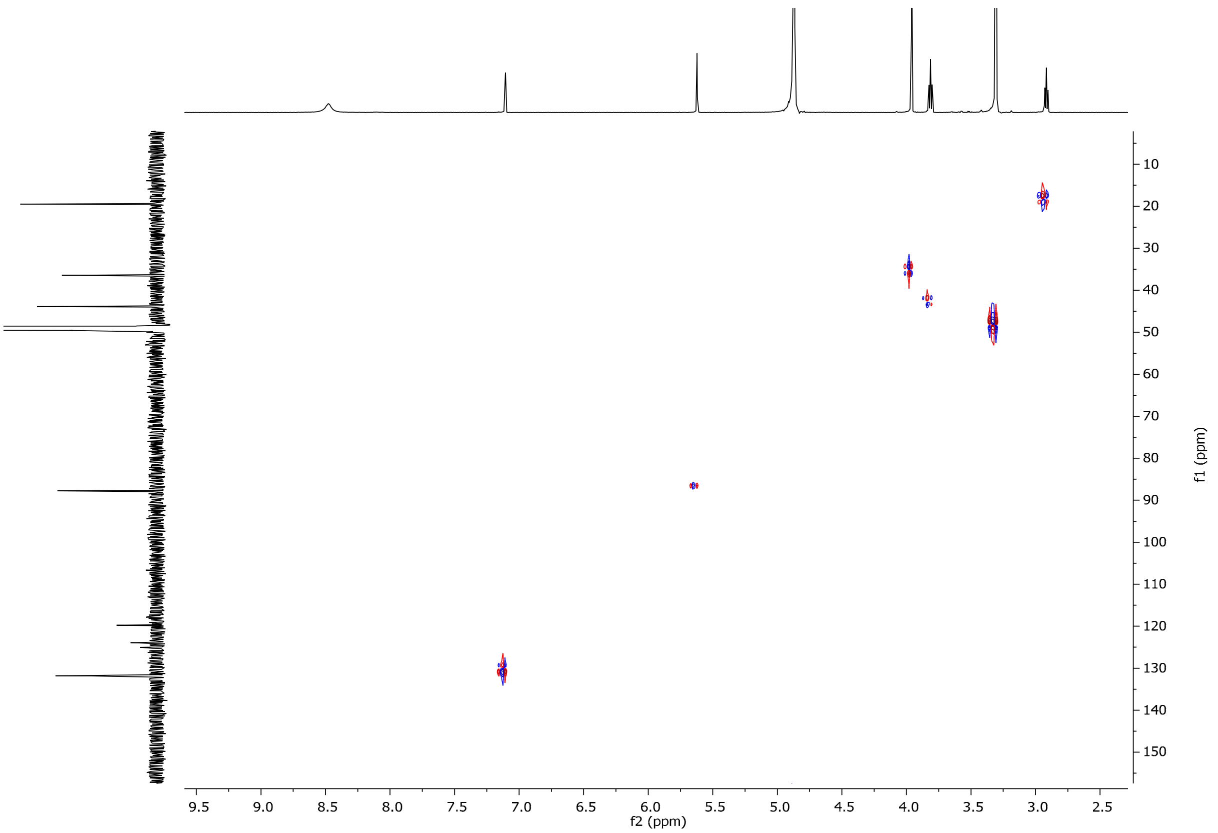

S.7.11 HSQC spectrum for makaluvamine A (**2**) in MeOD-d_4.

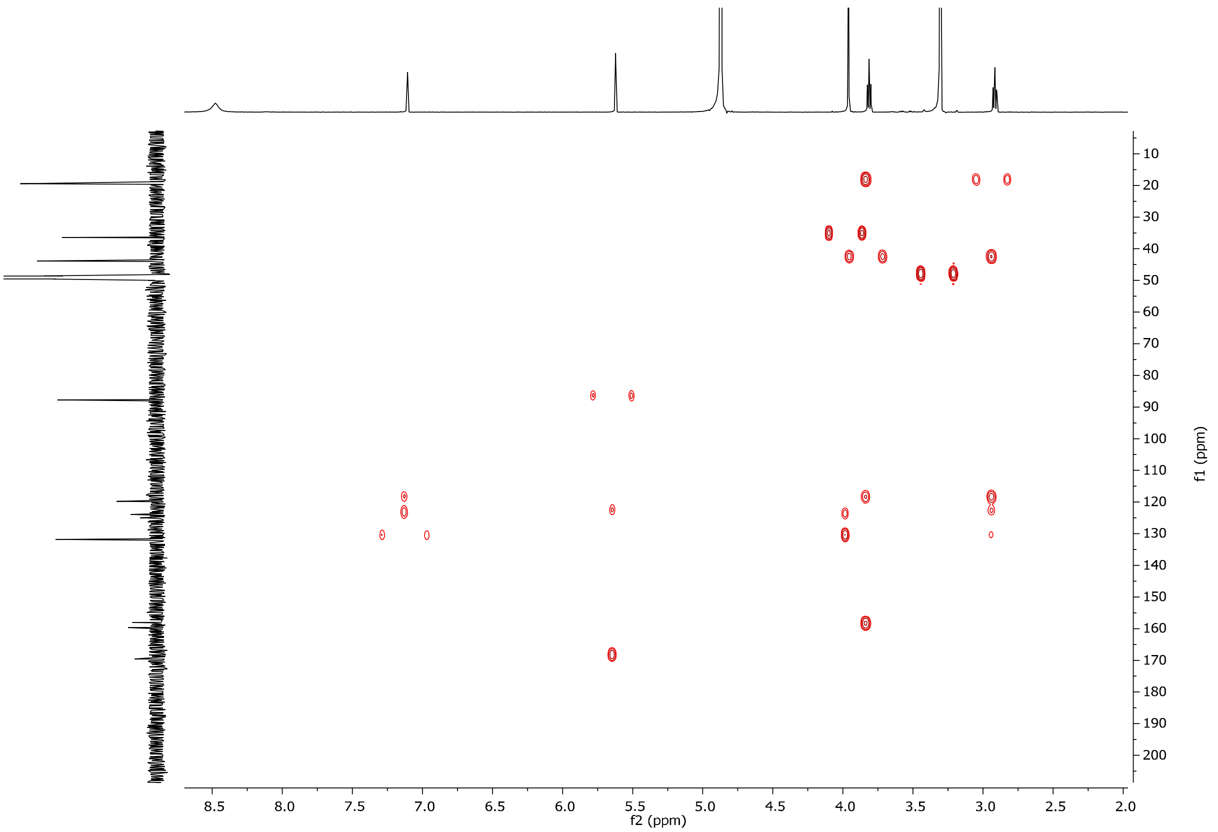

S.7.12 HMBC spectrum for makaluvamine A (**2**) in MeOD-d4.

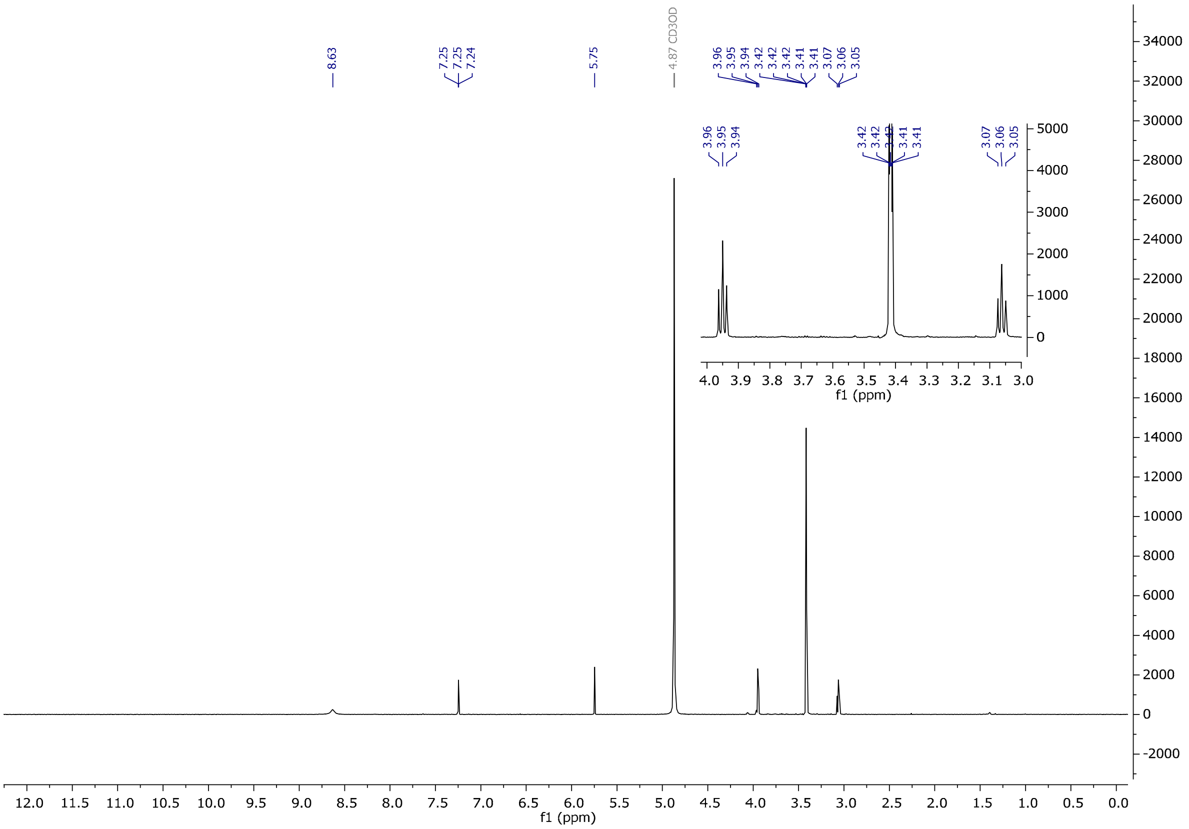

S.7.13 ^{1}H NMR spectrum for makaluvamine I (**3**) in MeOD-d4.

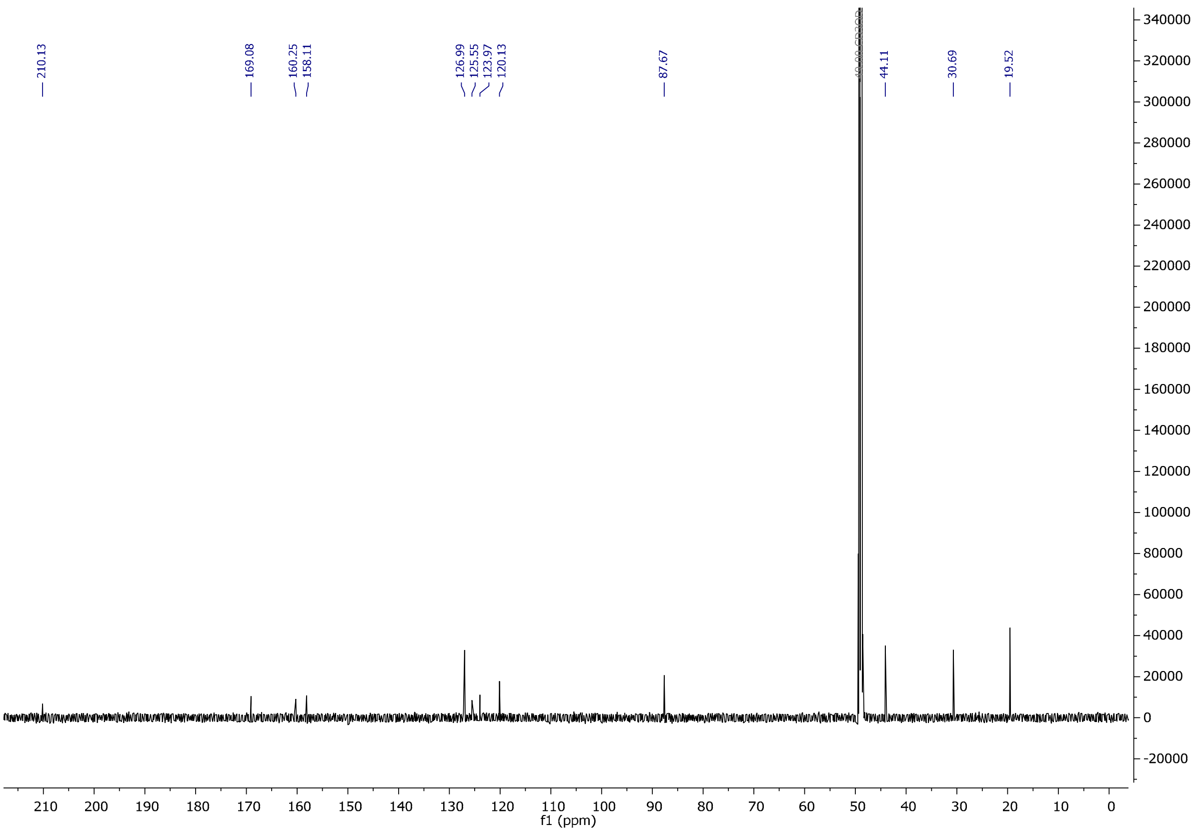

S.7.14 ^{13}C NMR spectrum for makaluvamine I (**3**) in MeOD-d$_4$.

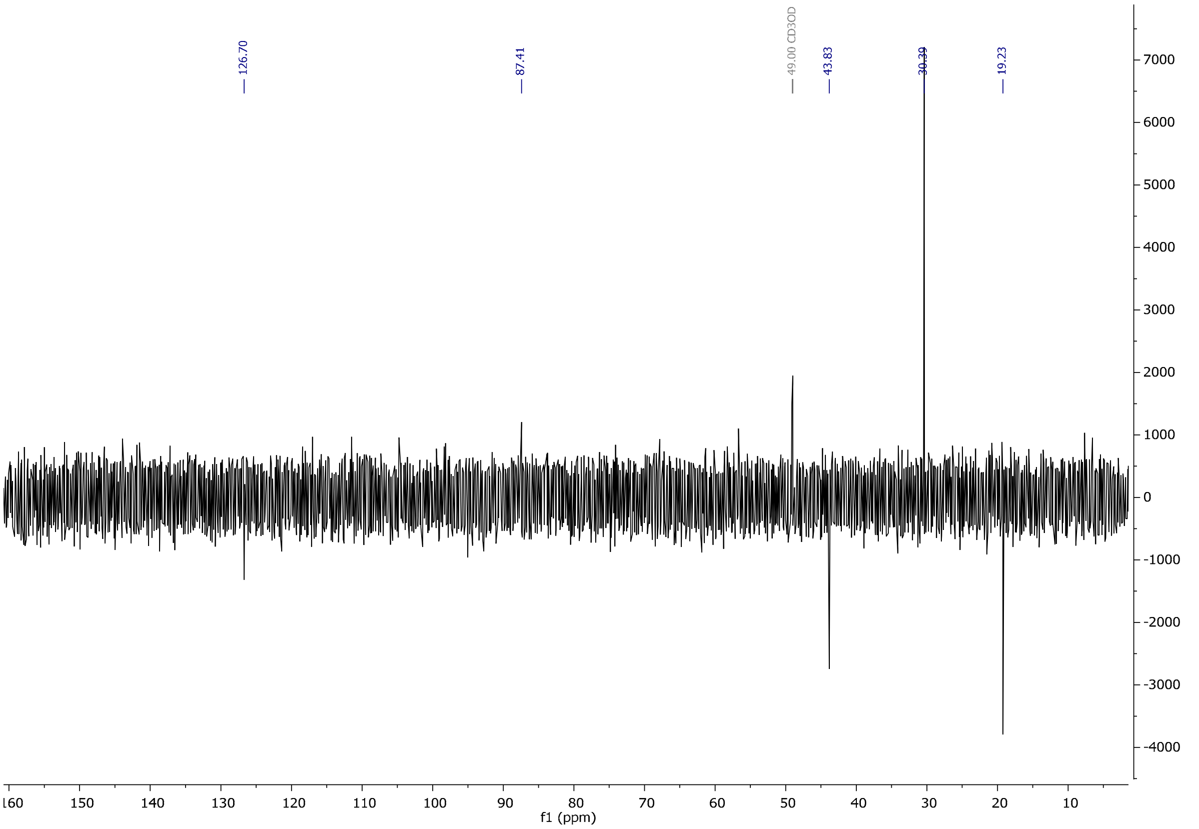

S.7.15 DEPT-135 spectrum for makaluvamine I (**3**) in MeOD-d$_4$.

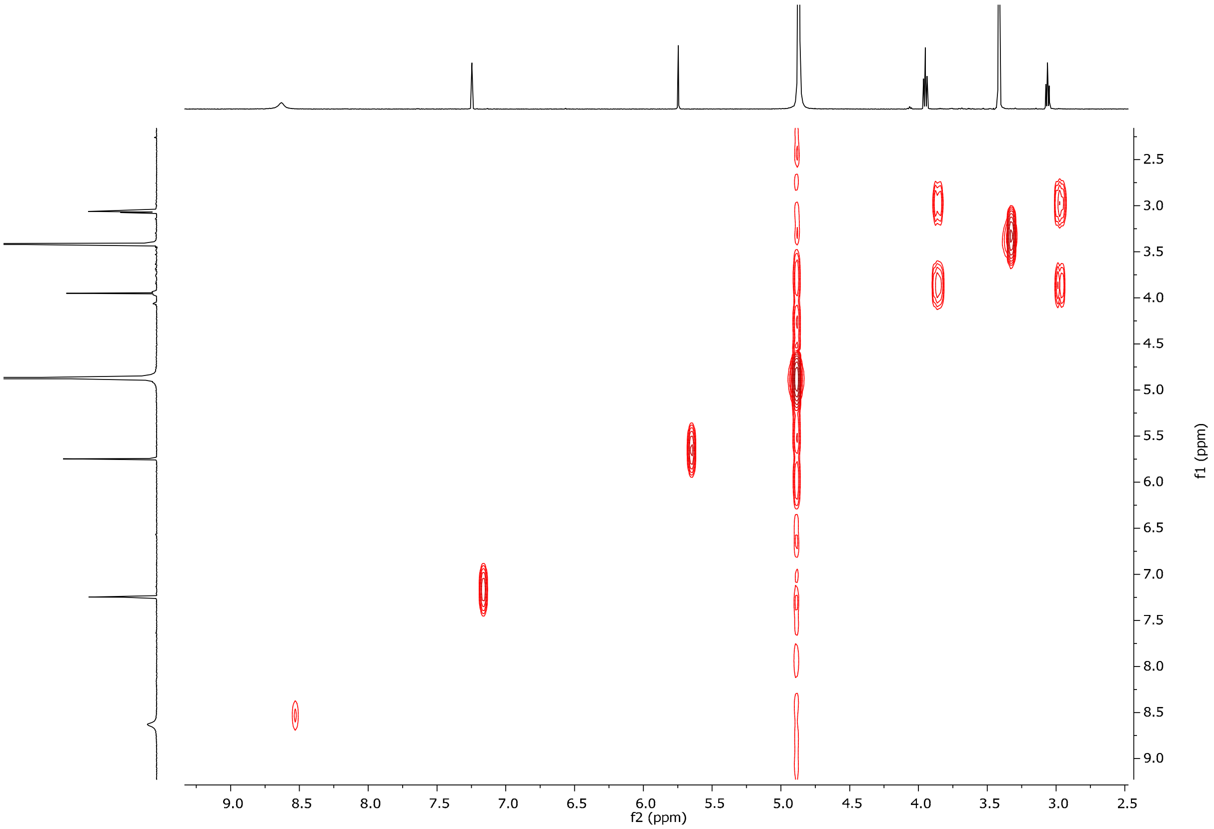

S.7.16 COSY spectrum for makaluvamine I (**3**) in MeOD-d4.

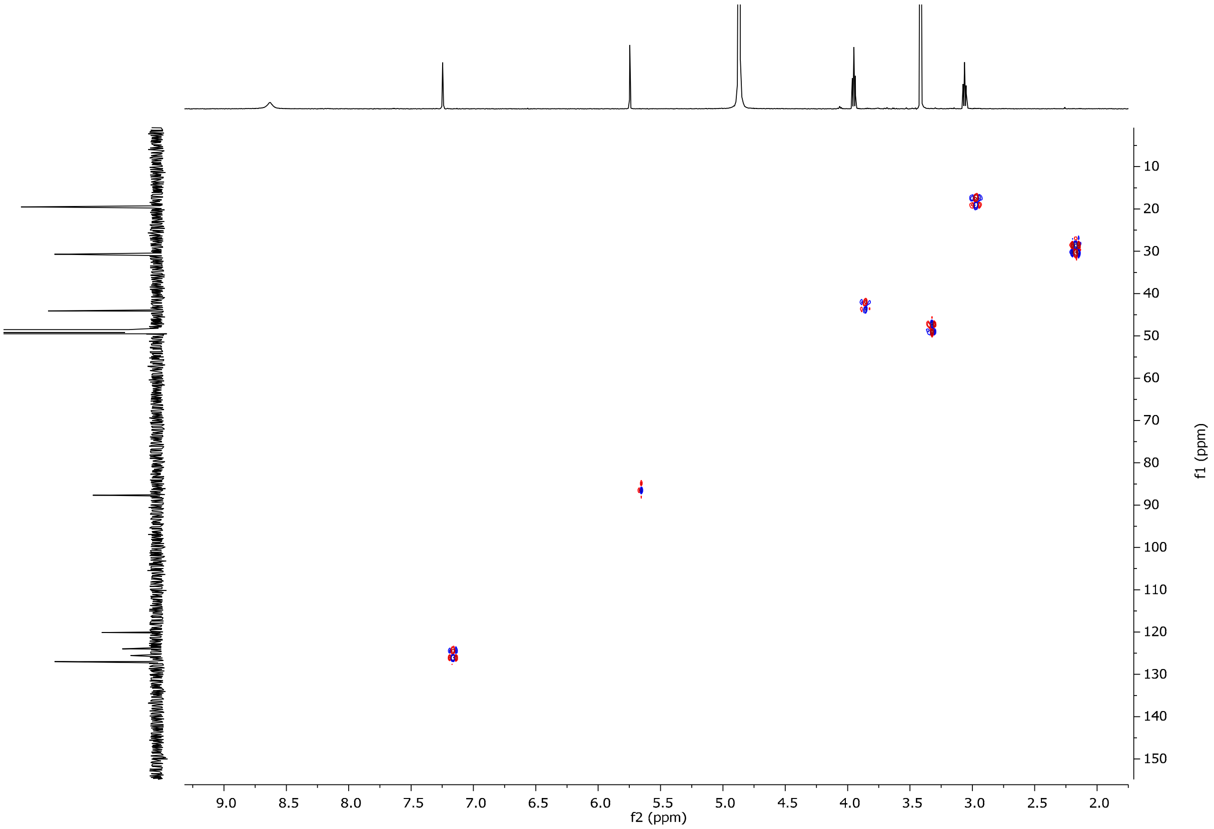

S.7.17 HSQC spectrum for makaluvamine I (**3**) in MeOD-d4.

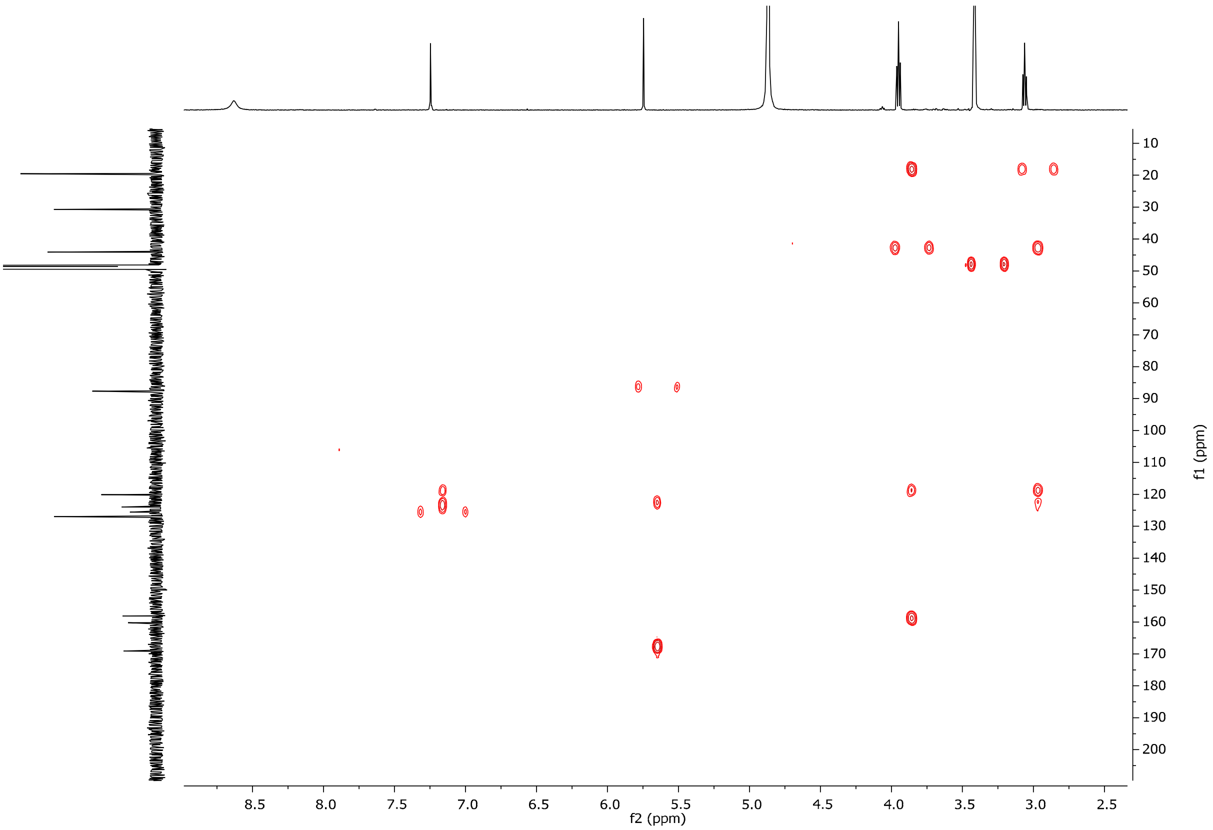

S.7.18 HMBC spectrum for makaluvamine I (**3**) in MeOD-d4.

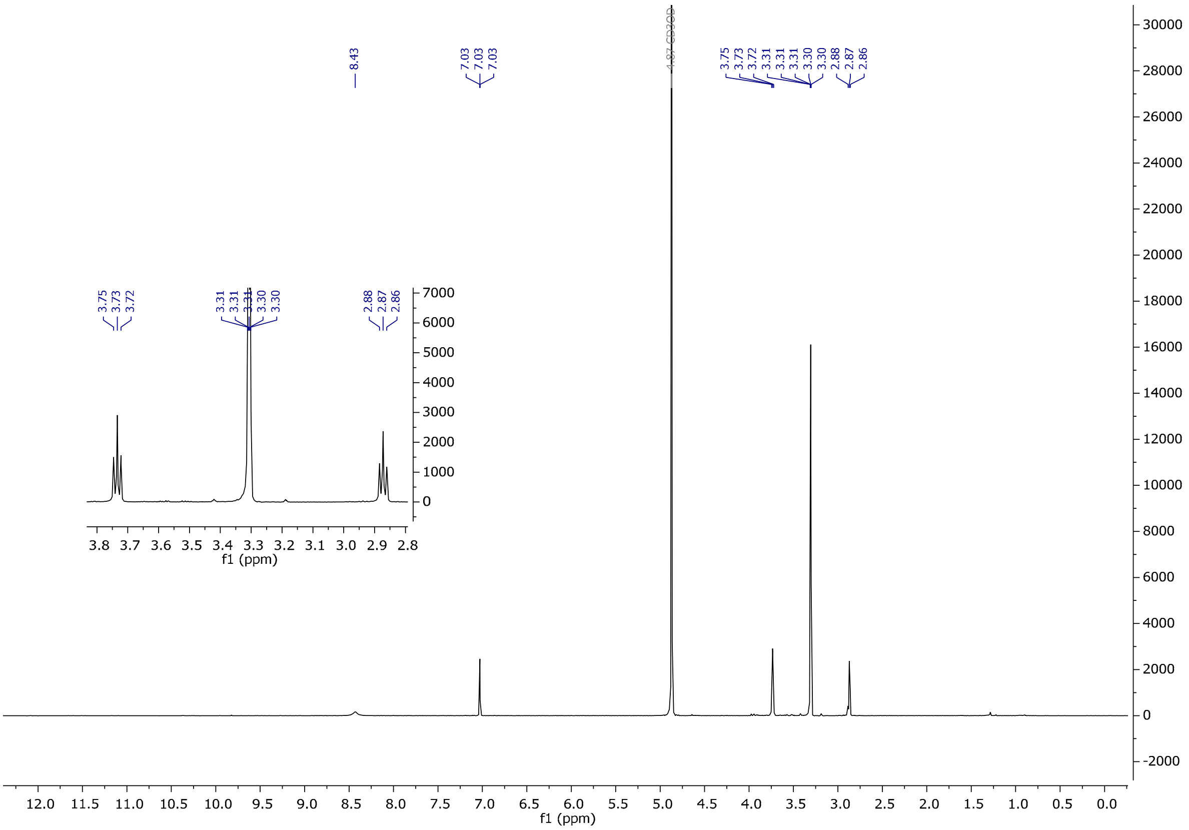

S.7.19 ^{1}H NMR spectrum for makaluvamine O (**4**) in MeOD-d4.

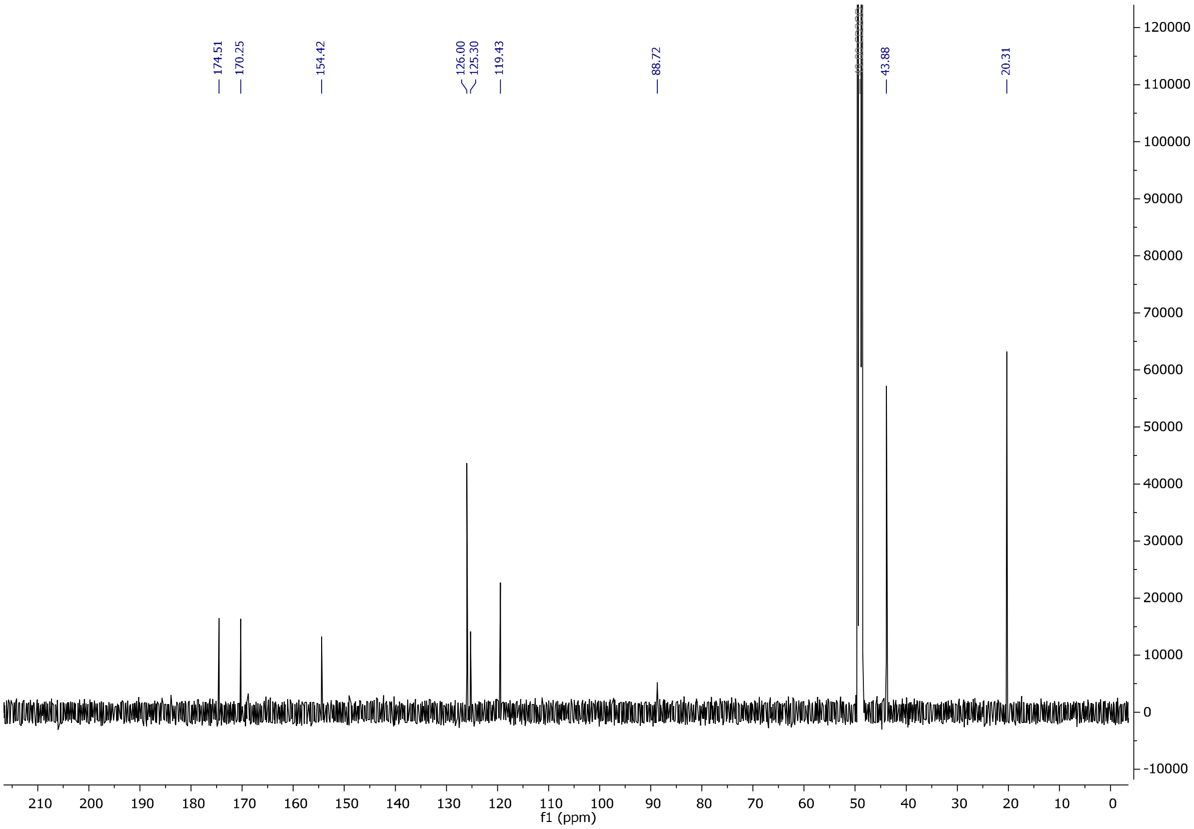

S.7.20 ^{13}C spectrum for makaluvamine O (**4**) in MeOD-d4.

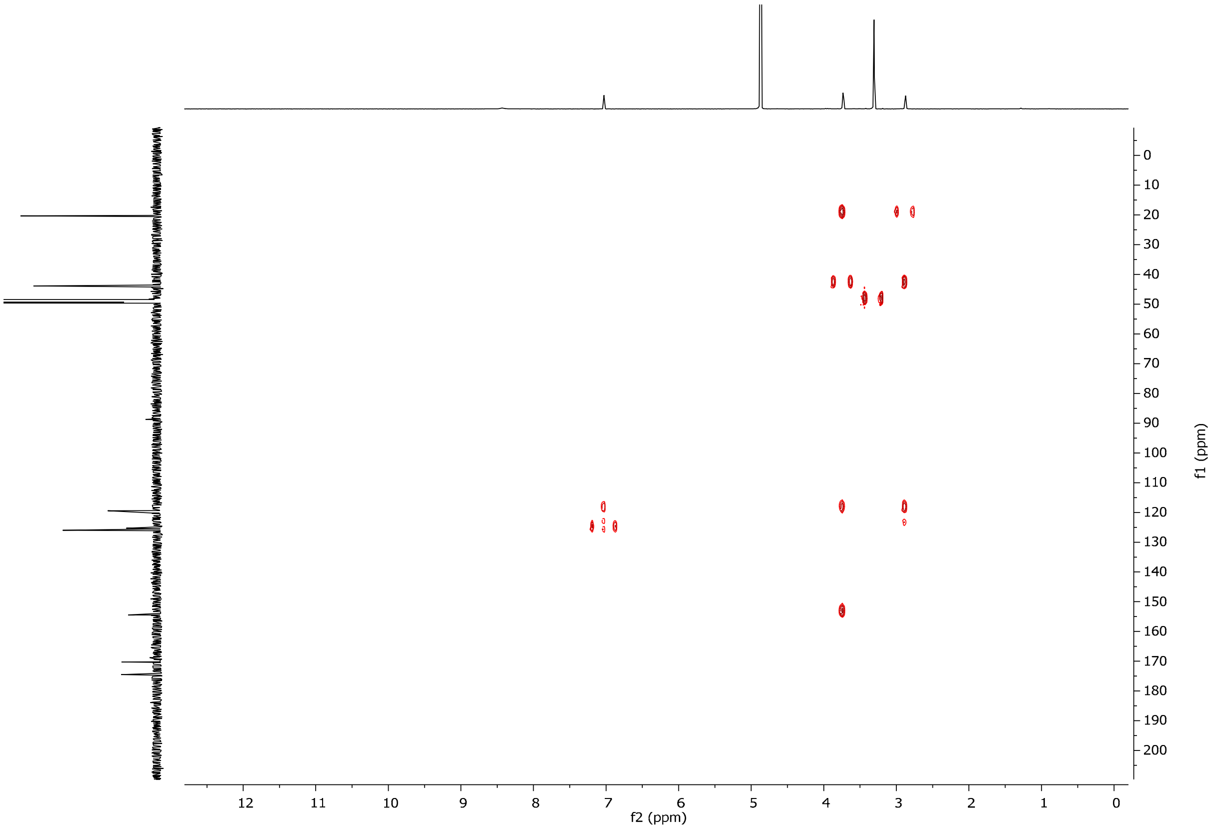

S.7.21 HMBC spectrum for makaluvamine E (**4**) in MeOD-d4.

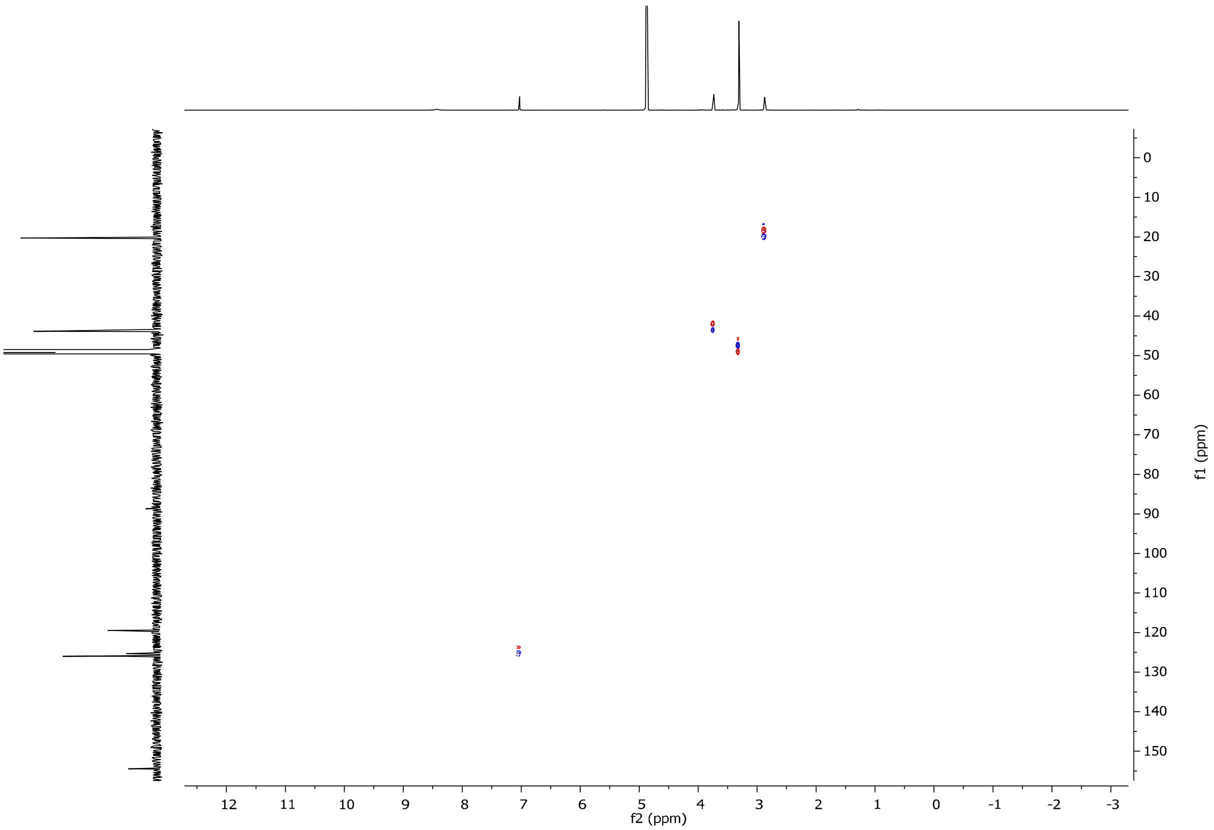

S.7.22 HSQC spectrum for makaluvamine E (**4**) in MeOD-d4.

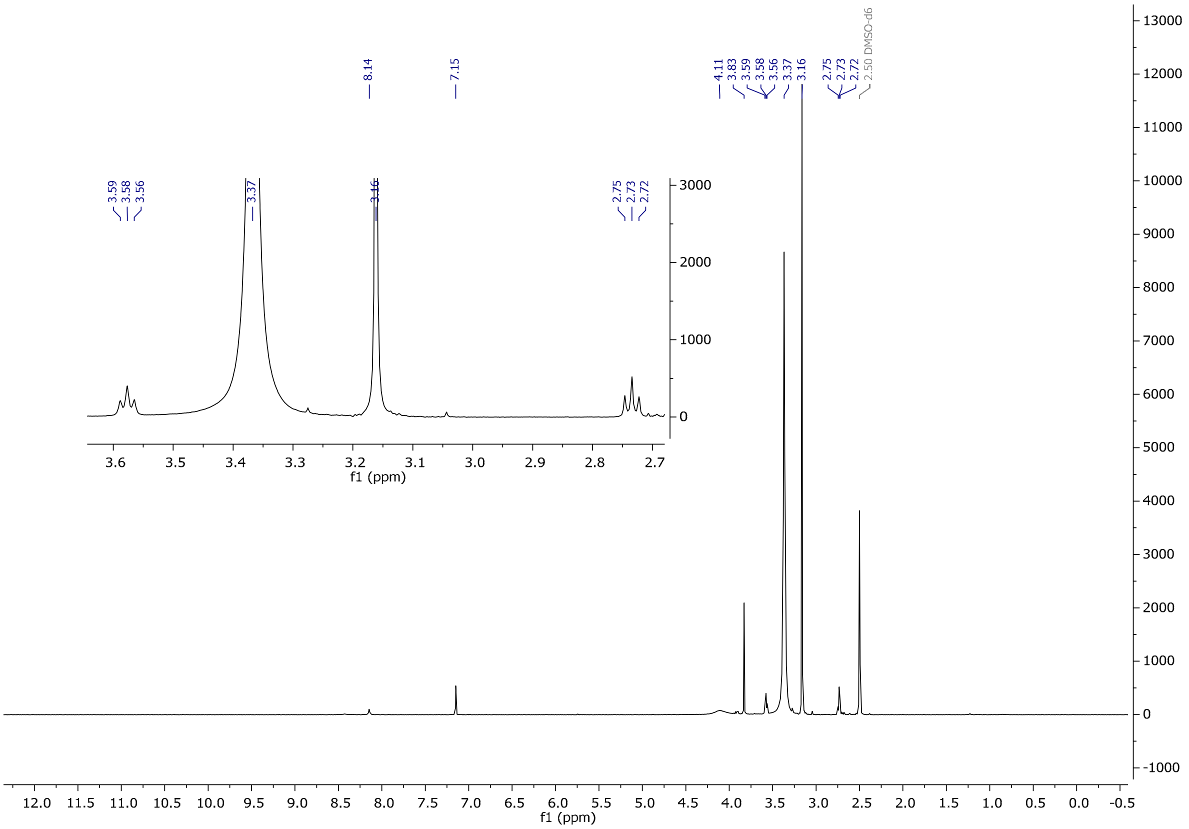

S.7.23 ^{1}H NMR spectrum for makaluvone (**5**) in DMSO-d6.

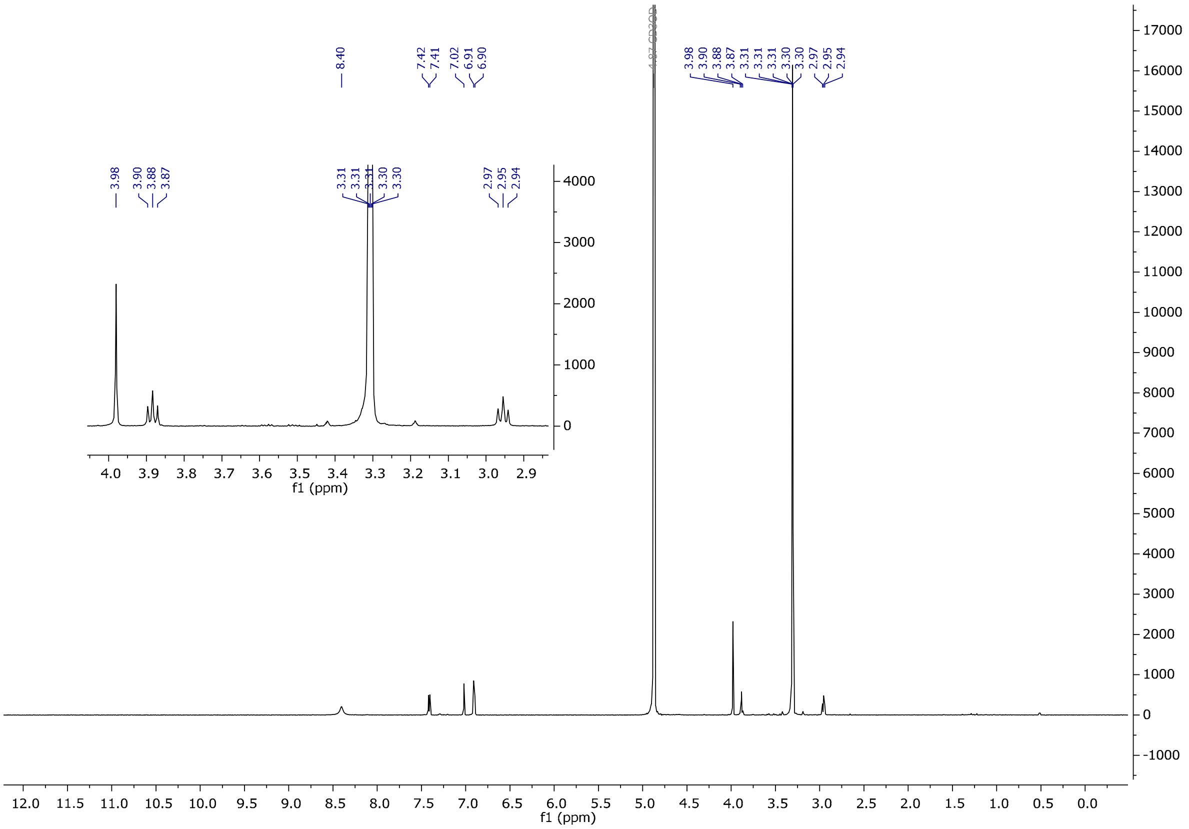

S.7.24 ^{1}H NMR spectrum for tsitsikammamine B (**6**) in MeOD-d₄.

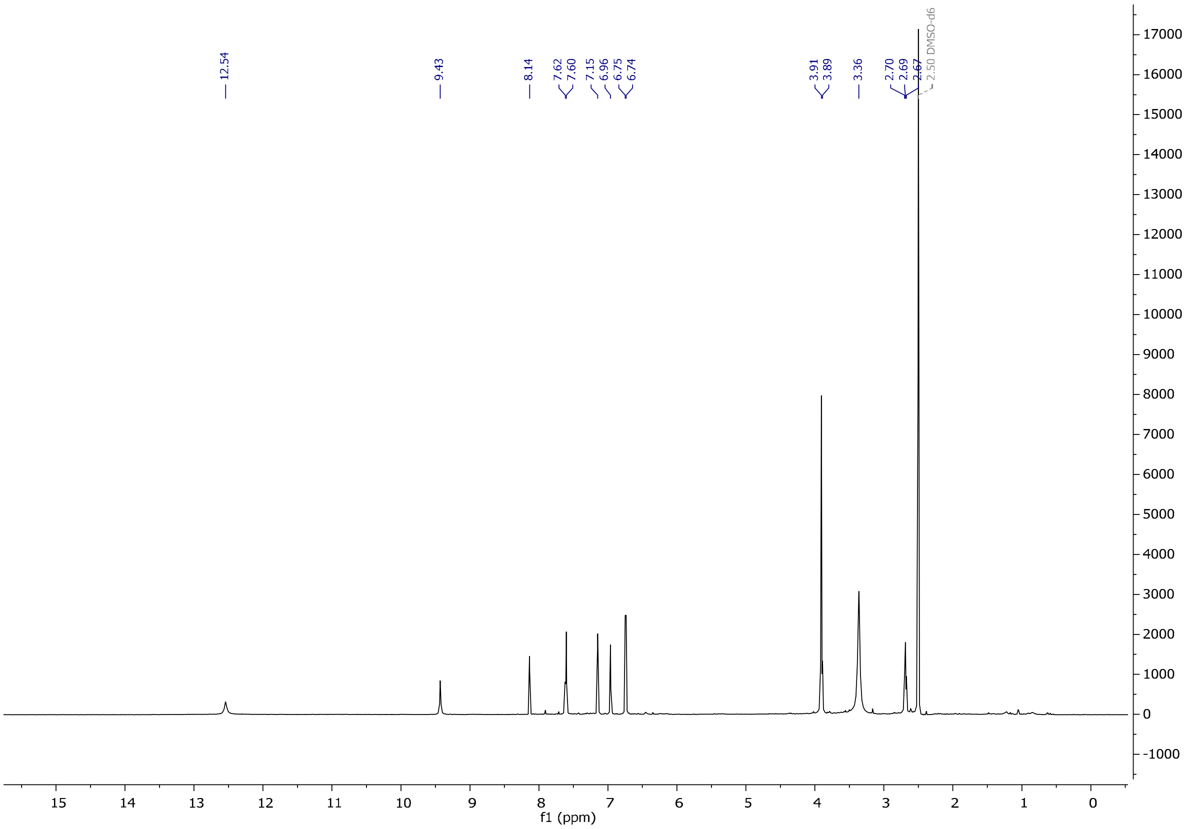

S.7.25 ^{1}H NMR spectrum for tsitsikammamine B (**6**) in DMSO-d₆.

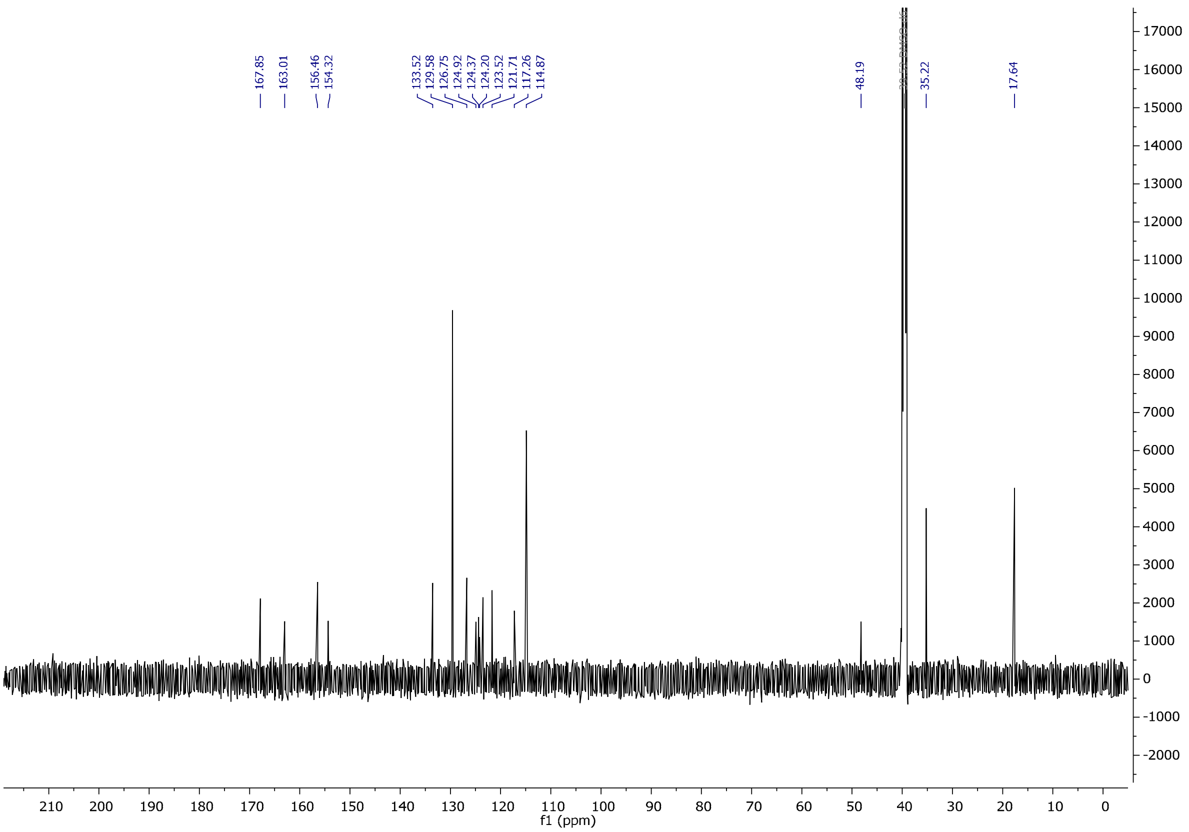

S.7.26 ^{13}C NMR spectrum for tsitsikammamine B (**6**) in DMSO-d$_6$.

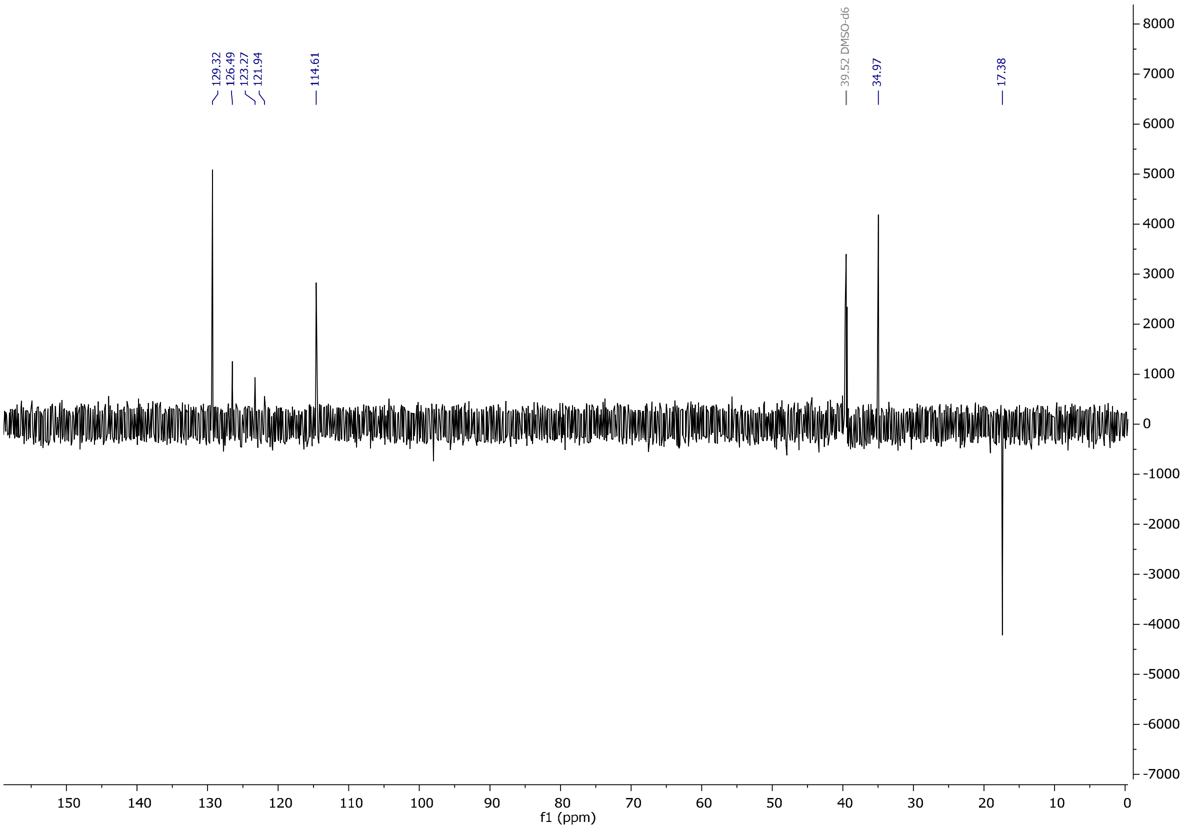

S.7.27 DEPT-135 spectrum for tsitsikammamine B (**6**) in DMSO-d$_6$.

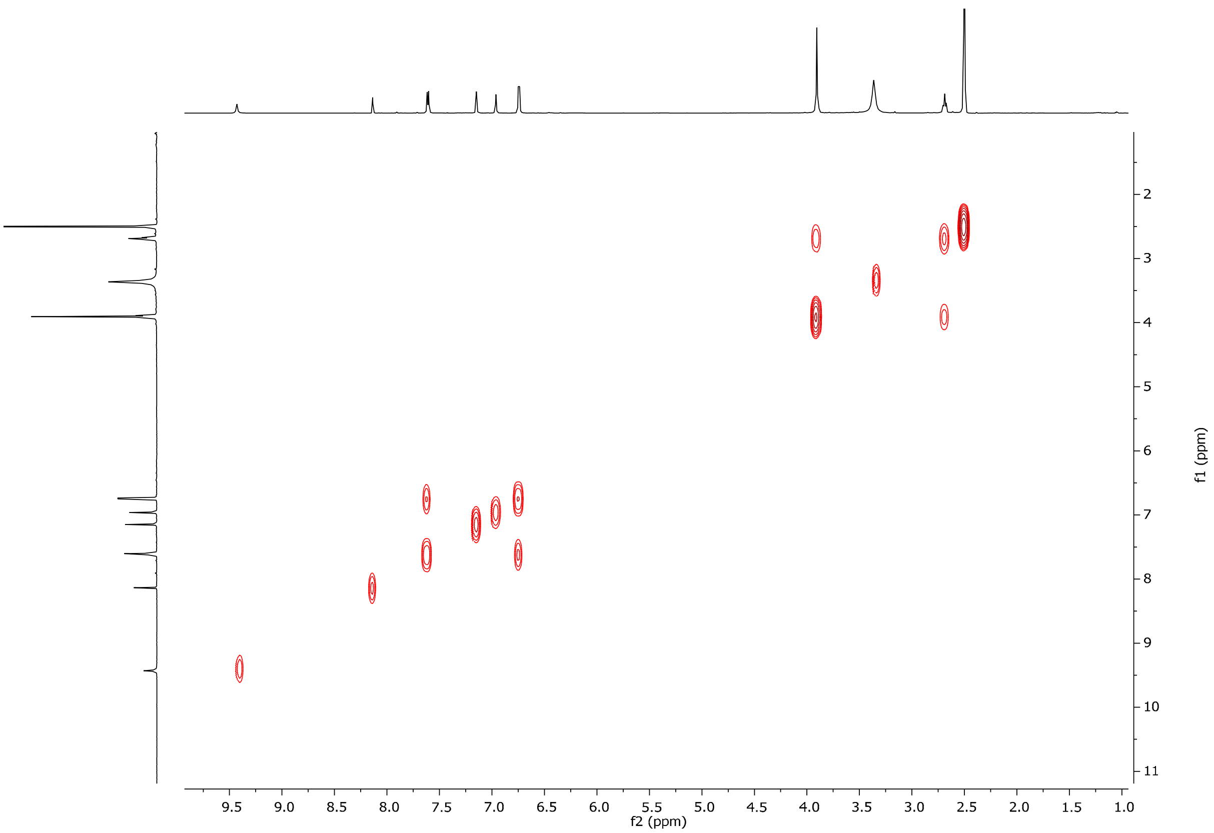

S.7.28 COSY spectrum for tsitsikammamine B (**6**) in DMSO-d6.

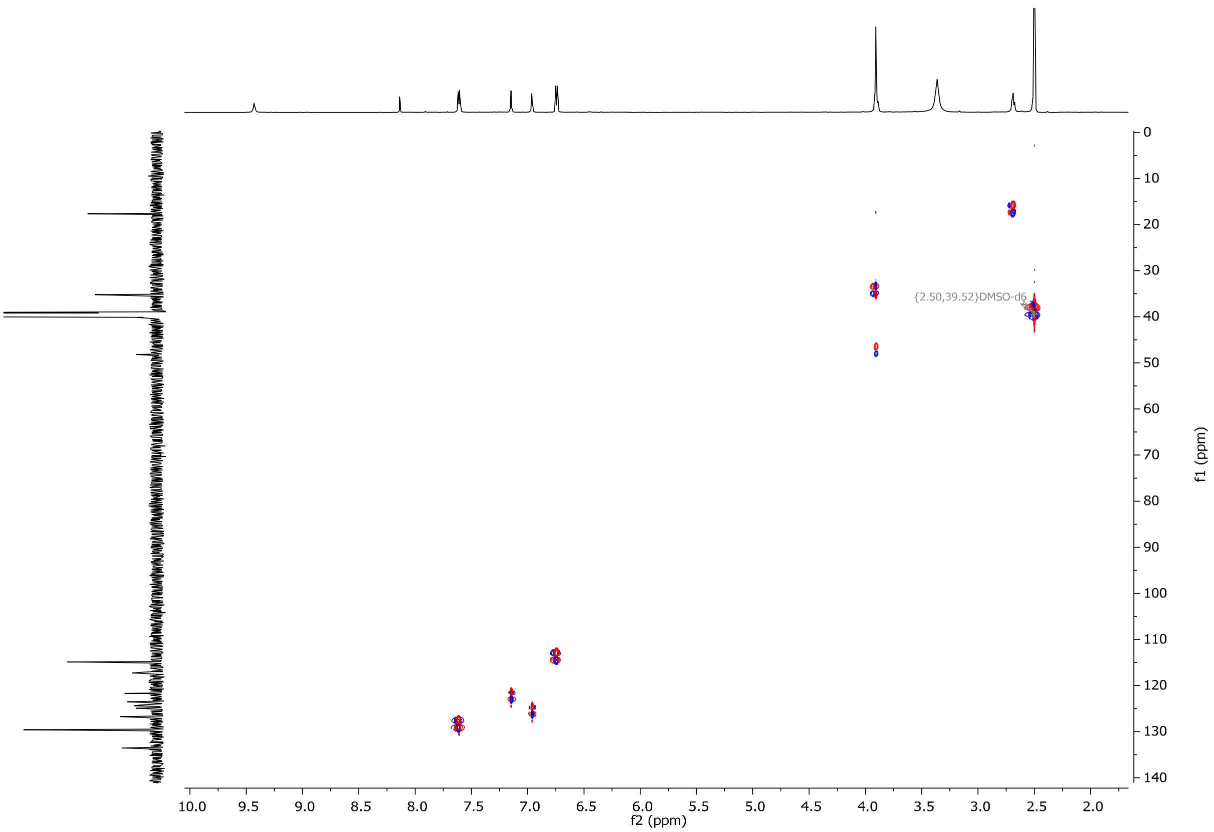

S.7.29 HSQC spectrum for tsitsikammamine B (**6**) in DMSO-d6.

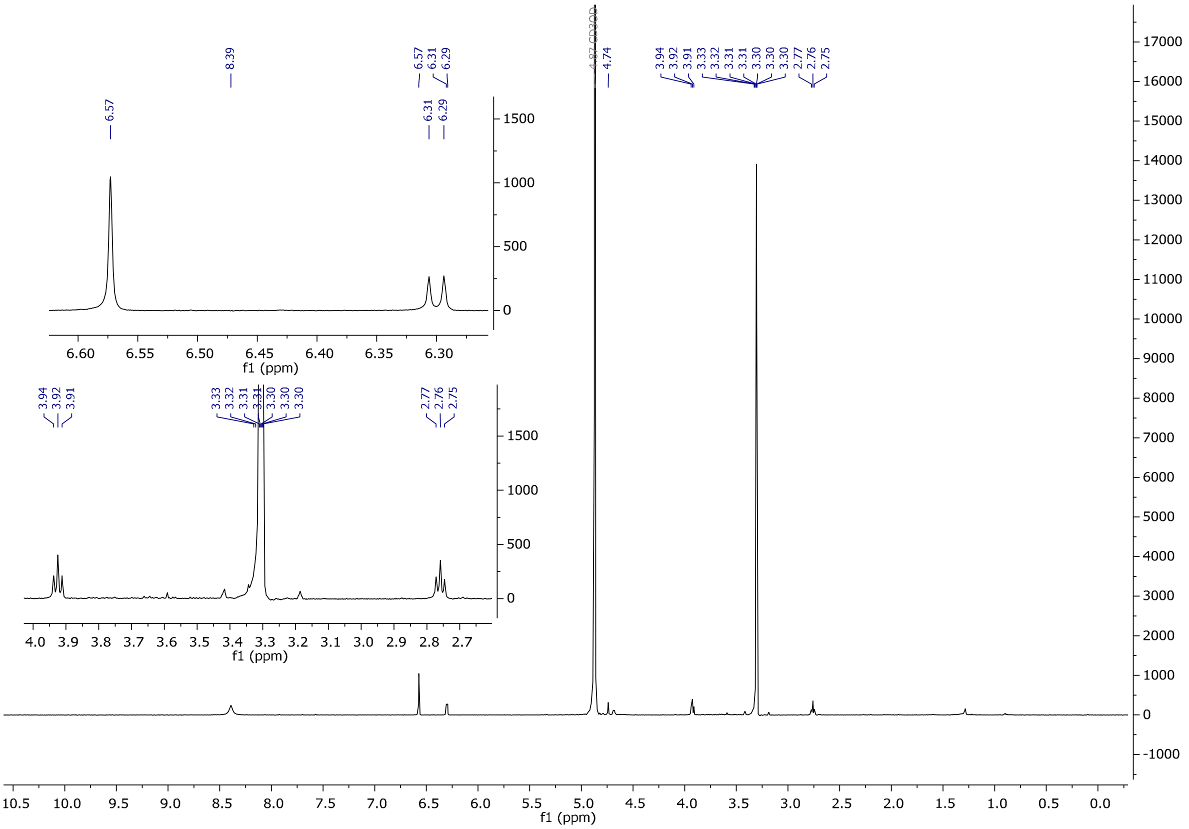

S.7.30 ^{1}H NMR spectrum for 14-bromo-7,8-dehydro-3-dihydrodiscorhabdin C (**7**) in MeOD-d4.

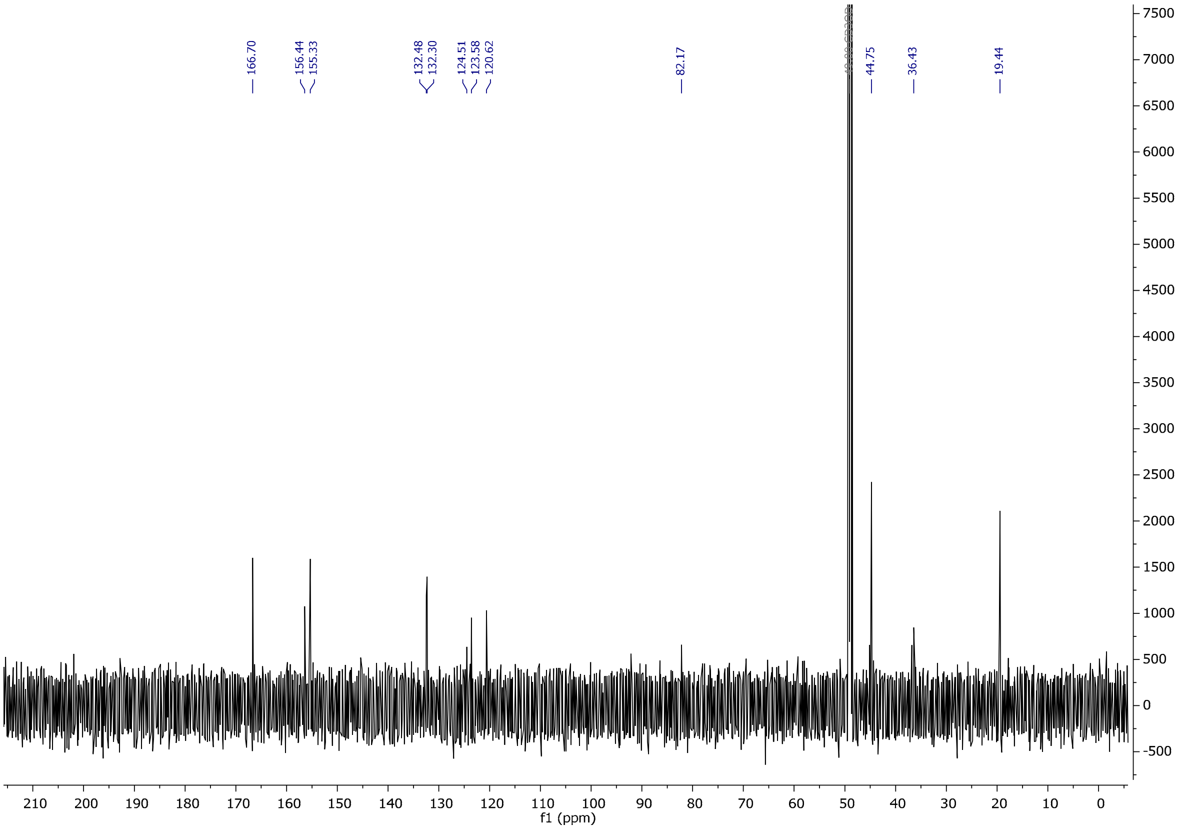

S.7.31 ^{13}C NMR spectrum for 14-bromo-7,8-dehydro-3-dihydrodiscorhabdin C (**7**) in MeOD-d4.

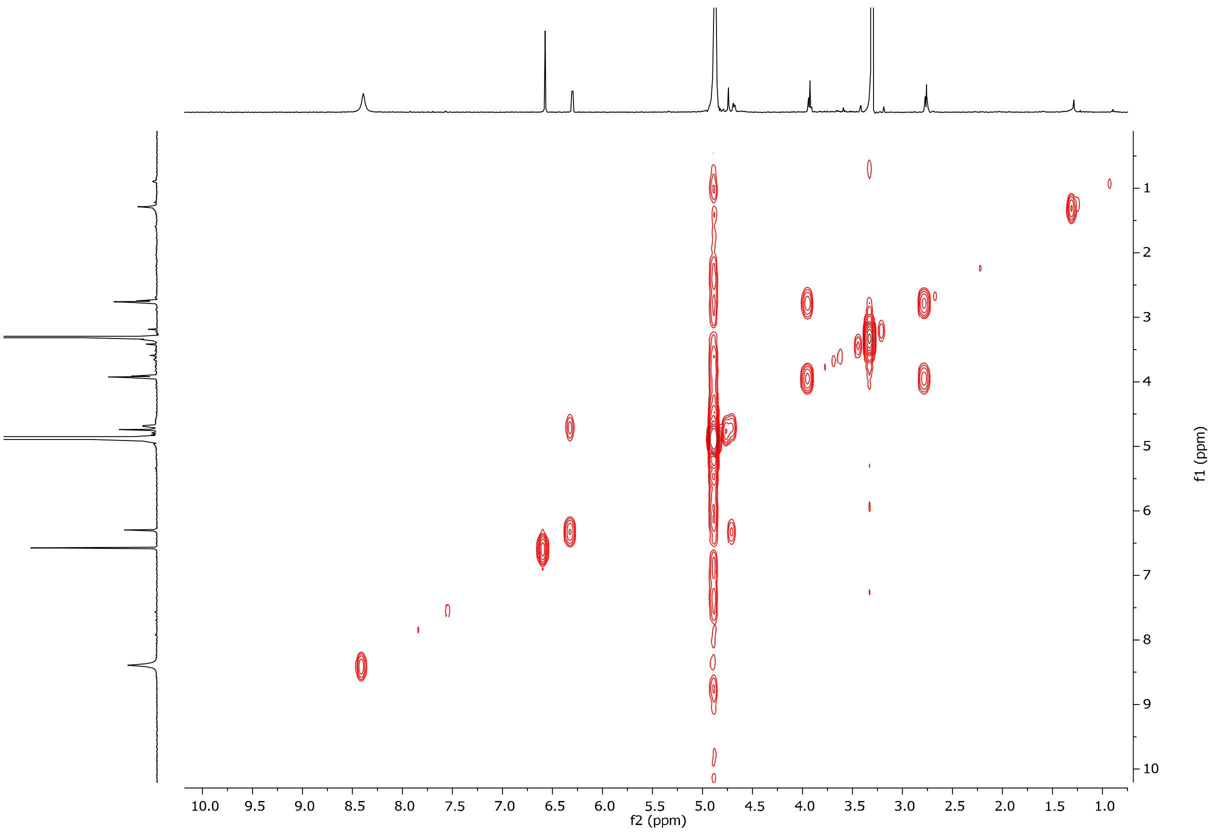

S.7.32 COSY spectrum for 14-bromo-7,8-dehydro-3-dihydrodiscorhabdin C (**7**) in MeOD-d4.

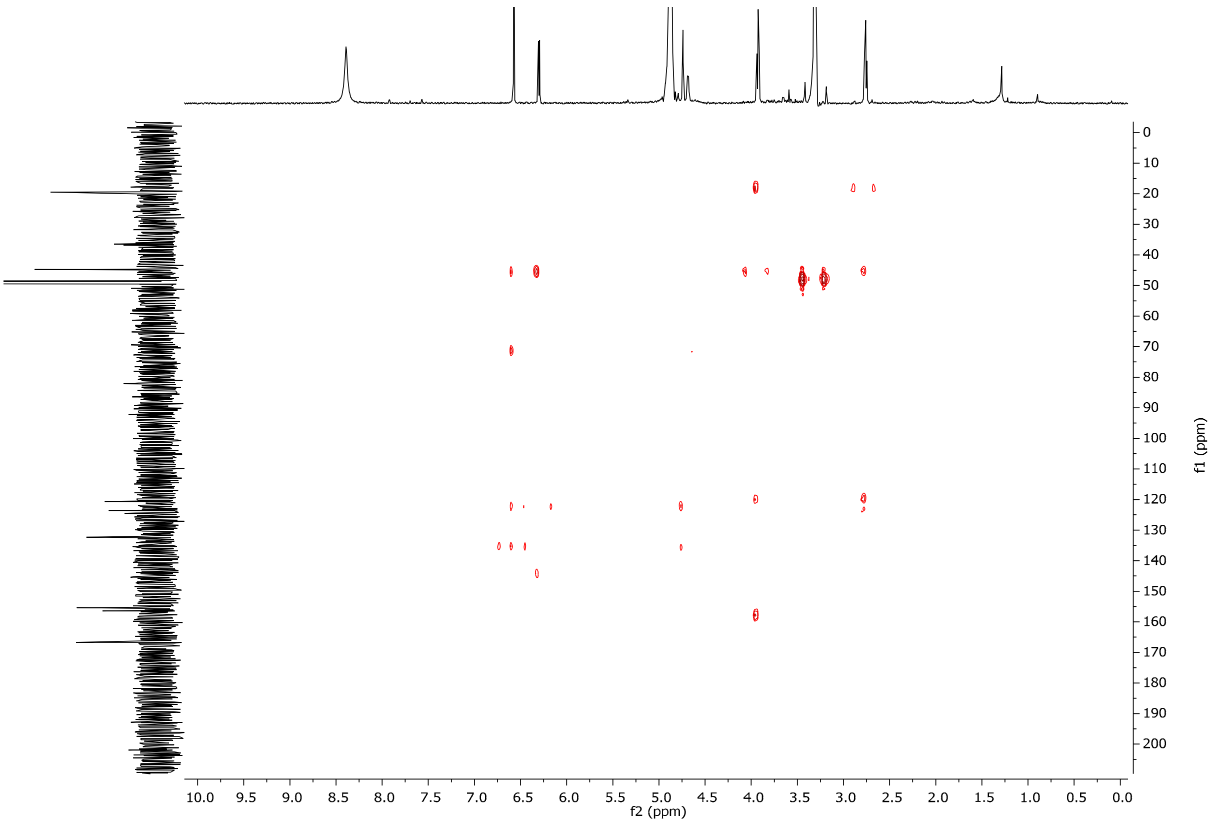

S.7.33 HMBC spectrum for 14-bromo-7,8-dehydro-3-dihydrodiscorhabdin C (**7**) in MeOD-d4.

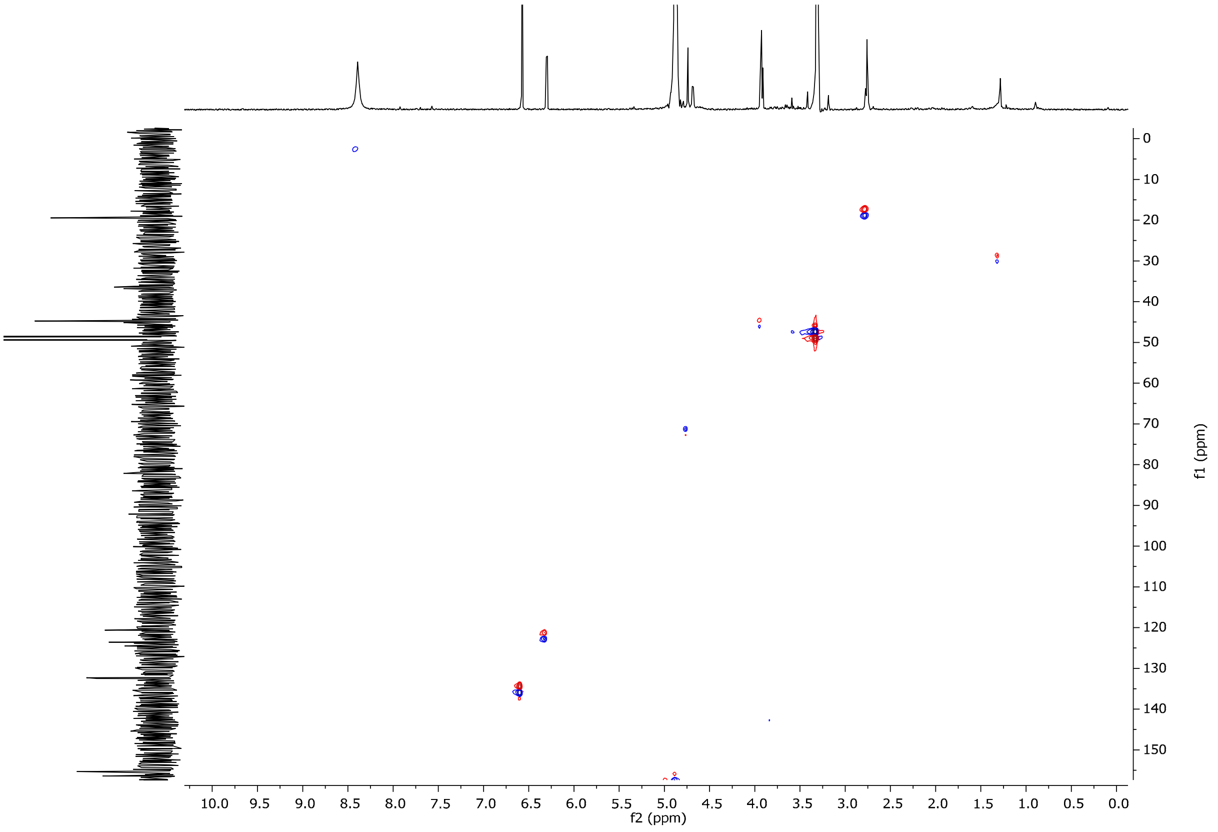

S.7.34 HSQC spectrum for 14-bromo-7,8-dehydro-3-dihydrodiscorhabdin C (**7**) in MeOD-d_4.
